# Quantifying the Influence of Poly(Ethylene glycol) on the Micelle Formation of Nonionic Detergents

**DOI:** 10.1002/cplu.202500380

**Published:** 2025-08-21

**Authors:** Frank Müh, Julia Gätcke, Athina Zouni

**Affiliations:** ^1^ Institute for Theoretical Physics Johannes Kepler University Linz Altenberger Strasse 69 4040 Linz Austria; ^2^ Institute for Biology Humboldt Universität zu Berlin Leonor‐Michaelis‐Haus, Philippstrasse 13 10095 Berlin Germany

**Keywords:** 8‐anilinonaphthalene‐1‐sulfonate, charge–transfer state, critical micelle concentration, fluorescence, poly(ethylene glycol)

## Abstract

The influence of poly(ethylene glycol) (PEG with molecular weights between 400 and 4000) on the critical micelle concentration (CMC) of nonionic detergents with maltose as well as oligo(ethylene glycol) head groups is determined by using 8‐anilinonaphthalene‐1‐sulfonate (ANS) as fluorescence probe. The CMC is found to increase with the concentration of PEG (0%–30% (w/v)) in a way that is determined by the molar concentration of oxyethylene (OE) units and independent of the molecular weight of the added polymer. The effect is explained with the extended conformation of PEG in aqueous solution allowing for an interaction of detergent monomers with individual OE units via their alkyl tails. The fluorescence spectra of ANS are found to exhibit two major emission peaks that are affected in position and intensity by binding to micelles as well as PEG. A model with two conformations of ANS combined with two binding sites in the micelles is used to explain the spectra and their correlation with detergent properties. The shapes of the titration curves are shown to depend on the aggregation number and the equilibrium constant describing binding of ANS to micelles and are analyzed to find that PEG competes with micelles for binding of ANS.

## Graphical abstract

The influence of poly(ethylene glycols) (PEG) with different molecular weights on the critical micelle concentration of nonionic detergents is quantified based on fluorescence spectra of 8‐anilinonaphthalene‐1‐sulfonate (ANS) and found to depend on the concentration of oxyethylene (OE) units rather than polymer length.

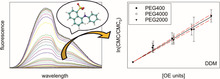

## 1 Introduction

“Models are to be used, not believed.” This quotation comes from an article about micelle structure by F. M. Menger published in 1979, where he states that “Micelles have been the subject of over 2800 publications since 1970”.^[^ ^1^ ^]^ For comparison, “micelles” are the topic of about 114,000 publications in the comparable period from 2015 to 2024 according to Web of Science (as of January 4th, 2025). Of these, nearly 7900 share the topic “polymers”. Apparently, there is an unabated scientific interest in this area. Why is this so? And why are we interested in it?

**Figure 1:**
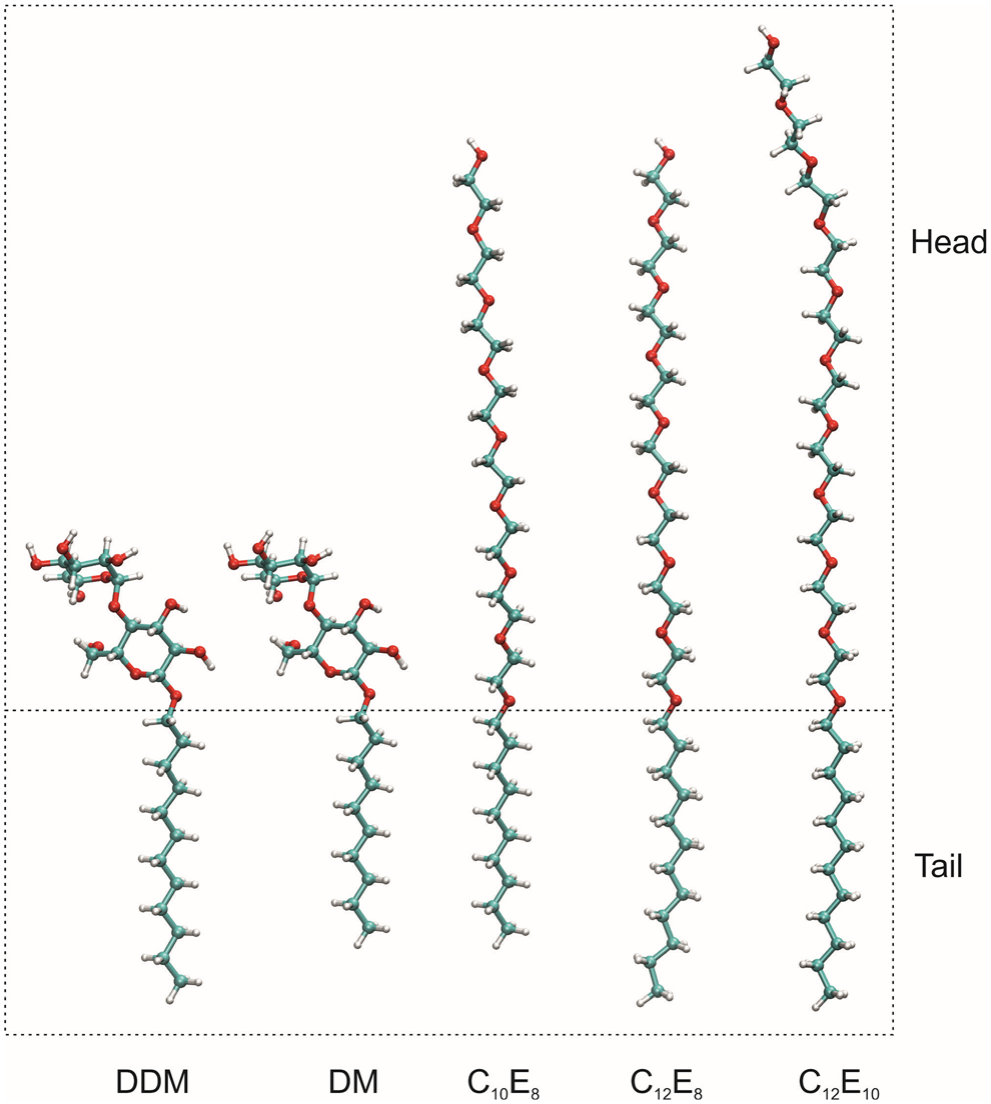
CPK models of the detergents investigated in this work. DDM: dodecyl β‐D‐maltoside; DM: decyl β‐D‐maltoside; C_10_E_8_; Octa(ethylene glycol) monodecylether; C_12_E_8_; Octa(ethylene glycol) monododecylether; C_12_E_10_; Deca(ethylene glycol) monododecylether. In all cases, the hydrophobic tail is an *n*‐alkyl chain. DM and DDM have a maltose moiety as a head group, while the $C_{a}E_{b}$ detergents possess an oligo(ethylene glycol) chain as the hydrophilic part.

The term “micelle” was coined by J. W. McBain at a meeting in 1913^[^ ^1^, ^2^ ^–^ ^3^ ^]^ and refers to an aggregate formed by amphiphilic molecules (such as soap) in solution. Of primary interest for us are amphiphiles with a head‐tail‐structure (see **Figure** **1**), where the head is hydrophilic and the tail hydrophobic, so that micelles are formed in water due to the hydrophobic effect.^[^ ^4^, ^5^ ^–^ ^6^ ^]^ The latter term refers to the low solubility of hydrophobes like hydrocarbons in water and their tendency to aggregate in an aqueous environment.^[^ ^7^ ^]^ The same tendency also causes amphiphiles to enter surfaces or interfaces and lower the surface tension.^[^ ^8^ ^]^ This is why they are called “surfactants”, a blend of “surface active agents”.^[^ ^9^ ^]^ Another name is “detergent,” referring to their detergency properties.^[^ ^10^ ^,^ ^11^ ^]^ We shall use both names synonymously.

It was not until 1920 that McBain first suggested that micelles are only formed above a particular total detergent concentration.^[^ ^3^ ^,^ ^12^ ^]^ Accordingly, the critical micelle concentration (CMC) is to be understood as the minimal total detergent concentration that is necessary for micelles to form and for the detergent solution to exhibit suitable properties such as the solvation of hydrophobes,^[^ ^13^ ^]^ drugs,^[^ ^14^ ^]^ or the solubilization of membrane proteins.^[^ ^15^ ^]^ The CMC can be defined sharply,^[^ ^8^ ^,^ ^16^ ^–^ ^19^ ^]^ but it is not a sharp boundary.

In many applications, detergents occur in mixtures with polymers or proteins.^[^ ^20^ ^]^ Most studies dealing with detergent–polymer interactions are concerned with ionic detergents, where the head group bears a net charge.^[^ ^20^ ^]^ In those cases, one often observes the onset of association of surfactant to the polymer accompanied by micelle formation below the normal CMC at a concentration termed critical association concentration (CAC).^[^ ^21^ ^]^ Note that nonionic detergents, in which the head group is neutral as in the examples shown in Figure 1, rarely show such a distinct interaction with polymers, so that the CAC is not a pertinent concept in these cases. Rather, one observes here an increase of the CMC.^[^ ^22^, ^23^ ^–^ ^28^ ^]^

The interaction of proteins with surfactants is another vital research area. ^[^ ^29^ ^,^ ^30^ ^]^ Our research interest is concentrated on nonionic detergents, since these are indispensable tools in the work with membrane proteins.^[^ ^31^, ^32^, ^33^, ^34^ ^–^ ^35^ ^]^ In fact, many proteins of importance in bioenergetics^[^ ^36^, ^37^, ^38^, ^39^, ^40^ ^–^ ^42^ ^]^ or medicine^[^ ^43^, ^44^, ^45^, ^46^, ^47^, ^48^ ^–^ ^49^ ^]^ are transmembrane protein complexes that span a lipid bilayer in their physiological environment. Detergents are required to isolate the proteins for a structural characterization either by X‐ray crystallography (XRC)^[^ ^31^ ^,^ ^35^ ^,^ ^50^ ^,^ ^51^ ^]^ or cryo‐electron microscopy (cryo‐EM).^[^ ^52^ ^]^ In this context, we will use the term solubilization in the sense of making the protein soluble in water and keeping it in solution. To solubilize a membrane protein, the detergent has to form a belt around the originally membrane‐facing parts of the protein´s surface to shield these hydrophobic regions from the aqueous environment.^[^ ^53^ ^]^ These belts mirror micelles in that the detergent tails preferentially face inwards and the heads outwards, but they are structurally distinct. Yet, the assembly of the belts is governed by the same physical principles as micelle formation. Accordingly, a minimal detergent concentration is required for belt assembly and, hence, membrane protein solubilization. We refer to the minimal detergent concentration required to keep the membrane protein monodisperse in an aqueous solution as critical solubilization concentration (CSC).^[^ ^54^ ^]^ The CSC is similar to the CMC, but is expected to depend on the protein concentration and to be actually larger than the CMC.^[^ ^15^ ^,^ ^27^ ^,^ ^54^ ^]^ However, the precise relation between CMC and CSC remains to be clarified.

In order to crystallize a protein for XRC, one has to add a precipitating agent to drive the solution into the supersaturated regime.^[^ ^55^ ^]^ Often, a water‐soluble polymer like poly(ethylene glycol) (PEG) is employed for this purpose.^[^ ^55^, ^56^ ^–^ ^57^ ^]^ Note that PEG is also used to purify proteins by means of fractional precipitation.^[^ ^58^, ^59^, ^60^ ^–^ ^61^ ^]^ The influence of PEG on the solubility of proteins has been discussed in the literature, but there is no consensus about the physical mechanisms.^[^ ^58^ ^,^ ^62^, ^63^, ^64^ ^–^ ^65^ ^]^

In the case of membrane proteins, one has to take into account that PEG affects—in addition to the solubility of the protein itself—the self‐assembly of the detergent into micelles or belts and, in this way, may indirectly influence the aggregation behavior of the entity to be crystallized. Systematic studies of how PEG shifts the CMC of nonionic detergents are astonishingly scarce and often restricted to one particular PEG, a limited range of PEG concentrations, and a small set of detergents.^[^ ^22^ ^–^ ^26^ ^]^ These limitations are understandable in view of the laboriousness of systematic measurement series.

Our own attempts to shed light on the polymer effect on micellization started in 2015, when we studied the influence of various PEGs on a series of alkyl β‐D‐maltosides. However, only the data pertaining to PEG2000 were analyzed and published.^[^ ^27^ ^]^ The CMC was measured by employing the fluorescence enhancement of 8‐anilinonaphthalene‐1‐sulfonate (ANS) when it enters a medium that is less polar than water, like the interior of a micelle.^[^ ^66^ ^,^ ^67^ ^]^ The key finding of this earlier study was that PEG2000 increases the CMC of all alkyl β‐D‐maltosides according to Equation (1),^[^ ^27^ ^]^

(1) $$\ln\;\left(\frac{\mathrm{CMC}}{{\mathrm{CMC}}_{0}}\right)=k_{P}\chi$$

where CMC and CMC_0_ are, respectively, the CMC in the presence and absence of PEG, $k_{P}$ is a constant, and χ the PEG concentration in %(w/v). Equation (1) has the same form as the corresponding equation describing effects of salts on the CMC, where the constant is referred to as the ‘salt (effect) constant’.^[^ ^68^, ^69^, ^70^ ^–^ ^71^ ^]^ Accordingly, we termed $k_{P}$ the ‘polymer constant’.^[^ ^27^ ^]^ Note, however, that while the salt constant is usually negative, $k_{P}$ is positive. It should be mentioned that our experiments were not performed in pure water but in a buffer system suitable for the crystallization of the photosynthetic membrane protein complex photosystem II (PSII).^[^ ^41^ ^]^ The polymer constant depends on the buffer conditions and may differ even for the same detergent. In extreme cases, PEG may have a nonmeasurable effect on the CMC as observed by Hitscherich et al.^[^ ^24^ ^]^

It took some time until we published the remaining data pertaining to PEGs with molecular weights from 400 to 8000. The key result was that the PEG effect can be described by Equation (2),^[^ ^28^ ^]^

(2) $$\ln\;\left(\frac{\mathrm{CMC}}{{\mathrm{CMC}}_{0}}\right)=k\;c_{\mathrm{OE}}$$

where k is another positive constant and $c_{\mathrm{OE}}$ is the molar concentration of oxyethylene (OE) units. Unexpectedly, k was found to be very similar for PEG polymers with different molecular weights and thus determined by the overall concentration of OE units irrespective of their topological connection.

The recent work yielding Equation (2) raised a number of problems,^[^ ^28^ ^]^ which we address in the present paper: 1) The measurement series were afflicted with some statistical spread and possibly also systematic errors, which we could not attribute to certain reasons, because the experimental data were collected a decade ago. In order to confirm Equation (2), we decided to mitigate the errors by repeating the experiments, focusing on two different PEGs with a significantly different molecular weight, PEG400 and PEG4000. 2) The CMC shift was interpreted in terms of a model in which PEG affects mainly the solubility of the alkyl tail in the aqueous phase. Reviewers gave consideration to the role of the head group. We address this concern by investigating a series of detergents that not only differ in the length of the alkyl tail but in the type and size of the head group (see Figure 1). To this end, we added detergents with oligo(ethylene glycol) head groups to the list of study subjects. In particular, C_12_E_8_ came into focus in recent years as it allows growing a crystal type of PSII^[^ ^72^ ^]^ that gained in importance for elucidating the dynamical mechanism of water splitting in oxygenic photosynthesis.^[^ ^73^, ^75^ ^–^ ^76^ ^]^ 3) The theory underpinning the data analysis relied on the use of the mole fraction as concentration unit and a decomposition of the free energy of the solution that employs the entropy of mixing. Here, we use the more common molar concentration unit and employ Ben‐Naim´s concept of the pseudo‐chemical potential (PCP)^[^ ^77^ ^]^ that is rooted in statistical thermodynamics.^[^ ^78^ ^,^ ^79^ ^]^ Using these concepts, it is possible to boil down the theory of cosolute effects on the CMC (see Section ST1, Supporting Information) to Equation (3),

(3) $$\ln\;\left(\frac{\mathrm{CMC}}{{\mathrm{CMC}}_{0}}\right)=g_{\mathrm{mic}}-g_{\mathrm{mic}}^{0}$$

where $g_{\mathrm{mic}}$ and $g_{\mathrm{mic}}^{0}$ are the PCP differences between detergent micelles and monomers (in units of the thermal energy) in the presence and absence, respectively, of PEG. The further interpretation of the polymer constant k in Equation (2) will be based on Equation (3). It should be noted nonetheless that Equation (3) owes its simplicity to the neglect of micelle polydispersity, that is, the assumption that the aggregation number of detergent micelles is sharply defined and not affected by PEG. In the spirit of the quotation from Menger,^[^ ^1^ ^]^ it is only to the extent that micellar size polydispersity is not of concern that our model can be used to understand the CMC shift, but it does not have to be believed.

## 2 Results and Discussion

### 2.1 Definition and Measurement of the CMC

In the majority of the literature, the CMC is defined empirically based on breaking points in experimental titration curves.^[^ ^80^ ^]^ Phillips^[^ ^16^ ^]^ proposed to define the CMC more sharply as the concentration that corresponds to the maximum change in the gradient of a plot of the measured quantity ϕ against the total detergent concentration x, given by a zero third derivative^[^ ^18^ ^]^

(4) $${\left(\frac{d^{3}\phi }{dx^{3}}\right)}_{x\;=\;\mathrm{CMC}}=0$$

Al‐Soufi et al.^[^ ^18^ ^]^ noted that various experimental methods, which differ in the measured quantity ϕ, may give inconsistent results and employed the ‘Phillips‐condition’ to the detergent monomer concentration y in order to have a consistent reference for all methods.

(5) $${\left(\frac{d^{3}y}{dx^{3}}\right)}_{x\;=\;\mathrm{CMC}}=0$$

Then, the problem is to find out how the zero third derivative of y is related to that of the used experimental quantity ϕ. Al‐Soufi et al.^[^ ^18^ ^]^ employed Equation (5) in an empirical concentration model, while Bothe et al.^[^ ^19^ ^]^ used it for a more strict thermodynamic analysis of nonionic detergents based on a mass‐action model, in which a fixed aggregation number *m* of micelles is assumed. We recently learned that a similar analysis was performed earlier by Corkill et al.,^[^ ^17^ ^]^ but without an in‐depth derivation. Thus, we refer readers interested in mathematical details to Bothe et al.^[^ ^19^ ^]^ (see also the book by Moroi^[^ ^8^ ^]^). The advantage of this procedure is that it yields—within the limits of the mass‐action model—an accurate relationship between the CMC and the micellization free energy $g_{\mathrm{mic}}$ (which is, more precisely, the PCP difference between detergent micelles and monomers divided by the thermal energy $k_{B}T$; cf. Section ST1, Supporting Information)

(6) $$g_{\mathrm{mic}}=\frac{m-1}{m}\ln\left(\mathrm{CMC}L_{1}\right)+\tau_{m}+\frac{5}{2m}\ln\;m$$

In Equation (6), $L_{1}$ is a volume‐like quantity related to the thermal motion of a detergent monomer (see Section ST1, Supporting Information), $\tau_{m}$ is a complicated function of the aggregation number m (see Equation (S84), Supporting Information), and the last term is—like $L_{1}$—related to the statistical mechanical treatment of kinetic energy.^[^ ^79^ ^]^ In the following, we will avoid these complications by assuming that PEG does not affect m. The additional terms then drop out, if the CMC is referred to PEG‐free conditions, and we arrive at Equation (3).

**Figure 2:**
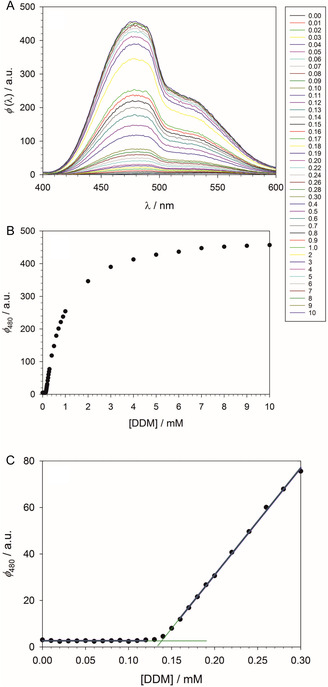
Determination of the CMC of DDM in 100 mM PIPES (pH 7.0), 5 mM CaCl_2_ at 19 °C by titration of the fluorescence of 10 µM ANS. A) Fluorescence spectra in the wavelength range *λ* = 400–600 nm (excitation wavelength: 354 nm). The legend indicates the DDM concentrations in mM. B) Dependance of the fluorescence at 480 nm on the detergent concentration, [DDM]. C) Determination of the breaking point indicating the CMC. The blue lines result from linear regressions of the respective parts of the titration curve. The green lines are extrapolations of the regression lines to their point of intersection, which corresponds to the breaking point. The extrapolation is done analytically as described in the text. Figures and regressions were made with SigmaPlot 13 (2014 Systat Software Inc.).

Bothe et al.^[^ ^19^ ^]^ have shown that if ϕ is the fluorescence intensity of ANS,^[^ ^66^ ^,^ ^67^ ^]^ the breaking points defined by (Equation 4) and (5) coincide, so that the fluorescence enhancement method is ideally suited to measure the CMC as defined by the ‘Phillips condition’ and to employ (Equation 3) and (6). The determination of the CMC by this method is illustrated in **Figure** **2** for the example of DDM in PEG‐free buffer. Upon addition of DDM to the buffer, the fluorescence intensity of ANS increases, indicating binding of ANS to micelles (Figure 2A). At higher detergent concentrations—well above the CMC, where the fluorescence enhancement due to micelle formation levels off (Figure 2B)—ANS exhibits a fluorescence spectrum with a major peak at 480 nm and a shoulder at ≈530 nm. These spectra are reminiscent of fluorescence spectra of ANS binding to hydrophobic patches of proteins or biological membranes.^[^ ^81^, ^82^, ^83^ ^–^ ^85^ ^]^ They are, however, different from the spectra reported by De Vendittis et al.,^[^ ^66^ ^]^ which do not show the shoulder and refer to ANS bound to micelles of sodium dodecyl sulfate (SDS). The fluorescence spectra of ANS will be further analyzed below. In the context of determining the CMC, the relevant aspect is that the occurrence of the shoulder in the fluorescence spectrum does not compromise the suitability of ANS as an indicator of micelle formation. The fluorescence enhancement can be probed by analyzing the fluorescence intensity at 480 nm, $\phi_{480}$ (Figure 2B,C).

To quantify the CMC, we employ partial linear regression (Figure 2C). Below the CMC, the fluorescence intensity is constant at a low level $\phi_{480}(x)=c_{0}$, and the constant can be determined by a linear fit of the corresponding data points. Right above the CMC, the fluorescence intensity can be fit to a linear function of the form

(7) $$\phi_{480}\left(x\right)=a_{0}+b_{1}x$$

If we denote the (arbitrary) unit of $\phi_{480}(x)$ by a.u. and give the total detergent concentration x in mM, the constants $a_{0}$ and $c_{0}$ have the unit a.u., while $b_{1}$ has the unit a.u./mM. The values of these constants for all measured titration curves are collocated in Table S1 (Supporting Information). The given standard errors originate from linear regression and refer to a single titration curve in each case. With the assumption that the point of intersection of the two lines (green in Figure 2C) occurs at the point x that corresponds to the zero third derivative of the $\phi_{480}(x)$‐curve, we can compute the CMC according to

(8) $$\mathrm{CMC}=\frac{c_{0}-a_{0}}{b_{1}}$$

If $\text{Δ}a_{0}$, $\text{Δ}b_{1}$, and $\text{Δ}c_{0}$ are the standard errors of $a_{0}$, $b_{1}$, and $c_{0}$, respectively, the relative error of the CMC due to uncertainties in one titration curve follows as

(9) $$\frac{\text{Δ}\mathrm{CMC}}{\mathrm{CMC}}=\frac{\text{Δ}a_{0}+\text{Δ}c_{0}}{c_{0}-a_{0}}+\frac{\text{Δ}b_{1}}{b_{1}}$$

The obtained CMC values and standard errors ΔCMC are presented in **Table** **1**, while literature data are compiled in Tables S2 and S3 (Supporting Information).

**Table 1:** Experimentally determined CMC values (in mM) in dependance of the PEG concentration χ (in % (w/v)).

χ	DDM			DM		C_10_E_8_		C_12_E_8_		C_12_E_10_	
	PEG400	PEG2000	PEG4000	PEG400	PEG4000	PEG400	PEG4000	PEG400	PEG4000	PEG400	PEG4000
0	0.139 ± 0.005	–	–	1.86 ± 0.10	–	0.90 ± 0.09	–	0.071 ± 0.017	–	0.029 ± 0.002	–
5	0.177 ± 0.007	0.183 ± 0.007	0.173 ± 0.004	1.95 ± 0.08	2.13 ± 0.15	1.11 ± 0.06	1.16 ± 0.05	0.093 ± 0.004	0.096 ± 0.005	0.032 ± 0.003	0.040 ± 0.002
10	0.206 ± 0.012	0.201 ± 0.007	0.206 ± 0.008	2.23 ± 0.21	2.36 ± 0.35	1.26 ± 0.14	1.31 ± 0.14	0.113 ± 0.007	0.126 ± 0.006	0.047 ± 0.004	0.047 ± 0.004
15	0.239 ± 0.013	0.24 ± 0.04	–	2.75 ± 0.10	2.67 ± 0.15	1.38 ± 0.10	1.25 ± 0.17	0.135 ± 0.014	0.135 ± 0.024	0.052 ± 0.007	0.045 ± 0.005
20	0.30 ± 0.02	–	–	3.07 ± 0.15	2.77 ± 0.13	1.53 ± 0.08	1.34 ± 0.14	0.168 ± 0.018	0.164 ± 0.023	0.071 ± 0.008	0.049 ± 0.012
25	–	–	–	3.37 ± 0.16	–	1.76 ± 0.06	1.74 ± 0.31	0.193 ± 0.013	0.144 ± 0.085	0.080 ± 0.013	0.062 ± 0.011
30	–	–	–	3.9 ± 0.5	–	1.86 ± 0.32	1.64 ± 0.37	0.238 ± 0.032	–	0.081 ± 0.011	0.054 ± 0.020

The CMC values we obtain in the present work for DM and DDM are larger than in our earlier work,^[^ ^27^ ^]^ but closer to literature values (Table S3, Supporting Information). As can be seen from Figure S2 (Supporting Information), the logarithm ln (CMC/mM) follows the expected linear dependance on the number a of carbon atoms in the alkyl tail. The deviation of our present from our earlier data is clearly observable but still close to the usual spread of measured CMC values. We note that this spread cannot be traced back solely to the different experimental methods used to determine the CMC (cf. footnotes to Table S2, Supporting Information), but likely arises from differences in experimental conditions such as buffer composition, temperature, and chemical source. In the present work, we took care to keep the temperature in the range (19±1) °C, which is lower than the normal room temperature. A possible reason for the lower CMC values obtained by us earlier under the same experimental conditions^[^ ^27^ ^]^ could be the hydrolysis of the ether linkage, which would produce long‐chain alcohols that tend to lower the CMC. The good match of our new data with the literature suggests that problems of this sort are minimized in the present work and that the lower temperature is a minor problem.

A linear dependance of the logarithm ln (CMC/mM) on the number a of carbon atoms in the alkyl tail is also observed for oligo(ethylene glycol) monoalkyl ethers, $C_{a}E_{b}$ (Figure S3, Supporting Information). In the following, we will denote the head groups of these detergents as OEG groups for ‘oligo(ethylene glycol)’ to distinguish them from the separate PEG molecules discussed below. In both cases, we will refer to the ethylene glycol subunits as OE units for ‘oxyethylene’. Thus, b is the number of OE units in the OEG head. The distinction between ‘oligo’ and ‘poly’ is made here merely to better distinguish the detergents from the polymers. It has no physical meaning, however, since the average number of OE units in PG400, for example, is 8.761^[^ ^28^ ^]^ and thus similar to b for the detergents. For PEG4000, the number is 90.391,^[^ ^28^ ^]^ so that the term ‘poly’ is more adequate in this case.

Our CMC values for C_10_E_8_ and C_12_E_8_ (Table 1) are in good agreement with literature data (Table S2 and Figure S3, Supporting Information), whereas our value for C_12_E_10_ is somewhat too low. For this type of detergent, it makes sense to ask for the dependance of ln (CMC/mM) on the number b. A correlation can be found only for fixed a, where the slope is larger for a=10 than for a=12 (Figure S4, Supporting Information). This observation is qualitatively in agreement with the results of Berthod et al.,^[^ ^86^ ^]^ who found the slope to increase with decreasing a. In fact, they found positive slopes for a=8 and a=12, but a negative slope for a=16. On the scale of Figure S4 (Supporting Information), it is even more apparent that our CMC value for C_12_E_10_ is too low. We note that we found only one CMC value for C_12_E_10_ in the literature (0.09 mM),^[^ ^87^ ^]^ referring to a detergent mixture, where b is just an average. While this CMC value fits well to the values for other detergents with a=12, the possibility should be taken into account that many data pertaining to older works do not refer to pure detergent compounds.

### 2.2 Effect of PEG on ANS Fluorescence and the CMC

**Figure 3:**
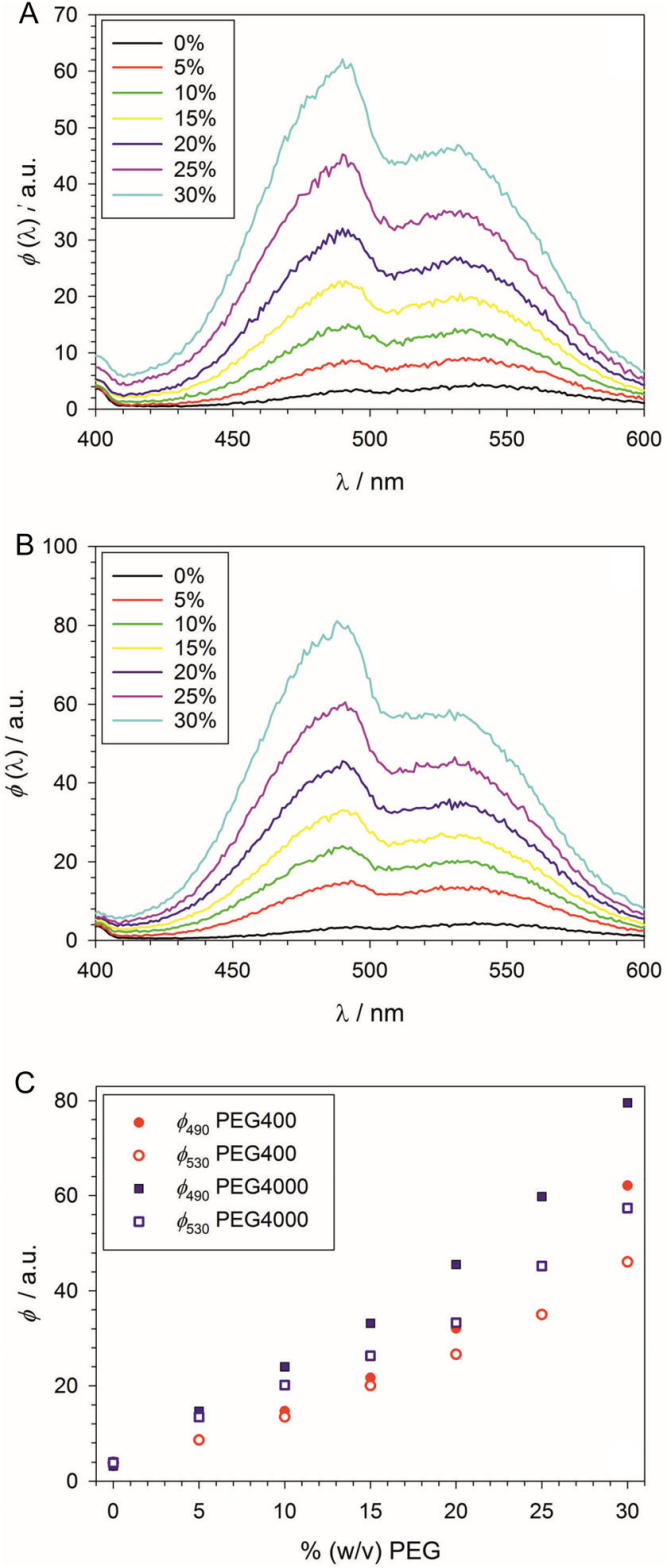
Fluorescence spectra in the wavelength range *λ* = 400–600 nm of 10 µM ANS in 100 mM PIPES (pH 7.0), 5 mM CaCl_2_ at 19 °C in the absence of detergent (excitation wavelength: 354 nm). A) Dependance of the spectra on the addition of PEG400. B) Dependance of the spectra on the addition of PEG4000. C) Dependance of the fluorescence intensities at the peak maxima (490 and 530 nm) on the PEG concentration. Plots made with SigmaPlot 13 (2014 Systat Software Inc.).

PEG has an influence on the fluorescence spectrum of ANS (**Figure** **3**). In buffer, ANS shows a weak fluorescence with two emission maxima around 490 and 530 when excited at 354 nm. The choice of the excitation wavelength was motivated by a comparison of the absorption spectra of ANS in buffer without detergent or with detergent (DDM) at a concentration above the CMC (Figure S5, Supporting Information). Addition of DDM caused a redshift of the first major absorption peak with an isosbestic point around 354 nm (inset to Figure S5, Supporting Information) reflecting binding of ANS to micelles. This point was chosen as excitation wavelength to minimize a variation of the excited state population (and hence emission intensity) with detergent concentration. The isosbestic point suggests the presence of two conformations of ANS with strongly overlapping absorption peaks, whose relative population changes upon binding of ANS to micelles. It is well possible that the two emission peaks seen in the fluorescence spectra correspond to these two conformations and that the choice of the isosbestic point for excitation is the reason why both emission peaks are observed in contrast to many spectra reported in the literature.

Addition of PEG to the buffer causes both emission peaks of ANS to increase in intensity, with the peak at 490 nm becoming more intense than the one at 530 nm with increasing PEG concentration (Figure 3). Notably, this effect is more pronounced for PEG4000 than for PEG400 at the same PEG concentration (Figure 3C). This result not only shows that ANS binds to PEG but also that the longer PEG chains offer more suitable ‘binding sites’ for ANS than the shorter chains. The same trend is observed when the peak intensities are plotted as a function of the concentration of OE units, $c_{\mathrm{OE}}$, of the added polymer (Figure S6, Supporting Information). We note that the conversion factors from %(w/v) to $c_{\mathrm{OE}}$ for PEG have been determined in our earlier work.^[^ ^28^ ^]^ They are 0.2168 for PEG400 and 0.2260 for PEG4000 (as well as 0.2250 for PEG2000 also needed below).

**Figure 4:**
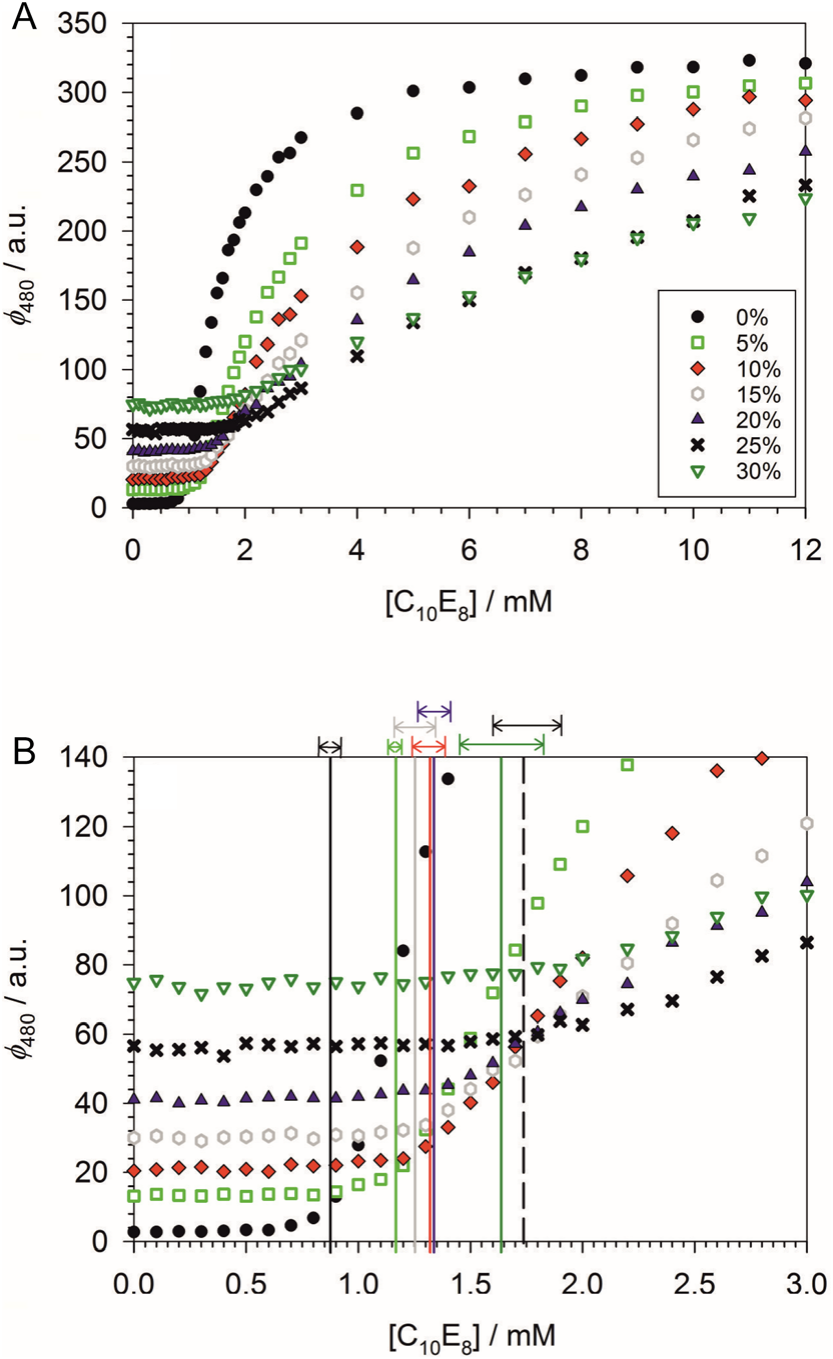
Determination of the CMC of C_10_E_8_ in 100 mM PIPES (pH 7.0), 5 mM CaCl_2_ at 19 °C at different concentrations of PEG4000 by titration of the fluorescence of 10 µM ANS (excitation wavelength: 354 nm). The legend indicates the PEG concentrations in %(w/v) and defines the color code. A) Dependance of the fluorescence at 480 nm on the detergent concentration, [C_10_E_8_]. B) Titration curves in the region of the breaking points indicating the CMC. The vertical bars mark the corresponding CMC values with the standard error margins displayed by the double arrows. Figure made with SigmaPlot 13 (2014 Systat Software Inc.).

**Figure 5:**
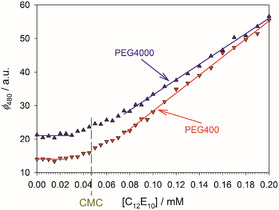
Determination of the CMC of C_12_E_10_ in 100 mM PIPES (pH 7.0), 5 mM CaCl_2_ at 19 °C in the presence of 10% (w/v) PEG400 or PEG4000 by titration of the fluorescence of 10 µM ANS (excitation wavelength: 354 nm). The solid lines result from linear regression of the respective parts of the titration curves (for fitting results, see Table S1, Supporting Information). The vertical dashed line marks the CMC value of 0.047 mM obtained in both cases (cf. Table 1). Figures and fits made with SigmaPlot 13 (2014 Systat Software Inc.).

The addition of PEG causes multiple changes of the fluorescence titration curve, as can be seen from the example of the combination of C_10_E_8_ and PEG4000 shown in **Figure** **4**: 1) The constant fluorescence intensity at 480 nm below the CMC as represented by the fit parameter $c_{0}$ (cf. Table S1, Supporting Information) is increased with increasing PEG concentration. When plotted as a function of the molar concentration of OE units of the added PEG, the increase is nonlinear and larger for PEG4000 than for PEG400 (Figure S7, Supporting Information). In this respect, the behavior of $c_{0}$ mirrors that of $\phi_{490}$ discussed above in accordance with the spectra shown in Figure 3. For DDM, we also investigated the effect of PEG2000. Concerning $c_{0}$, PEG2000 behaves similarly to PEG4000 (Figure S7, Supporting Information). Thus, the effect of added PEG on ANS depends nonlinearly on the chain length of the polymer. 2) The rise of the fluorescence intensity right above the CMC, approximated linearly by Equation (7), is such that the slope $b_{1}$ decreases with increasing concentration of OE units of the added polymer (Figure S8, Supporting Information), that is,the curves are flattened with increasing PEG concentration (Figure 4). Here, again, the polymer size matters, that is, $b_{1}$ is smaller for PEG4000 than for PEG400 at the same concentration of OE units (Figure S8 and Table S1, Supporting Information). As with $c_{0}$, PEG2000 behaves similarly to PEG4000. 3) The breaking point in the titration curve indicating the CMC is shifted to higher values with increasing PEG concentration. Here, we observe that the polymer size does not matter, that is, the CMC shift for a given detergent is within experimental uncertainties the same for PEG400 and PEG4000 (and also for PEG2000 in the case of DDM), if the polymer concentration in %(w/v) is the same (see the further quantification below). The three effects of PEG on the titration curves and their different dependance on the molecular weight of PEG can best be illustrated with the example of C_12_E_10_ with 10% (w/v) PEG, for which we obtained a CMC value of 0.047 ± 0.004 mM (Table 1). Whereas the CMC value is the same for PEG400 and PEG4000 the initial fluorescence level is higher and the titration curve flatter for PEG4000 than for PEG400 (**Figure** **5**).

Below, in the ‘Further Analysis of Titration Curves,’ we will show that $b_{1}$ can be interpreted as a binding constant, that is, it is–with suitable approximations (cf. Section ST3, Supporting Information)—proportional to the equilibrium constant K for binding of ANS to micelles. In this respect, it is instructive to compare $b_{1}$ for the different detergents at zero PEG concentration (Figure S9, Supporting Information) in order to characterize the binding of ANS to the various micelles. Clearly, $b_{1}$ is larger for detergents with a=12 than for those with a=10. Within each group, $b_{1}$ is larger for the OEG head group with b=8 than for the maltose head group. Comparing C_12_E_8_ and C_12_E_10_, we see that $b_{1}$ is larger for the larger head group. Before interpreting these results, one has to take into account that the various micelles may differ in their tendency to form and their size. Indeed, there is a (nonlinear) correlation between $b_{1}$ and ln (CMC/mM) (Figure S10A, Supporting Information), indicating that the titration curves are steeper if the driving force for micelle formation is higher (or the CMC lower and ln (CMC/mM) more negative). There is also a correlation between $b_{1}$ and the aggregation number m (Figure S10B, Supporting Information), supporting the intuitive idea that ANS binds more favorably to larger micelles. However, before drawing such a conclusion, we have to extract information about the real binding constant K from the data (see below).

At this point one may become suspicious and ask whether there is a contradiction in the data. If ANS binds to PEG with a concomitant increase of the fluorescence intensity (Figure 3), it should also bind to the OEG head groups of the $C_{a}E_{b}$ detergents below the CMC. Shouldn´t we then observe an increase of the fluorescence intensity with increasing detergent concentration below the CMC? The riddle can be solved easily by considering the effective concentration of OE units that can be reached before micelle formation. It is maximal for C_10_E_8_ and corresponds to 8 × CMC = 7.2 mM. A comparison with the PEG effect on $c_{0}$ (Figure S7, Supporting Information) shows that this OE concentration is too small to induce a measurable increase of $\phi_{480}$.

**Figure 6:**
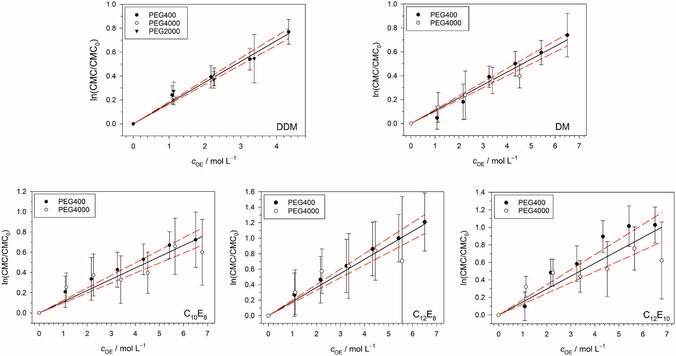
Dependance of $\ln\;(\mathrm{CMC}/{\mathrm{CMC}}_{0})$ for the investigated detergents on the concentration of OE units of the added PEG polymer, $c_{\mathrm{OE}}$ (in mol L^−1^). The solid straight lines follow from linear regression of data points for all types of PEG in each case. The resulting slopes are the polymer constants k according to Equation (2). They are given together with their standard errors in Table 2. The red dashed lines indicate the 95% confidence interval. Figures and fits made with SigmaPlot 13 (2014 Systat Software Inc.).

In order to verify the validity of Equation (2), we have to quantify the observation that the CMC shift does not depend on the molecular weight of PEG. To this end, we plot $\ln\;(\mathrm{CMC}/{\mathrm{CMC}}_{0})$ versus $c_{\mathrm{OE}}$ in **Figure** **6**. It can be seen that for each detergent, the data points lie close to the regression line irrespective of the molecular weight of PEG, supporting the validity of Equation (2) within certain error margins. The spread of the data points around the regression lines is particularly small for DDM and DM, which implies a significant improvement compared to our earlier work.^[^ ^28^ ^]^ The same result is obtained for the $C_{a}E_{b}$ detergents, albeit with a somewhat larger spread, the worst case being C_12_E_10_.

We recall that each data point shown in Figure 6 corresponds to a single titration curve. Certainly, the statistics can be improved by repeating each titration several times. However, in view of the laboriousness of the data measurement procedure, we refrained from such repetitions in the present work (except for initial tests). Nonetheless, we think that the data are sufficient to support Equation (2) at least for the maltosides. In the case of the $C_{a}E_{b}$ detergents, there is room for improvement, particularly for C_12_E_10_, but this is a task for future work.

**Figure 7:**
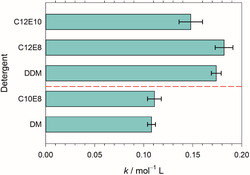
The polymer constants k in Equation (2) due to PEG for the investigated detergents (see also Table 2). The error bars result from the linear regressions shown in Figure 6. The red dashed line separates the detergents with a=10 (bottom) from those with a=12 (top). Figure made with SigmaPlot 13 (2014 Systat Software Inc.).

The slope of a regression line in Figure 6 is the polymer constant k in Equation (2). The polymer constants obtained for the various detergents in the present work are compiled in **Table** **2** and illustrated in **Figure** **7**. It stands out that k is significantly different for detergents with a different number a of carbon atoms in the alkyl tail, being close to 0.1 for a=10 and closer to 0.17 for a=12 (with the unit of k being mol^−1^ L). This result is qualitatively in agreement with our earlier work, where we obtained higher k‐values for longer alkyl chains of the maltosides.^[^ ^27^ ^,^ ^28^ ^]^ However, the polymer constants differ quantitatively. Rescaled from %(w/v) to $c_{\mathrm{OE}}$, we obtained values of 0.1–0.2 for DM and 0.27–0.34 for DDM for various PEGs^[^ ^28^ ^]^ as well as 0.18 for DM and 0.20 for DDM for PEG2000.^[^ ^27^ ^]^ We ascribe these discrepancies to the insufficient accuracy of the older data. In fact, the values obtained earlier for different PEGs^[^ ^28^ ^]^ could still be interpreted such that k depends on the molecular weight of the polymer, and it was the large spread in the data that motivated the present study. Based on the new data, we can now assert that k for the maltosides is indeed independent of the molecular weight and predominantly determined by the concentration of OE units of the added PEG. The same is basically true for the $C_{a}E_{b}$ detergents, but a repetition of the experiments as well as an extension to larger ranges for the values of a and b would be of interest.

**Table 2:** The polymer constants k in Equation (2) due to PEG for the investigated detergents (see also Figure 7). The error margins follow from linear regression of data points for all types of PEG (cf. Figure 6).

Detergent	k in mol^−1^ L
DDM	0.174 ± 0.005
DM	0.108 ± 0.004
C_10_E_8_	0.111 ± 0.007
C_12_E_8_	0.182 ± 0.009
C_12_E_10_	0.148 ± 0.012

Concerning the influence of the detergent head group on the polymer constant, the new data show that there is no significant difference between a maltose and an octa(ethylene glycol) head group. In contrast, there is an apparent difference between C_12_E_8_ and C_12_E_10_. We think that on the basis of the present data, this difference cannot be considered as significant. The error bars shown in Figure 7 originate from the linear regressions shown in Figure 6. These fits are based on all data points in the plot (assuming all PEGs cause the same CMC shift) but do not take into account the error bars depicted in Figure 6. The latter originate from the fits of individual titration curves and merely serve to indicate that all data points could well lie on the same line considering these uncertainties. The fit to determine k yields a different kind of error: It makes a prediction of the standard error based on the spread of the data points, ignoring their individual errors. Thus, the uncertainty of k is probably larger than suggested by the fit. Based on the overall spread of data points, we suspect the results for C_12_E_10_ to be less reliable than those for the other detergents. In particular, the data for PEG4000 seem to be more insecure and are reminiscent of the problems we encountered in our previous study.^[^ ^28^ ^]^ Indeed, if we determine k for C_12_E_10_ with PEG400 only, we obtain k= 0.179 ± 0.010 mol^−1^ L, which is practically the same value as for C_12_E_8_ and DDM. We conclude that for the present set of detergents, the polymer constant is not significantly influenced by the head group, subject to a repetition of the experiments with the $C_{a}E_{b}$ detergents to improve the statistics.

### 2.3 Interpretation of ANS Fluorescence Spectra

**Figure 8:**
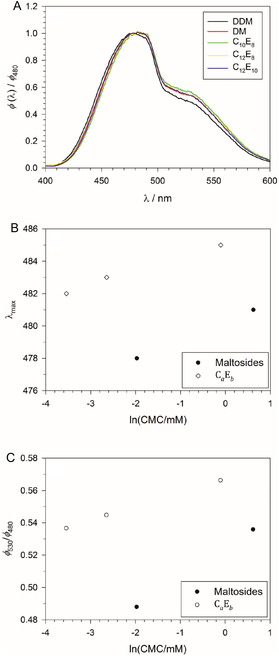
A) Fluorescence spectra in the wavelength range *λ* = 400–600 nm (excitation wavelength: 354 nm) normalized to the fluorescence intensity at 480 nm for the indicated detergents in 100 mM PIPES (pH 7.0), 5 mM CaCl_2_ at 19 °C at the highest detergent concentration investigated, where the fluorescence intensity as a function of detergent concentration levels off as in Figure 2B. The detergent concentrations are (in mM): DDM: 10; DM: 12; C_10_E_8_: 12; C_12_E_8_: 2; C_12_E_10_: 6. B) Correlation of the peak maximum around 480 nm (estimated from the region, where $\phi (\lambda )/\phi_{480}>0.99$) and the logarithm of the CMC in mM. C) Correlation of the peak ratio $\phi_{530}/\phi_{480}$ and the logarithm of the CMC in mM. Figure made with SigmaPlot 13 (2014 Systat Software Inc.).

To better understand the fluorescence spectra, we compared the spectra obtained for the different detergents at the respective highest detergent concentration (at zero PEG concentration), which should correspond mainly to micelle‐bound ANS (**Figure** **8**A). For better comparison, the spectra were normalized to the fluorescence intensity at 480 nm by computing the ratio $\phi (\lambda )/\phi_{480}$. It can be seen that the spectra pertaining to the various detergents differ slightly with respect to the major peak position and the peak ratio $\phi_{530}/\phi_{480}$.

To quantify these differences and gain an interpretation, we plotted the wavelength of the major emission maximum (estimated from the region where $\phi (\lambda )/\phi_{480}>0.99$) and the peak ratio $\phi_{530}/\phi_{480}$ as a function of ln (CMC/mM), where the latter value is taken as a measure of the hydrophilic‐lipophilic balance (HLB) of the detergents (Figure 8B,C; see Table 1 for CMC values). The idea behind these plots needs further explanation: The concept of the HLB is reviewed in Section ST2, Supporting Information (see also Figure S11–S18, Supporting Information, as well as Table S2 and S3, Supporting Information). It was originally introduced for $C_{a}E_{b}$ detergents as a measure of the effectiveness of the surfactants as emulsifiers^[^ ^88^ ^]^ and later also related to other surfactant properties.^[^ ^89^, ^93^ ^–^ ^94^ ^]^ As the name implies, the HLB may be considered as a measure of how hydrophilic or lipophilic an amphiphilic molecule is. Since the tendency to form micelles is higher for a more lipophilic (or equivalently hydrophobic) molecule, one would expect a correlation with the driving force for micelle formation, which is proportional to ln (CMC/mM). Indeed, such correlations have been found,^[^ ^90^, ^91^ ^–^ ^92^ ^]^ but they are not valid in general for all types of detergents, and not even within the class of $C_{a}E_{b}$ detergents (Figure S15, Supporting Information). However, the correlation is perfect for a homologous series of *n*‐alkyl‐ß‐D‐maltosides (Figure S16A, Supporting Information) and octa(ethylene glycol) monoalkylethers (Figure S16B, Supporting Information). We thus take the logarithm of the CMC as a rough measure of the HLB. The correlation between the fluorescence spectra of micelle‐bound ANS and ln (CMC/mM) may then be interpreted as resulting from a change of the overall hydrophobicity of the micelle interior, which can be expected to be larger for larger alkyl chains and hence for smaller CMCs and HLBs. Since the number of data points is rather small (five detergents), we cannot further pursue this type of analysis here. We merely draw the tentative conclusion that the differences in the fluorescence spectra reflect differences in micellar properties.

**Figure 9:**
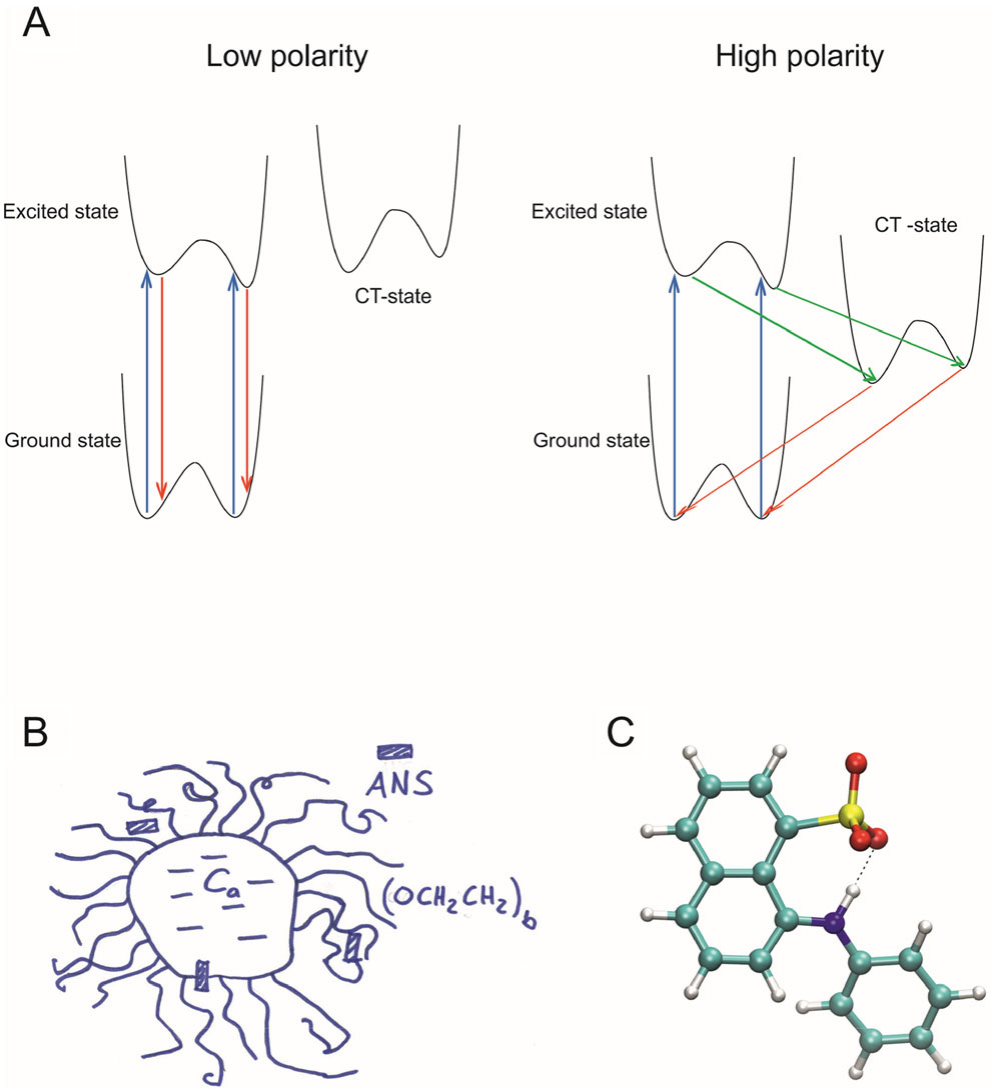
A) Illustration of the two‐conformer model of ANS fluorescence. In this model, ANS has two stable conformations in the electronic ground state, the first excited state, and an intramolecular charge–transfer (CT) state. The relative abundance of the two conformers depends on both the electronic state and the molecular environment of ANS. The blue, red, and green arrows indicate, respectively, absorption, fluorescence, and CT. In both conformations, the excited state is assumed to be fluorescent, whereas the CT state is assumed to be non‐ or weakly fluorescent. B) Sketch of the two‐state model for the solubilization of ANS in $C_{a}E_{b}$ micelles (inspired by Mukerjee^[^ ^13^ ^]^). ANS is either located in the head group region, where it finds conditions similar to PEG, or at the interface between the head group region and the hydrophobic core of the micelle. C) Molecular structure of ANS in one of its likely conformations obtained after energy minimization with density functional theory (DFT) at the PBE0/6‐311G(d,p) level using Q‐Chem.^[^ ^133^ ^]^ The black dashed line indicates a hydrogen‐bond–like interaction between the amine group and the sulfonate moiety. Figure made with VMD.^[^ ^134^ ^]^

It is well known that the fluorescence of molecules like ANS strongly depends on the solvent, which is the reason for its widespread application as a probe in the first place.^[^ ^95^ ^]^ Correlations were found between the fluorescence intensity and peak maximum and the polarity of the solvent. Taking dioxane‐water mixtures as solvent, for example, the emission maximum is shifted from 471 to 530 nm upon increasing the water content from 0.1 to 93.9%(v/v), while the fluorescence quantum yield drops from 52 to 0.3%.^[^ ^96^ ^]^ Under these conditions, one usually observes only one emission peak. This behavior is explained with the existence of a highly fluorescent excited state and a weakly or nonfluorescent charge–transfer (CT) state (i. e. resulting from intramolecular electron transfer), which are in equilibrium. In solvents of low polarity, the excited state is favored and fluorescence originating from this state has a high quantum yield (**Figure** **9**A). Increasing the solvent polarity stabilizes the CT state (due to reaction of the solvent to the large permanent electric dipole of the CT state), so that fluorescence originates predominantly from this state and is thus quenched.^[^ ^97^ ^]^

Only a few spectra reported in the literature of ANS bound to proteins or biological membranes^[^ ^81^ ^–^ ^84^ ^]^ show a more complicated structure with two emission peaks reminiscent of our findings. Outstanding in this respect is the work by Penzer,^[^ ^98^ ^]^ who provided a basic characterization of ANS by NMR spectroscopy and studied, *inter alia* the influence of MgCl_2_ on ANS fluorescence in aqueous solution. Under certain conditions, he observed two emission peaks similar to ours. Using fluorescence excitation spectroscopy, he could show that the two emission peaks pertain to different absorption spectra indicative of an equilibrium between two conformations of ANS. Penzer suggested that in one conformation, the amine group of the anilino substituent forms an intramolecular hydrogen bond to the sulfonate group (which is better described as a hydrogen‐bond–like interaction because of its distorted geometry, see Figure 9C), whereas no such hydrogen bond is formed in the second conformation featuring the phenyl ring in a perpendicular orientation with respect to the naphthalene ring system (not shown). Later studies showed that crystallized ANS occurs in two conformations, which both have an intramolecular hydrogen bond between NH and ${\mathrm{SO}}_{3}^{-}$, but differ in the orientation of the phenyl ring with respect to the naphthalene moiety.^[^ ^99^, ^100^ ^–^ ^101^ ^]^ The two *in crystallo* geometries can be used as proxies for two conformations that ANS might adopt in complex fluid environments. What is appealing about these two structures is that they may well differ in their fluorescence spectra, but at the same time allow both for an intramolecular electron transfer to occur.

To interpret our fluorescence spectra, we don´t have to give geometric details of the ANS conformations. We merely use the spectroscopic and crystallographic information from the literature to construct a minimal model to explain the data. What we have to explain is an observation that has—to our knowledge—not yet been described so explicitly: There are two emission peaks occurring in different proportions that both gain in intensity in less polar environments. This behavior is apparent from micelle‐bound ANS (Figure 2A) as well as from PEG‐bound ANS (Figure 3A,B). In our model, which we refer to as the two‐conformer model (Figure 9A), ANS occurs in two conformations, each with three relevant electronic states: 1) the electronic ground state, 2) the first excited state that is populated by absorption (blue arrows) and gives rise to a fluorescence with a high quantum yield, and 3) a CT state that is populated from the first excited state by intramolecular electron transfer (green arrows) and that has, by virtue of its nature, only a very small fluorescence quantum yield. In each of the three electronic states, the two conformers may occur in different proportions as indicated qualitatively by the various two‐well potential energy surfaces in Figure 9A. In an environment with a low polarity, either CT state is rarely populated, so that fluorescence occurs mainly from the excited state conformers with a high quantum yield (red arrows in the left part of Figure 9A). However, in an environment with a high polarity, both CT state conformers are populated, and fluorescence occurs mainly from these CT states with a low quantum yield (red arrows in the right part of Figure 9A).

For completeness, we mention that fluorescence quenching by intramolecular electron transfer is not the only possibility, although we think that it is the most likely one. Other possible mechanisms are the formation of triplet states by intersystem crossing, enhanced rates for internal conversion (radiationless transition into the ground state) or—as suggested by Penzer^[^ ^98^ ^]^—formation of an N…H—O hydrogen bond between ANS and water that gives rise to a new vibrational degree of freedom for internal conversion. However, as discussed by Kosower and Kanety,^[^ ^96^ ^]^ evidence for a role of intersystem crossing is weak.

The second major finding that we have to explain is the apparent difference in fluorescence peak position and shape between micelle‐bound ANS (Figure 8A) and PEG‐bound ANS (Figure 3A,B). In both conformations of ANS, the transition energies for absorption and emission, as well as their transition probabilities, can be expected to be sensitive to environmental factors such as polarity, dielectric constant, and specific molecular interactions. Due to the latter factor, the abundance of the two conformations may also change. When ANS binds to PEG, both emission peaks are slightly blueshifted. This shift can be ascribed to the environmental factors. The change in relative intensity is likely due to an energetic upshift of the CT states relative to the excited states upon binding to PEG, which may have a different extent in the two conformations and will give rise to an enhanced fluorescence as discussed above.

The location and distribution of solubilizates in micelles is a problem that has been studied for a long time.^[^ ^13^ ^]^ Since the micelle is an inhomogeneous medium and its interior is fluid‐like, there will be several possible binding sites, between which the solubilizate can exchange. Basically, one can distinguish three types of binding sites: 1) the hydrophobic micelle interior, 2) the more hydrophilic head group region, and 3) the interface between the former two. A solubilizate will be distributed between these three regions depending on its molecular properties. ANS is by itself an amphiphilic molecule with the naphthalene moiety and eventually also the phenyl ring defining the hydrophobic side and the sulfonate group the hydrophilic part (Figure 9C). Therefore, ANS can be expected to prefer the interface region. However, since we know from our own data that ANS binds to PEG, it will very likely also bind to the head group region, at least in the case of $C_{a}E_{b}$ detergents. Because of its net charge, it is very unlikely that ANS binds to the micelle interior. These considerations give rise to the two‐state model depicted in Figure 9B, which was developed in the 1970ies, albeit based on a different line of argumentation (related to the spectral properties of aromatic solubilizates and solvent polarity measures^[^ ^13^ ^,^ ^102^ ^,^ ^103^ ^]^).

In our two‐state model, ANS binds either to the head group or the interface region (Figure 9B), and it can exchange between the two. Please, do not confuse the two‐state model with the two‐conformer model. We do not mean that ANS is in one preferred conformation when it binds to one region of the micelle and in the other conformation when it binds to the other region. Rather, we imply that ANS occurs in two conformations in either region. Eventually, the relative abundance of the two conformations varies between the binding regions. So, the two conformational states occur in both binding states. The different molecular environments in the two binding states then give rise to changes in the fluorescence peak positions and intensities.

The two‐state model suggests that the fluorescence spectra shown in Figure 8A represent mixtures of two emitting species pertaining to the different binding states, where each emitting species features two main fluorescence peaks. However, whereas the two peaks originating from the ANS conformers can be distinguished, the two forms of each peak originating from the two binding states are strongly overlapping and cannot be resolved. In the case of $C_{a}E_{b}$ detergents, we can assume that the fluorescence spectrum of ANS bound to the OEG head groups is similar to that of ANS bound to PEG with a peak maximum at 490 nm and a relatively high $\phi_{530}/\phi_{490}$ ratio in the order of 0.75 (cf. Figure 3A,B). The fluorescence spectrum of micelle‐bound ANS can then be explained with the presence of a second emitting species pertaining to ANS in the interface region with a peak maximum closer to 480 nm and a relatively low $\phi_{530}/\phi_{480}$ ratio in the order of 0.5. The second species would also explain the relatively large width and the shape of the major emission peak in the spectra shown in Figure 8A compared to those in Figure 3A,B including the fact that the second emission peak around 530 nm is less well resolved.

The two‐state model also sheds light on the variation of the fluorescence spectrum of micelle‐bound ANS with the detergent. For the $C_{a}E_{b}$ detergents, both $\lambda_{\max}$ and $\phi_{530}/\phi_{480}$ are correlated as can be seen from their dependance on ln (CMC/mM) (Figure 8B,C). The data suggest that the abundance of ANS bound to the interface region between micellar core and head group area is decreased in the order C_12_E_10_, C_12_E_8_, C_10_E_8_ as indicated by an increase of the two parameters. (Note that both the CMC and ln (CMC/mM) increase in the same order.) This redistribution of ANS molecules can principally be rationalized on the basis of differences in the micelle structures. In this respect, it is not the relative length of the OEG head and the alkyl tail that matters, but rather the relative frequency of ANS binding sites in the two regions of the two‐state model. This relative frequency may be roughly quantified by the volume of the head group region and the average surface area of the micellar core. Both can be expected to depend on the aggregation number.

In the case of the alkyl maltosides, we have no independent information about the fluorescence spectrum of ANS bound to the maltose head groups. However, based on the overall similarity of the spectra (Figure 8A), we can hypothesize that it is not much different from the spectrum of PEG‐bound ANS, and shifting ANS to the interface region of the micelle would likewise blueshift the major emission peak and enhance it relative to the 530 nm peak. Then, the lower values of both $\lambda_{\max}$ and $\phi_{530}/\phi_{480}$ (Figure 8B,C) could be interpreted such that the interface region of the micelles is preferred over the head group area by ANS in the maltosides compared to $C_{a}E_{b}$ and also in DDM compared to DM. This could be explained by the smaller size of maltose compared to the OEG head groups on one hand and by the larger aggregation number of DDM compared to DM on the other hand.

Clearly, the presented interpretation of the fluorescence spectra is largely hypothetical and requires further investigations to be substantiated. Nonetheless, the two‐conformer model in conjunction with the two‐state binding model provides a rationale for understanding the experimental data and a suitable starting point for future work.

### 2.4 Further Analysis of Titration Curves

**Figure 10:**
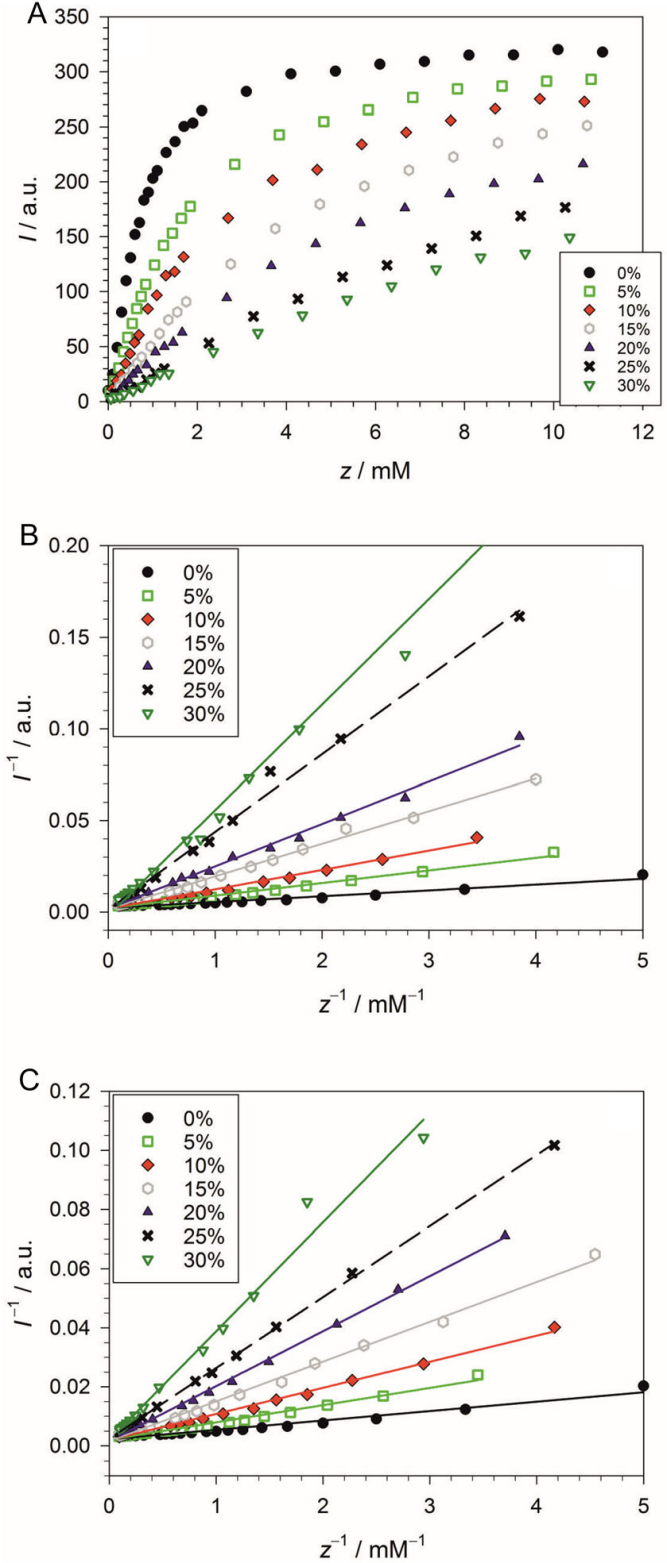
A) Dependance of $I=\phi_{480}-c_{0}$ on $z=[C_{10}E_{8}]-\mathrm{CMC}$ for C_10_E_8_ as computed from the titration curves in Figure 4A for different concentrations of PEG4000 (in %(w/v), see legend) using the CMC values listed in Table 1 and the $c_{0}$ values listed in Table S1 (Supporting Information). B) Double‐reciprocal plot of I(z) data points far above the CMC for C_10_E_8_ and for different concentrations of PEG4000 (in %(w/v), see legend) C) Same as in (B), but for PEG400. Plots made with SigmaPlot 13 (2014 Systat Software Inc.).

With the simplifying assumption that at most one ANS molecule binds to a micelle, the binding constant K is defined by Equation (10),

(10) $$K=\frac{\left[\mathrm{AM}\right]}{\left[A\right]\left[M\right]}$$

where [AM], [A], and [M] are, respectively, the molar concentrations of micelles with one bound ANS molecule, free ANS molecules (i. e., not bound to micelles), and micelles without an ANS molecule attached. Note that K is not the distribution coefficient, which would be defined by $K_{\text{dist}}=[\mathrm{AM}]/[A]$. Motivated by the work of Abuin et al.,^[^ ^67^ ^]^ we derive in Section ST3, Supporting Information, the relationship between K and the fit parameter $b_{1}$ occurring in Equation (7) that represents the initial slope of the titration curves above the CMC (see above). To obtain usable equations, we have to make the assumption that above the CMC, the concentration of detergent in micelles, z, is given by Equation (11),

(11) z=m[M]≈x−CMC

where x is the total detergent concentration and m the aggregation number. This approximation is widely used and corresponds to the usual assumption that above the CMC, the concentration of detergent monomers y=x−z is equal to the CMC. However, even within the framework of a mass action model, the approximation is valid only in the limit of large *m* as discussed in detail by Bothe et al.^[^ ^19^ ^]^ In this limit, the titration curves have a sharp breaking point, and above the CMC, the shape of the curves can be described by the function given in Equation (12),

(12) $$f=\frac{zK}{m+zK}$$

which represents the fraction of ANS molecules bound to micelles. Please, compare the model functions shown in Figure S19–S22, Supporting Information, with the experimental curves shown in Figure 2B and 4A. If we define the quantity $I=\phi -c_{0}$, which means that we subtract from the fluorescence intensity the constant fluorescence level below the CMC, we obtain I=Δϕf, where $\text{Δ}\phi =\phi_{\infty }-c_{0}$ is the difference between the fluorescence intensity at infinite detergent concentration (i.e. the hypothetical limit approached by the titration curve at very high detergent concentrations) and the initial intensity. The I(z) curves look like the examples shown for C_10_E_8_ in **Figure** **10**A.

Describing the I(z) curve with the help of the function f in Equation (12) implies that its initial slope at z=0 equals $b_{1}$. A closer look at the experimental data reveals that this is actually not the case, because the breaking point in the titration curves is not sharp. A combination of Equation (12) with the theory developed by Bothe et al.^[^ ^19^ ^]^ would probably yield a more realistic description of the titration curves but would inevitably lead to a more complex mathematics (see Equation (S83) and (S84), Supporting Information,( for an idea of these complications). Therefore, we stick to the simpler model. In this approximation, it holds that

(13) $$K=\frac{mb_{1}}{\text{Δ}\phi }$$

as shown in Section ST3, Supporting Information. This formula differs from the result obtained by Abuin et al.^[^ ^67^ ^]^ by the occurrence of the aggregation number *m*. Thus, to compute K from $b_{1}$, we need m and Δϕ. A problem is that m is not known for all detergents, and the known m values are afflicted with some uncertainty (cp. Table S2 and S3, Supporting Information).

Another problem is that we have to determine Δϕ. With this goal in mind, we titrated to rather high detergent concentrations to learn as much as possible about the asymptotic behavior of the titration curves, but clearly, they do not reach this limit even at zero PEG concentration. A solution to this problem was suggested by Abuin et al.^[^ ^67^ ^]^: The values of Δϕ can be obtained from data points far above the CMC (z=0) by extrapolation to infinite detergent concentration from double‐reciprocal plots (Figure 10B,C). A plot of $I^{-1}$ versus $z^{-1}$ has intercept $\text{Δ}\phi^{-1}$ and slope

(14) $$s=\frac{m}{K\text{Δ}\phi }$$

(Section ST3, Supporting Information). The results of linear regressions of such plots are compiled in Table S4 (Supporting Information). The plots are shown in Figure 10B,C for C_10_E_8_ and in Figure S23 (Supporting Information) for the other detergents. We learn two lessons from the analysis of the double‐reciprocal plots: 1) There is no evidence for a systematic change of the intercept $\text{Δ}\phi^{-1}$ with the PEG concentration (see the example of C_10_E_8_ shown in Figure S24A,B, Supporting Information), suggesting that it is not affected by the polymer within error margins. In contrast, the slope s is systematically increasing with PEG concentration (Figure S24C, Supporting Information), mirroring the behavior of $b_{1}$, which is correspondingly decreasing (Figure S8, Supporting Information). 2) The determination of $\text{Δ}\phi^{-1}$ from linear regressions of the double‐reciprocal plots is rather insecure. A comparison of the Δϕ values for the various detergents obtained from an average of the data pertaining to ‘good fits’ (i. e. $R^{2}\geq 0.9900$, $\text{Δ}\phi^{-1}>0$) with those estimated from the original titration curves for zero PEG concentration shows a good match only for C_12_E_8_ and C_12_E_10_ (Table S5, Supporting Information). In all other cases, Δϕ is severely overestimated. As a consequence, we computed K on the basis of $b_{1}$ according to Equation (13) —rather than s according to Equation (14) —and used the Δϕ values estimated from the original titration curves assuming that PEG does not affect Δϕ.

Values for K/m and K were obtained for all detergents (Table S5, Supporting Information). For the computation of K, we assumed m=70 for detergents with a=10 and m=120 for a=12, taking into account the uncertainties in the aggregation numbers. Note that this assumption is purely hypothetical for C_12_E_10_, since we have no experimental value for m at our disposal in this case. Accordingly, the binding constants have to be taken as tentative. Two features are of particular note (Figure S25 and S26, Supporting Information): 1) The binding constants decrease nonlinearly with the concentration of OE units of the added PEG and 2) at a given concentration $c_{\mathrm{OE}}$, the effect of PEG4000 is larger than that of PG400. The effect of PEG2000 is similar to that of PEG4000. We conclude that the added PEG competes with the micelles for binding of ANS and, in this way, flattens the titration curves. The shape of the titration curves above the CMC can be understood in terms of the fraction f of ANS bound to micelles, which in turn is determined by K and m according to Equation (12).

It is also noteworthy that K correlates negatively with the logarithm of the CMC (at zero PEG concentration), indicating that the binding of ANS to the micelles is stronger if the driving force for micelle formation is higher (Figure S27, Supporting Information). This effect could be related to the properties of the ANS binding sites in the micelles, which according to the spectroscopic results discussed above (see Figure 8) also correlate with ln (CMC/mM).

### 2.5 Interpretation of the CMC Shift and Implications for Other PEG‐Related Phenomena

As discussed in considerable detail previously^[^ ^19^ ^,^ ^79^ ^]^ (see also the work by Nagarajan and Ruckenstein^[^ ^104^ ^]^ as well as Blankschtein and coworkers^[^ ^105^, ^106^ ^–^ ^107^ ^]^), the micellization free energy can be decomposed into varies contributions according to Equation (15),

(15) $$g_{\mathrm{mic}}=g_{\mathrm{tr}}+g_{\mathrm{int}}+g_{\text{pack}}+g_{\mathrm{st}}$$

where $g_{\mathrm{tr}}$, $g_{\mathrm{int}}$, $g_{\text{pack}}$, and $g_{\mathrm{st}}$ describe, respectively, the transfer (tr) of the alkyl tail from the aqueous medium into the micellar core, the formation of an interface (int) between the micellar core and the aqueous medium partially shielded by the head groups, the packing (pack) of alkyl tails in the micelle interior, and the steric (st) repulsion of head groups in the micelle. Based on this decomposition, we develop a model that is as simple as possible and—in the spirit of the quotation from the beginning of the Introduction—can be used to basically rationalize the PEG‐induced CMC shift without necessarily capturing all physical details, about which we do not have enough information. To this end, we make the following simplifying conjectures; 1) PEG does not interact directly with the micelles. 2) The CMC shift is determined by the alkyl tail, but not the head group. The first conjecture is supported by the observation that Δϕ and the fluorescence properties of micelle‐bound ANS—as far as we can see from the measured spectra—are not significantly altered by PEG. The second conjecture is suggested by the dependance of the polymer constant k on the alkyl chain length and its independence of the head group—subject to a verification of the case C_12_E_10_/PEG4000.

Since $g_{\mathrm{int}}$, $g_{\text{pack}}$, and $g_{\mathrm{st}}$ all depend only on the properties of the micelles, they should not be significantly affected by PEG. In contrast, $g_{\mathrm{tr}}$ depends on the alkyl chain length and thus may be suspected to be relevant for the PEG effect. Then, denoting $g_{\mathrm{tr}}$ in the absence of PEG by $g_{\mathrm{tr}}^{0}$, we can write by taking together (Equation 2) and (3):

(16) $$kc_{\mathrm{OE}}=g_{\mathrm{mic}}-g_{\mathrm{mic}}^{0}\approx g_{\mathrm{tr}}-g_{\mathrm{tr}}^{0}$$

Note that this is supposed to be an approximation. We do not mean that micelle–PEG interactions or the detergent head groups are totally irrelevant. We merely consider the change of $g_{\mathrm{tr}}$ to be the dominant factor for the CMC shift in our model.

The $g_{\mathrm{tr}}$ term is part of the PCP difference between detergent in micelles and detergent monomers.^[^ ^79^ ^]^ Following Ben‐Naim,^[^ ^77^ ^]^ we can interpret it as the work necessary to transfer the alkyl tail from a fixed position in the aqueous medium into the micelle. The notion of a ‘fixed position’ simply means that we do not consider the translational motion of the alkyl tail, but only its interaction with the surrounding medium. Based on our conjectures, PEG does not affect the surrounding of the alkyl tail within the micelle, but only the interaction with the aqueous medium it has in the detergent monomer. In Ben‐Naim´s notation, R(1|A+B+C+…) would be the work to bring molecule 1 into the environment consisting of molecules A, B, C, and so on. Thus, if 1 denotes the alkyl tail of a detergent monomer as well as W, B, and P, respectively, water, buffer, and PEG, we can write

(17) $$g_{\mathrm{tr}}-g_{\mathrm{tr}}^{0}=-\beta \left[R\left(1|W+B+P\right)-R\left(1|W+B\right)\right]$$

where $\beta =1/(k_{B}T)$ is the inverse thermal energy. In Section ST1, Supporting Information, we show how the rather complicated statistical integrals behind the work term can be boiled down to a simple form (cf. Equation (S57), Supporting Information), so that

(18) $$R\left(1|W+B+P\right)-R\left(1|W+B\right)=c_{2}J_{12}$$

Here, 2 stands for the OE unit, while $J_{12}$ is a parameter describing the interaction of the alkyl tail with the OE units containing all the complicated averages over molecular configurations in the solution. The punchline is that we can—combining (Equation 16) to (18) —interpret the polymer constant according to

(19) $$k=-\beta J_{12}$$

as such an interaction parameter divided by the thermal energy. Since k is positive, the minus sign in Equation (19) indicates that $J_{12}$ has to be negative, with the physical meaning that PEG stabilizes the alkyl tail in the aqueous solution (i. e. the free energy per monomeric detergent molecule as represented by the standard chemical potential is decreased; cf. Section ST1, Supporting Information). This interpretation is in line with the fact that a stabilization of the detergent monomer will increase the CMC.

What does it mean that the polymer constant is independent of the actual covalent connectivity of the PEG chains, so that only the overall concentration of OE units matters? In our previous work,^[^ ^28^ ^]^ we found that the (mass) density of PEG solutions in our buffer system increases linearly with $c_{\mathrm{OE}}$ in a way that is independent of the polymer length. This finding is in agreement with literature data for PEG in pure water^[^ ^108^, ^109^, ^110^ ^–^ ^111^ ^]^ and is related to the observation that the total molarity of the PEG solutions can be described by Equation (20),

(20) $$c_{\mathrm{tot}}=c_{\mathrm{ref}}-qc_{\mathrm{OE}}$$

where $c_{\mathrm{ref}}$ is the reference molarity of PEG‐free buffer (or water, in which case $c_{\mathrm{ref}}=55.56M$) and q=2.073.^[^ ^28^ ^]^ Equation (20) can be rationalized by assuming that each OE unit displaces about two water molecules in the solution (q≈2). Since the OE unit is heavier than the two water molecules, the density increases.

The notion of two water molecules being displaced by one OE unit is in accordance with structural models of aqueous PEG that were developed to explain the unusually high solubility of the polymer in water.^[^ ^112^ ^,^ ^113^ ^]^ It was noted by Blandamer et al.^[^ ^114^ ^]^ that the water molecules surrounding PEG in aqueous solution can form hydrogen bonding networks similar to that of bulk water due to suitable distances between oxygen atoms, which distinguishes PEG from other polymers such as poly(methylene oxide) or poly(propylene oxide). A relatively recent study by Wada et al.^[^ ^112^ ^]^ investigated the conformational distribution of the model compound dimethoxyethane (DME) in water by means of Raman spectroscopy and thermodynamic analyses. It was shown that the trans‐gauche‐trans (tgt) conformation of DME enjoys by far the largest free energy gain when being transferred from pure liquid DME into a diluted aqueous solution. This result provides clear evidence for the mechanism of the high solubility of PEG in agreement with the earlier structural models and spectroscopic results obtained on PEG solutions.^[^ ^115^, ^116^, ^117^ ^–^ ^118^ ^]^ Molecular dynamics (MD) simulations confirm that due to its conformational preferences in an aqueous environment, PEG chains form rather elongated structures in water with a high degree of hydration of the OE units rather than collapsed globular structures.^[^ ^119^, ^120^ ^–^ ^121^ ^]^ A particularly compelling feature of aqueous PEG is the extreme ratio on the order of 0.3 of its radius of gyration and the root mean square end‐to‐end distance, accompanied by a significant shape anisotropy.^[^ ^122^ ^]^

What is the relevance of these findings for the PEG effect on micelle formation? A rather extended PEG chain implies that the hydrophobic part of the OE unit, that is, the C_2_H_4_ group, is well accessible to other molecules and it is well hydrated. It is then conceivable that a detergent monomer tends to come into contact with the C_2_H_4_ group via its alkyl tail by virtue of the hydrophobic effect and in this way is stabilized in the aqueous solution. If the C_2_H_4_ groups are freely accessible, the probability for such a detergent‐PEG contact is proportional to the concentration of OE units and does not depend on the chain length of PEG. Furthermore, the probability for the contact is higher the longer the alkyl tail of the detergent, which explains why k increases with a.

Our results argue against a dominating role of micelle–PEG interactions for the CMC shift. However, it can certainly not be excluded that micelles come into contact with PEG chains, particularly at higher PEG concentrations. If such contacts stabilize the micelles, this effect cannot be resolved in our data, and it would still be different from the behavior of ionic detergents, since we do not observe a decrease of the CMC. Our improved analysis of the titration curves allows identifying the breaking point indicating the CMC even at higher PEG concentrations, where the curves are significantly flattened. Micelles are still formed as indicated by the ANS fluorescence spectra, and the definition of the CMC by means of the breaking point remains feasible. We think that the CAC is not a pertinent concept here, because the promotion of micelle formation by the polymer is not the main effect.

A widely studied nonionic detergent is Triton X‐100, which is also employed in membrane protein research.^[^ ^123^ ^,^ ^124^ ^]^ Although it has a 4‐(1,1,3,3‐tetramethylbutyl)‐phenyl group instead of an alkyl tail, results concerning its interaction with PEG are of interest. Ge et al.^[^ ^125^ ^]^ studied the properties of TX‐100 in 5% (w/v) PEG, where the size of the PEG varied from 400 to 20 000. They found a slight increase of the CMC, which appears to depend on the PEG size, but no change of the aggregation number. Evidence was reported based on fluorescence energy transfer (FRET), NMR spectroscopy, and dynamic light scattering (DLS) that PEG interacts with micelles. This interaction involves a penetration of PEG into the head group area of TX‐100 (which also has an OEG head group), but the PEG chains do not thread through the micelle core. The insensitivity of the aggregation number to 5% PEG4000 was confirmed later by Patel et al.^[^ ^126^ ^]^ using small angle neutron scattering, while a change of the hydrodynamic radius of the micelles determined by DLS was interpreted in terms of a wrapping of PEG chains around the micelles. Aktar et al.^[^ ^127^ ^]^ compared the interaction of PEG400 with an ionic (TTAB, tetradecyltrimethylammonium bromide) and a nonionic (TX‐100) detergent. Their MD simulations demonstrated that PEG400 chains enter into TTAB micelles but remain on the micellar surface of TX‐100, thus highlighting the distinctly different behavior of ionic and nonionic detergents.

An interesting finding by Ge et al.^[^ ^125^ ^]^ concerns their FRET experiments. In this case, the phenyl moiety of TX‐100 is the donor, and the added probe molecule pyrene is the acceptor. Based on their analysis, the average distance between donor and acceptor increases by about 10 Å due to addition of PEG. The distance change is independent of the PEG size. Ge et al. interpret this result by an attachment of PEG chains to or a penetration into the head group region of the micelles, causing a redistribution of pyrene within the micelle and an effective enlargement of the micellar aggregates. Note that this does not require a change of the aggregation number. In our experiments, attachment of PEG chains to the micelles could cause a redistribution of ANS between the two binding regions according to the two‐state model. We would then expect an increase of the $\phi_{530}/\phi_{490}$ ratio. However, as far as the flattened titration curves allow for such an analysis, we do not observe an effect of PEG on this fluorescence ratio. Thus, either there is no appreciable attachment of PEG chains to the micelles or the PEG–micelle interaction does not cause a redistribution of ANS. We imagine that the interaction of PEG chains with micelles is similar to what Aktar et al.^[^ ^127^ ^]^ observe in their MD simulations of the TX‐100/PEG400 system: The PEG chains are loosely attached so that they do not change the free energy of the micelles to the extent that it can be resolved in our CMC measurements.

What are the consequences for other PEG‐related phenomena? We will focus the discussion on two problems: 1) the PEG effect on the CSC and 2) the extraction of detergent from protein crystals.

1) As discussed previously,^[^ ^15^ ^,^ ^28^ ^]^ the standard Gibbs free energy for the solubilization of a membrane protein, $\text{Δ}G_{\mathrm{sol}}^{0}$, has two parts according to Equation (21).

(21) $$\text{Δ}G_{\mathrm{sol}}^{0}=\text{Δ}G_{\text{diss}}^{0}+\text{Δ}G_{\text{belt}}^{0}$$

Here, $\text{Δ}G_{\text{diss}}^{0}$ refers to the dissociation of protein aggregates, while $\text{Δ}G_{\text{belt}}^{0}$ is the driving force for the assembly of the detergent belt surrounding the hydrophobic protein surface. With the conjectures discussed above concerning the PEG–detergent interaction, we can conclude that PEG will shift $\text{Δ}G_{\text{belt}}^{0}$ in a way similar to $g_{\mathrm{mic}}$ (cf. our previous discussion^[^ ^28^ ^]^). Thus, the solubility of the membrane protein will be affected such that more detergent is required to solubilize it in the presence of PEG than in its absence (depending on the buffer conditions). However, $\text{Δ}G_{\text{diss}}^{0}$ also has to be taken into account for a complete understanding of the CSC. The mechanism underlying this free energy contribution is likely the same for membrane proteins as for soluble proteins, and it is elusive. An interesting related problem is the interaction between detergent belts and micelles that are likely influenced by PEG in a similar way.^[^ ^24^ ^,^ ^25^ ^,^ ^128^ ^,^ ^129^ ^]^ The range of PEG concentrations that we could investigate was also influenced by these effects, limiting the solubility of micelles in the buffer. A more systematic investigation of these limits as well as their temperature dependance was beyond the scope of the present project, so that we can neither discuss solubility changes of micelles nor PEG effects on the cloud points of the detergents in detail. Possibly, depletion forces play a role here, making the influence of PEG depending on the chain length. A further complication is that the extended conformation of the PEG chain will cause simple models of excluded volume effects, in which PEG is described as a globular structure, to fail. This is also a problem for a theoretical description of the fractional precipitation of proteins by PEG. In conclusion, the present results increase our understanding of the CSC in the context of membrane protein crystallization only partly.

2) There are basically two types of membrane protein crystals:^[^ ^50^ ^,^ ^51^ ^]^ In type II crystals, the protein–detergent complex is crystallized as a whole, including the detergent belt. As a consequence, these crystals are detergent‐rich, which comes at the expense of protein–protein contacts in the crystal and hence crystal stability and quality. In contrast, type I crystals are detergent‐depleted and the membrane proteins are stacked in layers with tight contacts of their hydrophobic surfaces. These crystals are often more stable and of higher quality and occasionally may show an arrangement of the protein units similar to crystalline aggregates formed in the native biomembrane.^[^ ^130^ ^]^ In an examination of the role of the detergent in the crystallization of PSII,^[^ ^72^ ^]^ it was found that postcrystallization treatments to improve the crystal quality resulted in detergent depletion and an unexpected transformation from type II to type I. The treatment involved soaking the crystals in a solution of PEG5000 monomethyl ether (MME) to dehydrate it. Since the procedure worked with C_12_E_8_ but not with DDM, it was suggested that PEG5000MME might have a different effect on the stability of detergent monomers outside the crystals in the case of C_12_E_8_ compared to DDM, which was substantiated by measuring different effects of PEG on the CMC (see SI of ref. [72]). Based on the present results, we have to conclude that the difference between C_12_E_8_ and DDM in this respect is not significant. In fact, the polymer constant of DDM was overestimated in this earlier study (k=0.267), whereas that for C_12_E_8_ was similar to the present data (k=0.178). As a consequence, it can be assumed that PEG, while promoting the extraction of detergent from the crystal, does so in the same way for C_12_E_8_ and DDM. Thus, the different behavior of the two detergents must have another origin. We hypothesize that two aspects are of importance: 1) the free energy for binding of the detergent to the protein surface and 2) the flexibility and size of the head groups influencing the diffusive motion of the detergent in the crystal. In conclusion, the present results brought us a small step closer to an understanding of the detergent extraction mechanism. In view of the importance of this crystal type (e. g., PSII^[^ ^73^ ^–^ ^76^ ^]^), it is certainly worthwhile to further investigate this issue.

## 3 Conclusion

In the area of detergent–polymer interactions, the focus is on ionic detergents, while our knowledge about the influence of polymers on nonionic detergents is incomplete. The latter is not only of fundamental interest but also important for a deeper understanding of the physical chemistry behind the structural and biochemical characterization of membrane proteins. To this end, we tried to quantify the effect of PEG, a water‐soluble polymer often employed as precipitating agent in protein analytics and crystallization, on the self‐assembly (CMC) of nonionic detergents frequently used in applications related to membrane protein research. It is fair to say that the quantification of the CMC shift caused by PEG is challenging despite the relatively simple nature of the fluorescence assay we used. The problems lie in the compromises caused by counteracting factors such as time and resource limits, data statistics, the simplicity and practicability of the experimental methods as well as the usability and comprehensibility of theoretical underpinnings for an interpretation of the data. Within this framework, we performed a straightforward set of titration experiments, refined their analytical processing, and attempted to rationalize the findings with the help of models that—despite being largely simplified—capture the essential aspects and are still firmly rooted in statistical thermodynamics and molecular physics.

We were able to confirm our earlier result^[^ ^28^ ^]^ that the CMC shift of the investigated detergents (cf. Figure 1) caused by PEG is determined by the concentration of OE units and—within the framework of investigated PEG concentrations—independent of the molecular weight of the polymer chain. The molecular explanation for this effect is found in the relatively extended conformation of the PEG chains in an aqueous environment, allowing for an interaction of individual OE units with detergent monomers. Thus, monomer–polymer interactions are relevant for nonionic detergents. However, this type of binding to the polymer certainly also occurs with ionic detergents and should be considered. In this respect, we note that we are working here with relatively high PEG concentrations, 5%–10% (w/v) being typical for crystallization setups of proteins.^[^ ^131^ ^]^ Many studies in the area of detergent–polymer interactions may not reach this level, and their findings might be dominated by other effects.^[^ ^132^ ^]^ Also, the CMC shift caused by PEG is rather subtle with $\ln\;(\mathrm{CMC}/{\mathrm{CMC}}_{0})\leq 1$ for up to 30% (w/v) PEG (cf. Figure 6, where 30% (w/v) roughly corresponds to $c_{\mathrm{OE}}=6.5-6.8M$).

When we first applied the ANS method,^[^ ^27^ ^]^ we did this rather naively by focusing on the fluorescence enhancement at a particular wavelength without paying too much attention to the emission spectra. This is probably the way the method is used by many. Accordingly, we were surprised to find spectra with a rich structure in the present work. A survey of the literature gave us the impression that ANS is not as well understood as one might expect based on its use as fluorescence probe for many decades. Ruling out a pH dependance, which we neither found in initial tests (data not shown) nor expected because of the high acidity of the sulfonate group and the strong binding of the amine proton to it, we used the sparse information from the literature to build a minimal model for an understanding of our spectra. This model asserts that ANS binds to two different types of binding sites in the micelles and occurs in two spectroscopically distinct conformations. Clearly, more work needs to be done to evaluate the model. We think that ANS is still an extremely interesting molecule from the viewpoints of analytical chemistry, quantum chemistry as well as molecular physics. Deepening our understanding of its fluorescence properties will be of use for the fields of detergent–polymer interactions and biophysics.

As regards more practical aspects, the present work provided an interesting piece of information for the field of the structural biology of membrane proteins: PEG stabilizes detergent monomers of DDM and C_12_E_8_ to the same extent. At least, we could not find any statistically reliable difference yet, and earlier findings concerning such a difference^[^ ^72^ ^]^ have to be revised. This finding bears implications for a mechanistic understanding of the transformation from type II to type I crystals. The transformation method is relatively new and particularly suitable for membrane proteins that tend to form crystalline arrangements in the native biomembrane^[^ ^130^ ^]^ and for microcrystals used in time‐resolved X‐ray methods.^[^ ^74^ ^]^ Further studies to learn about the underlying molecular mechanisms and ways to optimize it are certainly of high interest.

## 4 Experimental Section

4.1

4.1.1

##### General Information

DDM and DM were purchased from Glycon (Luckenwalde, Germany), all other detergents, as well as ANS (ammonium salt, HPLC grade), and all other chemicals from Merck/Sigma–Aldrich. All experiments were performed in buffer solution containing 100 mM piperazine‐1,4‐bis‐(2‐ethanesulfonic acid) (PIPES; adjusted to pH 7.0 with NaOH) and 5 mM CaCl_2_, corresponding to the buffer conditions suitable for the crystallization of photosystem II^[^ ^41^ ^]^ also used in previous work.^[^ ^27^ ^]^

##### Fluorescence Measurements

All samples for spectroscopy were prepared from appropriate stock solutions to achieve the desired concentrations for each data point in a titration. Measurements were performed by using Hellma macro cuvettes made of quartz glass suitable for the spectral window from 200 to 2500 nm with a 10 × 10 mm path length and chamber volume of 3.5 mL. The actual sample volume was 2 mL. The concentration of ANS was 10 µM in all samples. The fluorescence (emission) spectra were measured with a Jasco FP‐8300 spectrofluorimeter detected in the range from 400 to 600 nm with excitation at 354 nm and a data interval of 1 nm resulting in 201 data points per spectrum. The excitation and emission bandwidths were 1 and 5 nm, respectively. The light source was a Xe lamp. The detector response was 50 ms with medium sensitivity and a scan speed of 1000 nm/min. No filters, blank corrections, or auto‐gain functions were used. The temperature in the cuvette chamber was monitored, and care was taken to keep it in the range of 19 ± 1 °C.

## Supporting Information

ST1: Thermodynamics of micelle formation, ST2: The hydrophilic‐lipophilic balance (HLB), ST3: Equilibrium constant for the binding of ANS to micelles, Tables S1–S6, Figures S1–S27. The authors have cited additional references within the Supporting Information.^[135‐180]^

## Conflict of Interest

The authors declare no conflict of interest.

## Supporting information

Supplementary Material

## Data Availability

The data that support the findings of this study are available in the supplementary material of this article.

## References

[1] F. M. Menger , Acc. Chem. Res. 1979, 12, 111.

[2] J. W. McBain , Trans. Faraday Soc. 1913, 9, 99.

[3] B. Vincent , Adv. Colloid Interface Sci. 2014, 203, 51. 24345732 10.1016/j.cis.2013.11.012

[4] B. Kronberg , Curr. Opin. Colloid Interface Sci. 2016, 22, 14.

[5] N. F. A. van der Vegt , D. Nayar , J. Phys. Chem. B 2017, 121, 9986. 28921974 10.1021/acs.jpcb.7b06453

[6] D. Chandler , Nature 2005, 437, 640. 16193038 10.1038/nature04162

[7] W. Blokzijl , J. B. F. N. Engberts , Angew. Chem., Int. Ed. 1993, 32, 1545.

[8] Y. Moroi , Micelles: Theoretical and Applied Aspects, Plenum Press, New York, NY 1992.

[9] B. Aveyard , Surfactants: In Solution, at Interfaces and in Colloidal Dispersions, Oxford University Press, Oxford 2019.

[10] V. Wycisk , M. C. Wagner , L. H. Urner , ChemPlusChem 2024, 89, e202300386. 37668309 10.1002/cplu.202300386

[11] D. P. Bockmühl , T. J. Tewes , ChemPlusChem 2025, 90, e202400657. 39514416 10.1002/cplu.202400657 PMC11826111

[12] J. W. McBain , C. S. Salmon , J. Am. Chem. Soc. 1920, 42, 426.

[13] P. Mukerjee , in Solution Chemistry of Surfactants, Vol. 1 (Ed: K. L. Mittal ), Plenum Press, New York, NY 1979, pp. 153–174.

[14] D. R. Pokhrel , M. K. Sah , B. Gautam , H. K. Basak , A. Bhattarai , A. Chatterjee , RSC Adv. 2023, 13, 17685. 37312992 10.1039/d3ra02883f PMC10258811

[15] F. Müh , A. Zouni , Biochim. Biophys. Acta, Biomembr. 2008, 1778, 2298. 10.1016/j.bbamem.2008.05.016 18602888

[16] J. N. Phillips , Trans. Faraday Soc. 1955, 51, 561.

[17] J. M. Corkill , J. F. Goodman , S. P. Harrold , Trans. Faraday Soc. 1964, 60, 202.

[18] W. Al‐Soufi , L. Pineiro , M. Novo , J. Colloid Interface Sci. 2012, 370, 102. 22265231 10.1016/j.jcis.2011.12.037

[19] A. Bothe , A. Zouni , F. Müh , RSC Adv. 2023, 13, 9387. 36968053 10.1039/d2ra07440k PMC10031436

[20] Interactions of Surfactants with Polymers and Proteins (Eds: E. D. Goddard , K. P. Ananthapadmanabhan ), CRC Press, Boca Raton 1993.

[21] K. Holmberg , B. Jönsson , B. Kronberg , B. Lindman , Surfactants and Polymers in Aqueous Solution, Wiley, Chichester 2003.

[22] O. Anthony , R. Zana , Langmuir 1994, 10, 4048.

[23] M. Aoudia , R. Zana , J. Colloid Interface Sci. 1998, 206, 158. 9761639 10.1006/jcis.1998.5627

[24] C. Hitscherich , J. Kaplan , M. Allaman , J. Wiencek , P. J. Loll , Protein Sci. 2000, 9, 1559. 10975577 10.1110/ps.9.8.1559 PMC2144733

[25] J. Blouwolff , S. Fraden , J. Cryst. Growth 2007, 303, 546.

[26] M. G. Santonicola , M. A. Yocum , A. M. Lenhoff , E. W. Kaler , Langmuir 2007, 23, 5358. 17429988 10.1021/la063427d

[27] F. Müh , D. DiFiore , A. Zouni , Phys. Chem. Chem. Phys. 2015, 17, 11678. 25865704 10.1039/c5cp00431d

[28] F. Müh , A. Bothe , A. Zouni , Photosynth. Res. 2024, 162, 273. 38488943 10.1007/s11120-024-01079-5 PMC11615006

[29] K. P. Ananthapadmanabhan , in Interactions of Surfactants with Polymers and Proteins (Eds: E. D. Goddard , K. P. Ananthapadmanabhan ), CRC Press, Boca Raton 1993, pp. 319–365.

[30] D. Otzen , Biochim. Biophys. Acta, Proteins Proteomics 2011, 1814, 562. 10.1016/j.bbapap.2011.03.003 21397738

[31] G. G. Privé , Methods 2007, 41, 388. 17367711 10.1016/j.ymeth.2007.01.007

[32] M. le Maire , P. Champeil , J. V. Moller , Biochim. Biophys. Acta, Biomembr. 2000, 1508, 86. 10.1016/s0304-4157(00)00010-1 11090820

[33] D. Linke , Methods Enzymol. 2009, 463, 603. 19892194 10.1016/S0076-6879(09)63034-2

[34] S. H. Lin , G. Guidotti , Methods Enzymol. 2009, 463, 619. 19892195 10.1016/S0076-6879(09)63035-4

[35] A. Stetsenko , A. Guskov , Crystals 2017, 7, 197.

[36] R. E. Blankenship , Molecular Mechanisms of Photosynthesis, Wiley, Hoboken 2021.

[37] W. A. Cramer , T. Kallas , Cytochrome Complexes: Evolution, Structures, Energy Transduction, and Signaling, Springer, Dordrecht 2016.

[38] T. Rühle , D. Leister , V. Pasch , Plant Cell 2024, 36, 3974. 38484126 10.1093/plcell/koae081 PMC11449085

[39] T. M. Iverson , P. K. Singh , G. Cecchini , J. Biol. Chem. 2023, 299, 104761. 37119852 10.1016/j.jbc.2023.104761 PMC10238741

[40] R. G. Contreras , A. Torres‐Carrillo , C. Flores‐Maldonado , L. Shoshani , A. Ponce , Int. J. Mol. Sci. 2024, 25, 6122. 38892309 10.3390/ijms25116122 PMC11172918

[41] B. Loll , J. Kern , W. Saenger , A. Zouni , J. Biesiadka , Nature 2005, 438, 1040. 16355230 10.1038/nature04224

[42] F. Müh , A. Zouni , Protein Sci. 2020, 29, 1090. 32067287 10.1002/pro.3841 PMC7184784

[43] E. Gulezian , C. Crivello , J. Bednenko , C. Zafra , Y. H. Zhang , P. Colussi , S. Hussain , Trends Pharmacol. Sci. 2021, 42, 657. 34270922 10.1016/j.tips.2021.05.006

[44] E. Sobakinskaya , H. Krobath , T. Renger , F. Müh , Phys. Chem. Chem. Phys. 2021, 23, 25830. 34762087 10.1039/d1cp02702f PMC8612361

[45] E. Sobakinskaya , F. Müh , Phys. Chem. Chem. Phys. 2024, 26, 27176. 39435495 10.1039/d4cp03201b PMC11494458

[46] M. Trebak , J. W. Putney , Physiology 2017, 32, 332. 28615316 10.1152/physiol.00011.2017 PMC5545608

[47] A. Tiffner , L. Maltan , S. Weiss , I. Derler , Int. J. Mol. Sci. 2021, 22, 533. 33430308 10.3390/ijms22020533 PMC7825772

[48] C. S. Vrettou , V. Issaris , S. Kokkoris , G. Poupouzas , C. Keskinidou , N. S. Lotsios , A. Kotanidou , S. E. Orfanos , I. Dimopoulou , A. G. Vassiliou , Life 2024, 14, 1688. 39768394 10.3390/life14121688 PMC11676363

[49] N. Gössweiner‐Mohr , C. Siligan , K. Pluhackova , L. Umlandt , S. Koefler , N. Trajkovska , A. Horner , Small 2022, 18, 2202056. 10.1002/smll.202202056 35802902

[50] H. Michel , Trends Biochem. Sci. 1983, 8, 56.

[51] C. Ostermeier , H. Michel , Curr. Opin. Struct. Biol. 1997, 7, 697. 9345629 10.1016/s0959-440x(97)80080-2

[52] N. Thonghin , V. Kargas , J. Clews , R. C. Ford , Methods 2018, 147, 176. 29702228 10.1016/j.ymeth.2018.04.018

[53] M. Golub , J. Pieper , Molecules 2023, 28, 7414. 37959833 10.3390/molecules28217414 PMC10650700

[54] F. Müh , A. Zouni , Biochim. Biophys. Acta, Bioenerg. 2005, 1708, 219. 10.1016/j.bbabio.2005.03.005 15953478

[55] A. McPherson , J. A. Gavira , Acta Crystallogr., Sect. F 2014, 70, 2. 10.1107/S2053230X13033141 PMC3943105 24419610

[56] A. McPherson , J. Biol. Chem. 1976, 251, 6300. 977570

[57] S. D. Durbin , G. Feher , Annu. Rev. Phys. Chem. 1996, 47, 171. 8983237 10.1146/annurev.physchem.47.1.171

[58] D. H. Atha , K. C. Ingham , J. Biol. Chem. 1981, 256, 2108. 7298647

[59] K. C. Ingham , Methods Enzymol. 1990, 182, 301. 2314243 10.1016/0076-6879(90)82025-w

[60] D. Hekmat , Bioprocess Biosyst. Eng. 2015, 38, 1209. 25700885 10.1007/s00449-015-1374-y

[61] R. dos Santos , A. L. Carvalho , A. C. A. Roque , Biotechnol. Adv. 2017, 35, 41. 27908674 10.1016/j.biotechadv.2016.11.005

[62] R. J. Ellis , A. P. Minton , Biol. Chem. 2006, 387, 485. 16740119 10.1515/BC.2006.064

[63] R. Wälchli , F. Fanizzi , J. Massant , P. Arosio , Eur. J. Pharm. Biopharm. 2020, 151, 53. 32197816 10.1016/j.ejpb.2020.03.011

[64] J. Bloustine , T. Virmani , G. M. Thurston , S. Fraden , Phys. Rev. Lett. 2006, 96, 087803. 16606227 10.1103/PhysRevLett.96.087803

[65] W. Y. Chen , M. Y. Hsu , C. W. Tsai , Y. Chang , R. C. Ruaan , W. H. Kao , E. W. Huang , H. Y. T. C. Chuan , Langmuir 2013, 29, 4259. 23330911 10.1021/la304500w

[66] E. De Vendittis , G. Palumbo , G. Parlato , V. Bocchini , Anal. Biochem. 1981, 115, 278. 7304960 10.1016/0003-2697(81)90006-3

[67] E. B. Abuin , E. A. Lissi , A. Aspée , F. D. Gonzalez , J. M. Varas , J. Colloid Interface Sci. 1997, 186, 332. 9056362 10.1006/jcis.1996.4648

[68] P. Mukerjee , J. Phys. Chem. 1965, 69, 4038.

[69] A. Ray , G. Némethy , J. Am. Chem. Soc. 1971, 93, 6787. 5133091 10.1021/ja00754a014

[70] T. R. Carale , Q. T. Pham , D. Blankschtein , Langmuir 1994, 10, 109.

[71] S. Miyagishi , K. Okada , T. Asakawa , J. Colloid Interface Sci. 2001, 238, 91. 11350141 10.1006/jcis.2001.7503

[72] J. Hellmich , M. Bommer , A. Burkhardt , M. Ibrahim , J. Kern , A. Meents , F. Müh , H. Dobbek , A. Zouni , Structure 2014, 22, 1607. 25438669 10.1016/j.str.2014.09.007

[73] J. Kern , R. Chatterjee , I. D. Young , F. D. Fuller , L. Lassalle , M. Ibrahim , S. Gul , T. Fransson , A. S. Brewster , R. Alonso‐Mori , R. Hussein , M. Zhang , L. Douthit , C. de Lichtenberg , M. H. Cheah , D. Shevela , J. Wersig , I. Seuffert , D. Sokaras , E. Pastor , C. Weninger , T. Kroll , R. G. Sierra , P. Aller , A. Butryn , A. M. Orville , M. N. Liang , A. Batyuk , J. E. Koglin , S. Carbajo , et al., Nature 2018, 563, 421. 30405241 10.1038/s41586-018-0681-2 PMC6485242

[74] J. Kern , F. Müh , A. Zouni , in Metabolism, Structure and Function of Plant Tetrapyrroles: Control Mechanisms of Chlorophyll Biosynthesis and Analysis of Chlorophyll‐Binding Proteins (Ed: B. Grimm ), Elsevier, London 2019, pp. 33–67.

[75] R. Hussein , M. Ibrahim , A. Bhowmick , P. S. Simon , R. Chatterjee , L. Lassalle , M. Doyle , I. Bogacz , I. S. Kim , M. H. Cheah , S. Gul , C. de Lichtenberg , P. Chernev , C. C. Pham , I. D. Young , S. Carbajo , F. D. Fuller , R. Alonso‐Mori , A. Batyuk , K. D. Sutherlin , A. S. Brewster , R. Bolotovsky , D. Mendez , J. M. Holton , N. W. Moriarty , P. D. Adams , U. Bergmann , N. K. Sauter , H. Dobbek , J. Messinger , et al., Nat. Commun. 2021, 12, 6531. 34764256 10.1038/s41467-021-26781-z PMC8585918

[76] A. Bhowmick , R. Hussein , I. Bogacz , P. S. Simon , M. Ibrahim , R. Chatterjee , M. D. Doyle , M. H. Cheah , T. Fransson , P. Chernev , I. S. Kim , H. Makita , M. Dasgupta , C. J. Kaminsky , M. Zhang , J. Gaetcke , S. Haupt , I. I. Nangca , S. M. Keable , A. O. Aydin , K. Tono , S. Owada , L. B. Gee , F. D. Fuller , A. Batyuk , R. Alonso‐Mori , J. M. Holton , D. W. Paley , N. W. Moriarty , F. Mamedov , et al., Nature 2023, 617, 629. 37138085 10.1038/s41586-023-06038-z PMC10191843

[77] A. Ben‐Naim , J. Phys. Chem. 1978, 82, 792.

[78] T. L. Hill , An Introduction to Statistical Thermodynamics, Dover, New York, NY 1960.

[79] F. Müh , Colloids Interfaces 2024, 8, 60.

[80] J. N. Israelachvilli , Intermolecular and Surface Forces, Academic Press, Elsevier, Amsterdam 2011.

[81] J. K. Yadav , V. Prakash , Int. J. Food Prop. 2011, 14, 1182.

[82] T. Gulik‐Krzywicki , E. Shechter , M. Iwatsubo , J. L. Ranck , V. Luzzati , Biochim. Biophys. Acta, Biomembr. 1970, 219, 1. 10.1016/0005-2736(70)90055-6 4319692

[83] A. G. Lee , J. Rogers , D. C. Wilton , K. P. Ghiggino , D. Phillips , FEBS Lett. 1978, 94, 171. 700131 10.1016/0014-5793(78)80930-2

[84] M. Maccarrone , A. Di Venere , G. van Zadelhoff , G. Mei , G. Veldink , N. Rosato , A. Finazzi‐Agrò , Spectroscopy 2004, 18, 331.

[85] R. K. Fathalla , M. Engel , C. Ducho , Arch. Pharm. 2023, 356, e2300237. 10.1002/ardp.202300237 37464574

[86] A. Berthod , S. Tomer , J. G. Dorsey , Talanta 2001, 55, 69. 18968348 10.1016/s0039-9140(01)00395-2

[87] N. Nishikido , Y. Moroi , R. Matuura , Bull. Chem. Soc. Jpn. 1975, 48, 1387.

[88] W. C. Griffin , J. Soc. Cosmet. Chem. 1949, 1, 311.

[89] D. E. Evans , H. Wennerström , The Colloidal Domain, Wiley‐VCH, New York, NY 1999.

[90] P. Becher , J. Dispersion Sci. Technol. 1984, 5, 81.

[91] I. J. Lin , P. Somasundaran , J. Colloid Interface Sci. 1971, 37, 731.

[92] I. J. Lin , J. P. Friend , Y. Zimmels , J. Colloid Interface Sci. 1973, 45, 378.

[93] C. Rodríguez‐Abreu , J. Surfactants Deterg. 2019, 22, 1001.

[94] Z. C. Li , G. Y. Chen , L. Q. Chen , Y. H. Zhang , Z. Y. Dai , J. Surfactants Deterg. 2019, 22, 731.

[95] J. Slavik , Biochim. Biophys. Acta, Rev. Biomembr. 1982, 694, 1. 10.1016/0304-4157(82)90012-0 6751394

[96] E. M. Kosower , H. Kanety , J. Am. Chem. Soc. 1983, 105, 6236.

[97] A. Upadhyay , T. Bhatt , H. B. Tripathi , D. D. Pant , J. Photochem. Photobiol., A 1995, 89, 201.

[98] G. R. Penzer , Eur. J. Biochem. 1972, 25, 218. 5039838 10.1111/j.1432-1033.1972.tb01687.x

[99] V. Cody , J. Hazel , Biochem. Biophys. Res. Commun. 1976, 68, 425. 1252238 10.1016/0006-291x(76)91162-1

[100] V. Cody , J. Hazel , J. Med. Chem. 1977, 20, 12. 592328 10.1021/jm00222a018

[101] V. Cody , J. Hazel , Acta Crystallogr., Sect. B 1977, 33, 3180.

[102] J. R. Cardinal , P. Mukerjee , J. Phys. Chem. 1978, 82, 1614.

[103] P. Mukerjee , J. R. Cardinal , J. Phys. Chem. 1978, 82, 1620.

[104] R. Nagarajan , E. Ruckenstein , Langmuir 1991, 7, 2934.

[105] B. C. Stephenson , A. Goldsipe , K. J. Beers , D. Blankschtein , J. Phys. Chem. B 2007, 111, 1025. 17266257 10.1021/jp065696i

[106] B. C. Stephenson , A. Goldsipe , K. J. Beers , D. Blankschtein , J. Phys. Chem. B 2007, 111, 1045. 17266258 10.1021/jp065697a

[107] B. C. Stephenson , K. J. Beers , D. Blankschtein , J. Phys. Chem. B 2007, 111, 1063. 17266259 10.1021/jp065699v

[108] P. González‐Tello , F. Camacho , G. Blázquez , J. Chem. Eng. Data 1994, 39, 611.

[109] E. A. Müller , P. Rasmussen , J. Chem. Eng. Data 1991, 36, 214.

[110] I. Regupathi , R. Govindarajan , S. P. Amaresh , T. Murugesan , J. Chem. Eng. Data 2009, 54, 3291.

[111] M. T. Zafarani‐Moattar , A. Salabat , M. Kabiribadr , J. Chem. Eng. Data 1995, 40, 559.

[112] R. Wada , K. Fujimoto , M. Kato , J. Phys. Chem. B 2014, 118, 12223. 25265325 10.1021/jp5048997

[113] R. Kjellander , E. Florin , J. Chem. Soc., Faraday Trans. 1 1981, 77, 2053.

[114] M. J. Blandamer , M. F. Fox , E. Powell , J. W. Stafford , Makromol. Chem. 1969, 124, 222.

[115] T. M. Connor , K. A. McLauchlan , J. Phys. Chem. 1965, 69, 1888.

[116] K. J. Liu , J. L. Parsons , Macromolecules 1969, 2, 529.

[117] J. L. Koenig , A. C. Angood , J. Polym. Sci., Part A‐2 1970, 8, 1787.

[118] J. J. Shephard , P. J. Bremer , A. J. McQuillan , J. Phys. Chem. B 2009, 113, 14229. 19799394 10.1021/jp905149z

[119] K. Tasaki , J. Am. Chem. Soc. 1996, 118, 8459.

[120] G. D. Smith , D. Bedrov , O. Borodin , J. Am. Chem. Soc. 2000, 122, 9548.

[121] U. R. Dahal , E. E. Dormidontova , Phys. Chem. Chem. Phys. 2017, 19, 9823. 28275762 10.1039/c7cp00526a

[122] H. Lee , R. M. Venable , A. D. MacKerell , R. W. Pastor , Biophys. J. 2008, 95, 1590. 18456821 10.1529/biophysj.108.133025 PMC2483782

[123] A. Helenius , K. Simons , Biochim. Biophys. Acta, Rev. Biomembr. 1975, 415, 29. 10.1016/0304-4157(75)90016-7 1091302

[124] F. Müh , J. Rautter , W. Lubitz , Ber. Bunsen‐Ges. 1996, 100, 1974.

[125] L. L. Ge , X. H. Zhang , R. Guo , Polymer 2007, 48, 2681.

[126] U. Patel , N. Dharaiya , J. Parikh , V. K. Aswal , P. Bahadur , Colloids Surf., A 2015, 481, 100.

[127] S. Aktar , M. Saha , S. Mahbub , M. A. Halim , M. A. Rub , M. A. Hoque , D. M. S. Islam , D. Kumar , Y. G. Alghamdi , A. M. Asiri , J. Mol. Liq. 2020, 306, 112880.

[128] P. J. Loll , M. Allaman , J. Wiencek , J. Cryst. Growth 2001, 232, 432.

[129] P. Thiyagarajan , D. M. Tiede , J. Phys. Chem. 1994, 98, 10343.

[130] I. M. Folea , P. Zhang , E. M. Aro , E. J. Boekema , FEBS Lett. 2008, 582, 1749. 18466767 10.1016/j.febslet.2008.04.044

[131] R. Hussein , M. Ibrahim , R. Chatterjee , L. Coates , F. Müh , V. K. Yachandra , J. Yano , J. Kern , H. Dobbek , A. Zouni , Cryst. Growth Des. 2018, 18, 85. 10.1021/acs.cgd.7b00878 PMC6020701 29962903

[132] E. Pettersson , D. Topgaard , P. Stilbs , O. Söderman , Langmuir 2004, 20, 1138. 15803688 10.1021/la035703j

[133] E. Epifanovsky , A. T. B. Gilbert , X. T. Feng , J. Lee , Y. Z. Mao , N. Mardirossian , P. Pokhilko , A. F. White , M. P. Coons , A. L. Dempwolff , Z. T. Gan , D. Hait , P. R. Horn , L. D. Jacobson , I. Kaliman , J. Kussmann , A. W. Lange , K. U. Lao , D. S. Levine , J. Liu , S. C. McKenzie , A. F. Morrison , K. D. Nanda , F. Plasser , D. R. Rehn , M. L. Vidal , Z. Q. You , Y. Zhu , B. Alam , B. J. Albrecht , et al., J. Chem. Phys. 2021, 155, 084801.34470363

[134] W. Humphrey , A. Dalke , K. Schulten , J. Mol. Graphics 1996, 14, 33. 10.1016/0263-7855(96)00018-5 8744570

