# Supplementary material for: Quantifying the Influence of Poly(Ethylene glycol) on the Micelle Formation of Nonionic Detergents
Source: Chempluschem. 2025 Aug 21;90(10):e202500380. doi: 10.1002/cplu.202500380 (PMC12509515; doi:10.1002/cplu.202500380)
Supplement: Supplementary file 1 — Supplementary Material [file CPLU-90-e202500380-s001.pdf]

Supporting Information

Quantifying the Influence of Poly(ethylene glycol) on the Micelle Formation of Nonionic Detergents

Frank Müh,*[a] Julia Gätcke,[b] and Athina Zouni[b]

[a] Johannes Kepler University Linz, Institute for Theoretical Physics, Altenberger Strasse 69, 4040 Linz, Austria
E-mail: frank.mueh@jku.at

[b] Humboldt Universität zu Berlin, Institute for Biology, Leonor-Michaelis-Haus, Philippstrasse 13, 10095 Berlin, Germany

Contents

Supplementary Text 3

ST1: Thermodynamics of Micelle Formation 3

ST2: The Hydrophilic-Lipophilic Balance (HLB) 11

ST3: Equilibrium Constant for the Binding of ANS to Micelles 13

Supplementary Tables 14

Table S1 14

Table S2 15

Table S3 17

Table S4 18

Table S5 19

Table S6 19

Supplementary Figures 20

Figure S1, S2 20

Figure S3, S4 21

Figure S5, S6 22

Figure S7, S8 23

Figure S9, S10 24

Figure S11, S12 25

Figure S13, S14 26

Figure S15, S16 27

Figure S17, S18 28

Figure S19, S20 29

Figure S21, S22 30

Figure S23 31

Figure S24 32

Figure S25, S26, S27 33

Supplementary References 34

Supplementary Text

ST1: Thermodynamics of Micelle Formation

Gibbs versus Helmholtz Free Energy

The Gibbs free energy of the micellar solution is given by the thermodynamic Euler equation

$$G = \sum_j N_j \mu_j \,, \tag{S1}$$

where N_j and μ_j are the particle number and chemical potential, respectively, of the jth molecular species.[1, 2] In the following, we shall derive thermodynamic quantities from a classical statistical description. Since we have constant temperature T and constant pressure P as boundary conditions, we have to use the isothermal-isobaric (NPT) ensemble,[2] whose partition function Δ is related to the Gibbs free energy by

$$G = -k_\mathrm{B} T \ln \Delta \,. \tag{S2}$$

Here, k_B is Boltzmann´s constant, and we will frequently use the abbreviation $\beta = (k_\mathrm{B}T)^{-1}$. The isothermal-isobaric partition function can be written as

$$\Delta(T, P, \{N_j\}) = \frac{1}{\mathcal{V}_0} \int_0^\infty dV \, e^{-\beta PV} \, Q(T, V, \{N_j\}) \,, \tag{S3}$$

where Q is the canonical partition function, V is the volume, and $\mathcal{V}_0$ is a reference volume that serves to make Δ dimensionless.[2, 3] Note that Δ depends on temperature, pressure, and the particle numbers, whereas Q depends on temperature, volume, and the particle numbers. Let us denote the fluctuating volume in the NPT ensemble by V'. If we interpret $\mathcal{V}_0$ as the small range of volume values δV, over which V' fluctuates at the molecular level in thermal equilibrium (small isothermal compressibility of the aqueous solution), and assume that the integrand in Eq. (S3) is nonzero only in this range and adopts in δV approximately a constant value obtained by setting V to the macroscopic volume of the sample, we have

$$\Delta(T, P, \{N_j\}) = \frac{e^{-\beta PV} \, Q(T, V, \{N_j\})}{\delta V} \int_{\delta V} dV' = e^{-\beta PV} \, Q(T, V, \{N_j\}) \tag{S4}$$

and

$$G = -k_\mathrm{B} T \ln Q + PV = F + PV. \tag{S5}$$

with F being the Helmholtz free energy of the micellar solution. The assumptions leading to Eq. (S4) represent the equivalence of the NPT and the canonical (NVT) ensemble in the thermodynamic limit. Then, we obtain the chemical potential of molecular species *i* from

$$\mu_i = \left(\frac{\partial G}{\partial N_i}\right)_{T,P,\{N_j; j \neq i\}} = -k_\mathrm{B} T \left(\frac{\partial \ln Q}{\partial N_i}\right)_{T,V,\{N_j; j \neq i\}} + P \left(\frac{\partial V}{\partial N_i}\right)_{T,P,\{N_j; j \neq i\}} . \tag{S6}$$

Note that we do not assume V to be strictly constant, but to vary only slightly. However, the assumption of ensemble equivalence usually leads to the neglect of the last term on the right-hand side (rhs) of Eq. (S6). We then have

$$\mu_i = -k_\mathrm{B} T \left(\frac{\partial \ln Q}{\partial N_i}\right)_{T,V,\{N_j; j \neq i\}} . \tag{S7}$$

Employing Eq. (S7) implies that we use F instead of G. This is justified at atmospheric pressure and ambient temperatures, where $P\delta V$ is of the order of $0.5\,\mathrm{cal/mole}$.[4] With the temperature of $T = 292.15\,\mathrm{K}$ employed in our experiments, we have

$$e^{-\beta PV'} = e^{-\beta PV} e^{\mp \beta P \delta V} \approx e^{-\beta PV}[1 \mp \beta P \delta V] = e^{-\beta PV}\left[1 \mp \frac{0.5}{580.9}\right] = e^{-\beta PV}[1 \mp 8.6 \cdot 10^{-4}]. \tag{S8}$$

Chemical Potentials in a Model Solution

Before we can write down the canonical partition function, we have to be more specific about the composition of the solution. It is instructive to consider first a simple solution that contains only the solvent water (index W) and two different types of solute (indices 1 and 2). For this simple model system, the Hamiltonian reads

$$\mathcal{H}(x,p) = \mathcal{T}(p) + \mathcal{U}(x). \tag{S9}$$

In Eq. (S9), x and p stand collectively for the positions and conjugate momenta, respectively of all atoms (each having three components per particle), while $\mathcal{T}(p)$ and $\mathcal{U}(x)$ are, respectively, the kinetic and potential energy. We now introduce the $3N_i$-dimensional vectors $\mathbf{q}_i$ and $\mathbf{p}_i$ to represent the center-of-mass (COM) positions and conjugate momenta, respectively, of molecules of the ith type. Furthermore, we will symbolize the orientation-dependence of intermolecular interactions by the dimensionless collective variable w. The dimensionless partition function pertaining to the internal coordinates of a molecule of type i will be denoted by q_i. With $N = N_\mathrm{W} + N_1 + N_2$ and h being Planck´s constant, the canonical partition function for this model solution is then given by

$$Q = \frac{q_\mathrm{W}^{N_\mathrm{W}} q_1^{N_1} q_2^{N_2} \mathcal{Z}}{N_\mathrm{W}!\, N_1!\, N_2!\, h^{3N}} \int d\mathbf{p}_\mathrm{W} d\mathbf{p}_1 d\mathbf{p}_2\, e^{-\beta\mathcal{T}(\mathbf{p}_\mathrm{W},\mathbf{p}_1,\mathbf{p}_2)}, \tag{S10}$$

with the classical configuration integral[5]

$$\mathcal{Z} = \int d\mathbf{q}_\mathrm{W} d\mathbf{q}_1 d\mathbf{q}_2 dw\, e^{-\beta\mathcal{U}(\mathbf{q}_\mathrm{W},\mathbf{q}_1,\mathbf{q}_2;w)} \tag{S11}$$

Now, $\mathcal{T}(\mathbf{p}_\mathrm{W},\mathbf{p}_1,\mathbf{p}_2)$ is the kinetic energy due to the COM motions of all molecules and $\mathcal{U}(\mathbf{q}_\mathrm{W},\mathbf{q}_1,\mathbf{q}_2;w)$ is the interaction energy of molecules depending on distance and orientation. The integration over $\mathbf{p}_i$ ($i = \mathrm{W},1,2$) in Eq. (S10) can be carried out exactly. With the thermal de Broglie wavelength

$$\Lambda_i = \frac{h}{\sqrt{2\pi m_i k_\mathrm{B} T}} \tag{S12}$$

of a molecule of type i with mass m_i, Eq. (S10) becomes

$$Q = \frac{q_\mathrm{W}^{N_\mathrm{W}} q_1^{N_1} q_2^{N_2} \mathcal{Z}}{N_\mathrm{W}!\, N_1!\, N_2!\, \Lambda_\mathrm{W}^{3N_\mathrm{W}} \Lambda_1^{3N_1} \Lambda_2^{3N_2}}. \tag{S13}$$

A suitable way to compute the chemical potential is to consider the free energy difference between an $(N+1)$- and an N-particle system according to Ben-Naim:[6]

$$\mu = \left(\frac{\partial F}{\partial N}\right)_{T,V} = \lim_{dN\to 0}\left[\frac{F(N+dN) - F(N)}{dN}\right] = \frac{F(N+1) - F(N)}{1}. \tag{S14}$$

Then, it follows for the chemical potential of water that

$$\begin{aligned}\exp(-\beta\mu_\mathrm{W}) &= \frac{Q(T,V,N_\mathrm{W}+1,N_1,N_2)}{Q(T,V,N_\mathrm{W},N_1,N_2)} \\ &= \frac{q_\mathrm{W}^{N_\mathrm{W}+1} N_\mathrm{W}!\, \Lambda_\mathrm{W}^{3N_\mathrm{W}} \int d\mathbf{R}_\mathrm{W} d\mathbf{q}_\mathrm{W} d\mathbf{q}_1 d\mathbf{q}_2 dw'\, e^{-\beta\mathcal{U}'(\mathbf{R}_\mathrm{W},\mathbf{q}_\mathrm{W},\mathbf{q}_1,\mathbf{q}_2;w')}}{q_\mathrm{W}^{N_\mathrm{W}} (N_\mathrm{W}+1)!\, \Lambda_\mathrm{W}^{3(N_\mathrm{W}+1)} \int d\mathbf{q}_\mathrm{W} d\mathbf{q}_1 d\mathbf{q}_2 dw\, e^{-\beta\mathcal{U}(\mathbf{q}_\mathrm{W},\mathbf{q}_1,\mathbf{q}_2;w)}}.\end{aligned} \tag{S15}$$

Here, $\mathbf{R}_\mathrm{W}$ is a three-dimensional vector representing the COM position of the added water molecule, while w' now also encompasses the orientation of this water molecule. $\mathcal{U}'(\mathbf{R}_\mathrm{W},\mathbf{q}_\mathrm{W},\mathbf{q}_1,\mathbf{q}_2;w')$ is the potential energy due to intermolecular interactions including the added molecule. The latter may be split into two terms by using the assumption of pairwise additivity of the total potential:[6]

$$\mathcal{U}'(\mathbf{R}_\mathrm{W},\mathbf{q}_\mathrm{W},\mathbf{q}_1,\mathbf{q}_2;w') = \mathcal{U}(\mathbf{q}_\mathrm{W},\mathbf{q}_1,\mathbf{q}_2;w) + \mathcal{B}_\mathrm{W}(\mathbf{R}_\mathrm{W},\mathbf{q}_\mathrm{W},\mathbf{q}_1,\mathbf{q}_2;w'), \tag{S16}$$

where $\mathcal{B}_\mathrm{W}(\mathbf{R}_\mathrm{W},\mathbf{q}_\mathrm{W},\mathbf{q}_1,\mathbf{q}_2;w')$ includes all interactions of the added molecule with the other molecules of the solution. Eq. (S15) then becomes:

$$\exp(-\beta\mu_\mathrm{W}) = \frac{q_\mathrm{W} \int d\mathbf{R}_\mathrm{W} d\mathbf{q}_\mathrm{W} d\mathbf{q}_1 d\mathbf{q}_2 dw'\, e^{-\beta\mathcal{U}(\mathbf{q}_\mathrm{W},\mathbf{q}_1,\mathbf{q}_2;w)} e^{-\beta\mathcal{B}_\mathrm{W}(\mathbf{R}_\mathrm{W},\mathbf{q}_\mathrm{W},\mathbf{q}_1,\mathbf{q}_2;w')}}{(N_\mathrm{W}+1)\Lambda_\mathrm{W}^3 \int d\mathbf{q}_\mathrm{W} d\mathbf{q}_1 d\mathbf{q}_2 dw\, e^{-\beta\mathcal{U}(\mathbf{q}_\mathrm{W},\mathbf{q}_1,\mathbf{q}_2;w)}}. \tag{S17}$$

The next step is to realize that the canonical probability density for quantities depending only on position and orientation is given by

$$P(\mathbf{q}_\mathrm{W},\mathbf{q}_1,\mathbf{q}_2;w) = \frac{e^{-\beta\mathcal{U}(\mathbf{q}_\mathrm{W},\mathbf{q}_1,\mathbf{q}_2;w)}}{\int d\mathbf{q}_\mathrm{W} d\mathbf{q}_1 d\mathbf{q}_2 dw\, e^{-\beta\mathcal{U}(\mathbf{q}_\mathrm{W},\mathbf{q}_1,\mathbf{q}_2;w)}}, \tag{S18}$$

so that

$$\exp(-\beta\mu_\mathrm{W}) = \frac{q_\mathrm{W}}{(N_\mathrm{W}+1)\Lambda_\mathrm{W}^3} \int d\mathbf{R}_\mathrm{W} d\mathbf{q}_\mathrm{W} d\mathbf{q}_1 d\mathbf{q}_2 dw'\; P(\mathbf{q}_\mathrm{W},\mathbf{q}_1,\mathbf{q}_2;w)\, e^{-\beta\mathcal{B}_\mathrm{W}(\mathbf{R}_\mathrm{W},\mathbf{q}_\mathrm{W},\mathbf{q}_1,\mathbf{q}_2;w')}. \tag{S19}$$

Next, we transform to relative coordinates $\bar{\mathbf{q}}_i^{(\mathrm{W})} = \mathbf{q}_i - \mathbf{R}_\mathrm{W}$ and use the fact that $\mathcal{B}_\mathrm{W}$ is actually a function only of the relative coordinates to rewrite the chemical potential as

$$\exp(-\beta\mu_{\mathrm{W}}) = \frac{q_{\mathrm{W}}}{(N_{\mathrm{W}}+1)\Lambda_{\mathrm{W}}^3}\int d\mathbf{R}_{\mathrm{W}} d\bar{\mathbf{q}}_{\mathrm{W}}^{(\mathrm{W})} d\bar{\mathbf{q}}_1^{(\mathrm{W})} d\bar{\mathbf{q}}_2^{(\mathrm{W})} dw'\ P\left(\bar{\mathbf{q}}_{\mathrm{W}}^{(\mathrm{W})}, \bar{\mathbf{q}}_1^{(\mathrm{W})}, \bar{\mathbf{q}}_2^{(\mathrm{W})}; w'\right) e^{-\beta\mathcal{B}_{\mathrm{W}}\left(\bar{\mathbf{q}}_{\mathrm{W}}^{(\mathrm{W})}, \bar{\mathbf{q}}_1^{(\mathrm{W})}, \bar{\mathbf{q}}_2^{(\mathrm{W})}; w'\right)}. \tag{S20}$$

Since the integrand is independent of $\mathbf{R}_{\mathrm{W}}$, we may integrate over $\mathbf{R}_{\mathrm{W}}$ to obtain the volume V. The inner integral is simply the average in the NVT ensemble of the quantity $e^{-\beta\mathcal{B}_{\mathrm{W}}}$. If we symbolize the operation of taking this ensemble average by $\langle \ldots \rangle$, we obtain

$$\exp(-\beta\mu_{\mathrm{W}}) = \frac{q_{\mathrm{W}} V}{(N_{\mathrm{W}}+1)\Lambda_{\mathrm{W}}^3}\langle e^{-\beta\mathcal{B}_{\mathrm{W}}}\rangle. \tag{S21}$$

Since our solution is macroscopic, the number density of water molecules is given by $\rho_{\mathrm{W}} = N_{\mathrm{W}}/V \approx (N_{\mathrm{W}}+1)/V$. We thus obtain finally

$$\mu_{\mathrm{W}} = k_{\mathrm{B}}T\ln\frac{\rho_{\mathrm{W}}\Lambda_{\mathrm{W}}^3}{q_{\mathrm{W}}} - k_{\mathrm{B}}T\ln\langle e^{-\beta\mathcal{B}_{\mathrm{W}}}\rangle. \tag{S22}$$

In a similar way, we obtain for the solutes

$$\mu_i = k_{\mathrm{B}}T\ln\frac{\rho_i\Lambda_i^3}{q_i} - k_{\mathrm{B}}T\ln\langle e^{-\beta\mathcal{B}_i}\rangle \quad (i = 1,2). \tag{S23}$$

Pseudo-Chemical Potentials and Standard Chemical Potentials in the Model Solution

A useful concept introduced by Ben-Naim is the pseudo-chemical potential (PCP).[3, 6] In the present context, we can define it by

$$\tilde{\mu}_i = -k_{\mathrm{B}}T\ln q_i - k_{\mathrm{B}}T\ln\langle e^{-\beta\mathcal{B}_i}\rangle \quad (i = \mathrm{W},1,2), \tag{S24}$$

so that

$$\mu_i = \tilde{\mu}_i + k_{\mathrm{B}}T\ln\rho_i\Lambda_i^3 = \tilde{\mu}_i + k_{\mathrm{B}}T\ln(c_iL_i) \quad (i = \mathrm{W},1,2). \tag{S25}$$

In the last step in Eq. (S25), we introduced the molar concentration (molarity) $c_i = \rho_i/N_{\mathrm{A}}$ of species *i*, where N_{A} is Avogadro´s number, and the quantity $L_i = N_{\mathrm{A}}\Lambda_i^3$, which we may call the "molar kinetic volume" of species i. The difference $\mu_i - \tilde{\mu}_i = k_{\mathrm{B}}T\ln\rho_i\Lambda_i^3$ is also referred to as the *liberation* free energy.

Note that $\tilde{\mu}_i$ encompasses – besides the contributions from internal degrees of freedom (DOFs) of the molecules – all the intermolecular interactions. The latter aspect is made more explicit in a notation introduced by Ben-Naim.[3] The PCP can be derived by considering the addition of a particle to the solution at a fixed position. Then, $-k_{\mathrm{B}}T\ln\langle e^{-\beta\mathcal{B}_i}\rangle$ can be interpreted as the work R to bring the molecule from outside into the solution at this position:

$$\tilde{\mu}_i = R(i|\mathrm{W}+1+2; X_i) - k_{\mathrm{B}}T\ln q_i. \tag{S26}$$

Here, we read $R(i|\mathrm{W}+1+2; X_i)$ as the coupling work of a molecule of type i to the rest of the system composed of W, 1, and 2 with the mole fraction X_i of molecule type i. This notation highlights that, in general, the molecule of type i interacts with all types of molecules in the solution and the term $-k_{\mathrm{B}}T\ln\langle e^{-\beta\mathcal{B}_i}\rangle$ representing this interaction depends on the composition of the solution as typified by the mole fraction X_i. The chemical potential can then we written as

$$\mu_i = R(i|\mathrm{W}+1+2; X_i) + k_{\mathrm{B}}T\ln\frac{\rho_i\Lambda_i^3}{q_i} = R(i|\mathrm{W}+1+2; X_i) + k_{\mathrm{B}}T\ln\frac{c_iL_i}{q_i} \quad (i = \mathrm{W},1,2). \tag{S27}$$

We are now in the position to introduce standard states. Here, we have to distinguish between the solvent and the solutes. For the solvent water, we choose pure water as a reference state. We follow Ben-Naim[3] and use the upper index "p" to label a property of a pure substance. Let $\rho_{\mathrm{W}}^{\mathrm{p}}$ and $c_{\mathrm{W}}^{\mathrm{p}}$ be the number density and molarity, respectively, of pure water at a specified T and P. The chemical potential of pure water is then

$$\mu_{\mathrm{W}}^{\mathrm{p}} = R(\mathrm{W}|\mathrm{W}) + k_{\mathrm{B}}T\ln\frac{\rho_{\mathrm{W}}^{\mathrm{p}}\Lambda_{\mathrm{W}}^3}{q_{\mathrm{W}}} = R(\mathrm{W}|\mathrm{W}) + k_{\mathrm{B}}T\ln\frac{c_{\mathrm{W}}^{\mathrm{p}}L_{\mathrm{W}}}{q_{\mathrm{W}}}, \tag{S28}$$

where $X_{\mathrm{W}} = 1$ is implied. Here, $R(\mathrm{W}|\mathrm{W})$ is the coupling work of one water molecule to its environment, which is pure water. The chemical potential of water in the solution can then be rewritten by adding and subtracting $\mu_{\mathrm{W}}^{\mathrm{p}}$:

$$\begin{aligned}\mu_{\mathrm{W}} &= R(\mathrm{W}|\mathrm{W}+1+2; X_{\mathrm{W}}) + k_{\mathrm{B}}T\ln\frac{c_{\mathrm{W}}L_{\mathrm{W}}}{q_{\mathrm{W}}} + \mu_{\mathrm{W}}^{\mathrm{p}} - \mu_{\mathrm{W}}^{\mathrm{p}} \\ &= \mu_{\mathrm{W}}^{\mathrm{p}} + +k_{\mathrm{B}}T\ln\frac{c_{\mathrm{W}}L_{\mathrm{W}}}{q_{\mathrm{W}}} - k_{\mathrm{B}}T\ln\frac{c_{\mathrm{W}}^{\mathrm{p}}L_{\mathrm{W}}}{q_{\mathrm{W}}} = \mu_{\mathrm{W}}^{\mathrm{p}} + \Delta R_{\mathrm{W}} + k_{\mathrm{B}}T\ln\frac{c_{\mathrm{W}}}{c_{\mathrm{W}}^{\mathrm{p}}}.\end{aligned} \tag{S29}$$

with the abbreviation $\Delta R_{\mathrm{W}} = R(\mathrm{W}|\mathrm{W}+1+2; X_{\mathrm{W}}) - R(\mathrm{W}|\mathrm{W})$. If we introduce the unit volume $V_0 = 1\ \mathrm{L}$ and the standard chemical potential

$$\mu_{\mathrm{W}}^{0} = R(\mathrm{W}|\mathrm{W}) + \Delta R_{\mathrm{W}} + k_{\mathrm{B}}T \ln \frac{L_{\mathrm{W}}}{q_{\mathrm{W}} V_0}, \tag{S30}$$

we can write the chemical potential of the solvent in the usual way:

$$\mu_{\mathrm{W}} = \mu_{\mathrm{W}}^{0} + k_{\mathrm{B}}T \ln(c_{\mathrm{W}} V_0). \tag{S31}$$

Note that the standard chemical potential is different from the chemical potential of pure water. Only in the case of an extremely diluted solution, we can neglect ΔR_{W}, and μ_{W}^{0} becomes equal to $\mu_{\mathrm{W}}^{\mathrm{p}}$ for a unit concentration $c_{\mathrm{W}}^{\mathrm{p}} = 1\ \mathrm{mole/L}$. It should also be noted that the specific form of the standard chemical potential given in Eq. (S30) is valid only, if we use the molarity as the concentration unit.

For the solutes, we have to take a different route. Let solute 1 be the one that we are actually interested in, and for which we have to write down the chemical potential. Solute 2 will be called the cosolute, and it is of interest only for its influence on the chemical potential of solute 1. Let us assume that solute 1 is extremely diluted, so that a molecule of type 1 is practically only surrounded by water and cosolute molecules, whereas a direct encounter of two solute molecules of type 1 can be neglected. Then, the coupling work R for one solute 1 molecule becomes independent of the other solute 1 molecules and the mole fraction X_1, so that the chemical potential of solute 1 can be written as

$$\mu_1 = R(1|\mathrm{W}+2) + k_{\mathrm{B}}T \ln \frac{c_1 L_1}{q_1}. \tag{S32}$$

Note that $X_1 = 1$ is not implied here; rather $X_1 \to 0$. With the unit volume V_0, we can rewrite Eq. (S32) as

$$\mu_1 = \mu_1^{0} + k_{\mathrm{B}}T \ln(c_1 V_0), \tag{S33}$$

where we have introduced the standard chemical potential for the solute according to

$$\mu_1^{0} = R(1|\mathrm{W}+2) + k_{\mathrm{B}}T \ln \frac{L_1}{q_1 V_0}. \tag{S34}$$

This standard state can be interpreted as the infinite-dilution limit for solute 1. However, it refers to an infinite dilution in a mixture of solvent and cosolute, which is explicitly expressed by $R(1|\mathrm{W}+2)$.

Another useful standard state for solute 1 is the infinite-dilution limit in pure water. In the absence of solute 2, the chemical potential of solute 1 at extreme dilution can be written as

$$\mu_1 = R(1|\mathrm{W}) + k_{\mathrm{B}}T \ln \frac{c_1 L_1}{q_1}. \tag{S35}$$

With the standard chemical potential

$$\mu_1^{*} = R(1|\mathrm{W}) + k_{\mathrm{B}}T \ln \frac{L_1}{q_1 V_0}, \tag{S36}$$

we can rewrite Eq. (S35) according to

$$\mu_1 = \mu_1^{*} + k_{\mathrm{B}}T \ln(c_1 V_0). \tag{S37}$$

The influence of solute 2 on the chemical potential of solute 1 can thus be expressed as a change in the standard chemical potential:

$$\mu_1^{0} = \mu_1^{*} + \Delta R_1^{(2)} \tag{S38}$$

with $\Delta R_1^{(2)} = R(1|\mathrm{W}+2) - R(1|\mathrm{W})$. This result is extremely useful for the analysis of cosolute effects on chemical equilibria.

Influence of Solute 2 on the Standard Chemical Potential of Solute 1 in the Model Solution

To find an expression for $\Delta R_1^{(2)}$, we recall, how $R(1|\mathrm{W}+2)$ is defined. By analogy to Eq. (S16), we can split the molecular interaction energy by using the assumption of pairwise additivity according to

$$\mathcal{U}'(\mathbf{R}_1, \mathbf{q}_{\mathrm{W}}, \mathbf{q}_2; w, w_2) = \mathcal{U}(\mathbf{q}_{\mathrm{W}}, \mathbf{q}_2; w, w_2) + \mathcal{B}'_1(\mathbf{R}_1, \mathbf{q}_{\mathrm{W}}, \mathbf{q}_2; w, w_2). \tag{S39}$$

Here, $\mathbf{R}_1$ is the position of solute 1, while w and w_2 are variables representing the orientations of water and solute 2 molecules, respectively. We also assume that no other solute molecule of type 1 needs to be taken into account because of the extreme dilution. Introducing the relative coordinates $\bar{\mathbf{q}}_i^{(1)} = \mathbf{q}_i - \mathbf{R}_1$, we can write

$$R(1|\mathrm{W}+2) = -k_\mathrm{B}T\ln\langle e^{-\beta\mathcal{B}'_1}\rangle \tag{S40}$$

with

$$\langle e^{-\beta\mathcal{B}'_1}\rangle = \int d\bar{\mathbf{q}}_\mathrm{W}^{(1)} d\bar{\mathbf{q}}_2^{(1)} dw\, dw_2\, P\left(\bar{\mathbf{q}}_\mathrm{W}^{(1)}, \bar{\mathbf{q}}_2^{(1)}; w, w_2\right) e^{-\beta\mathcal{B}'_1\left(\bar{\mathbf{q}}_\mathrm{W}^{(1)}, \bar{\mathbf{q}}_2^{(1)}; w, w_2\right)} \tag{S41}$$

and

$$P\left(\bar{\mathbf{q}}_\mathrm{W}^{(1)}, \bar{\mathbf{q}}_2^{(1)}; w, w_2\right) = \frac{e^{-\beta\mathcal{U}\left(\bar{\mathbf{q}}_\mathrm{W}^{(1)}, \bar{\mathbf{q}}_2^{(1)}; w, w_2\right)}}{\int d\bar{\mathbf{q}}_\mathrm{W}^{(1)} d\bar{\mathbf{q}}_2^{(1)} dw\, dw_2\, e^{-\beta\mathcal{U}\left(\bar{\mathbf{q}}_\mathrm{W}^{(1)}, \bar{\mathbf{q}}_2^{(1)}; w, w_2\right)}}. \tag{S42}$$

For the case that no solute of type 2 is present, we obtain accordingly:

$$\mathcal{U}'(\mathbf{R}_1, \mathbf{q}_\mathrm{W}; w) = \mathcal{U}(\mathbf{q}_\mathrm{W}; w) + \mathcal{B}_1(\mathbf{R}_1, \mathbf{q}_\mathrm{W}; w), \tag{S43}$$

$$R(1|\mathrm{W}) = -k_\mathrm{B}T\ln\langle e^{-\beta\mathcal{B}_1}\rangle, \tag{S44}$$

$$\langle e^{-\beta\mathcal{B}_1}\rangle = \int d\bar{\mathbf{q}}_\mathrm{W}^{(1)} dw\; P\left(\bar{\mathbf{q}}_\mathrm{W}^{(1)}; w\right) e^{-\beta\mathcal{B}_1\left(\bar{\mathbf{q}}_\mathrm{W}^{(1)}; w\right)}, \tag{S45}$$

and

$$P\left(\bar{\mathbf{q}}_\mathrm{W}^{(1)}; w\right) = \frac{e^{-\beta\mathcal{U}\left(\bar{\mathbf{q}}_\mathrm{W}^{(1)}; w\right)}}{\int d\bar{\mathbf{q}}_\mathrm{W}^{(1)} dw\, e^{-\beta\mathcal{U}\left(\bar{\mathbf{q}}_\mathrm{W}^{(1)}; w\right)}}. \tag{S46}$$

To evaluate Eq. (S41), we shall make another simplifying conjecture: We assume that the positions and orientations of bulk water molecules are not correlated with those of the solute 2 molecules. Water molecules in the hydration shell of solute 2 may be considered as part of solute 2 for simplicity. Then, the joint probability density $P\left(\bar{\mathbf{q}}_\mathrm{W}^{(1)}, \bar{\mathbf{q}}_2^{(1)}; w, w_2\right)$ in Equation (S42) factorizes according to

$$P\left(\bar{\mathbf{q}}_\mathrm{W}^{(1)}, \bar{\mathbf{q}}_2^{(1)}; w, w_2\right) = P\left(\bar{\mathbf{q}}_\mathrm{W}^{(1)}; w\right) P_2\left(\bar{\mathbf{q}}_2^{(1)}; w_2\right). \tag{S47}$$

Invoking again pairwise additivity of molecular interactions, we can decompose the interaction energy of solute 1 with the solution according to

$$\mathcal{B}'_1\left(\bar{\mathbf{q}}_\mathrm{W}^{(1)}, \bar{\mathbf{q}}_2^{(1)}; w, w_2\right) = \mathcal{B}_1^{(\mathrm{W})}\left(\bar{\mathbf{q}}_\mathrm{W}^{(1)}; w\right) + \mathcal{B}_1^{(2)}\left(\bar{\mathbf{q}}_2^{(1)}; w_2\right). \tag{S48}$$

We are now in the position to evaluate Eq. (S41):

$$\begin{aligned}
\langle e^{-\beta\mathcal{B}'_1}\rangle &= \int d\bar{\mathbf{q}}_\mathrm{W}^{(1)} d\bar{\mathbf{q}}_2^{(1)} dw\, dw_2\, P\left(\bar{\mathbf{q}}_\mathrm{W}^{(1)}, \bar{\mathbf{q}}_2^{(1)}; w, w_2\right) e^{-\beta\mathcal{B}'_1\left(\bar{\mathbf{q}}_\mathrm{W}^{(1)}, \bar{\mathbf{q}}_2^{(1)}; w, w_2\right)} \\
&= \int d\bar{\mathbf{q}}_\mathrm{W}^{(1)} d\bar{\mathbf{q}}_2^{(1)} dw\, dw_2\, P\left(\bar{\mathbf{q}}_\mathrm{W}^{(1)}; w\right) P_2\left(\bar{\mathbf{q}}_2^{(1)}; w_2\right) e^{-\beta\mathcal{B}_1^{(\mathrm{W})}\left(\bar{\mathbf{q}}_\mathrm{W}^{(1)}; w\right)} e^{-\beta\mathcal{B}_1^{(2)}\left(\bar{\mathbf{q}}_2^{(1)}; w_2\right)} \\
&= \int d\bar{\mathbf{q}}_\mathrm{W}^{(1)} dw\, P\left(\bar{\mathbf{q}}_\mathrm{W}^{(1)}; w\right) e^{-\beta\mathcal{B}_1^{(\mathrm{W})}\left(\bar{\mathbf{q}}_\mathrm{W}^{(1)}; w\right)} \int d\bar{\mathbf{q}}_2^{(1)} dw_2\, P_2\left(\bar{\mathbf{q}}_2^{(1)}; w_2\right) e^{-\beta\mathcal{C}_1^{(2)}\left(\bar{\mathbf{q}}_{\widehat{\mathrm{W}}}^{(1)}; \widehat{w}\right)} e^{-\beta\mathcal{B}_1^{(2)}\left(\bar{\mathbf{q}}_2^{(1)}; w_2\right)} \\
&= \langle e^{-\beta\mathcal{B}_1^{(\mathrm{W})}}\rangle_\mathrm{W}\ \langle e^{-\beta\mathcal{B}_1^{(2)}\left(\bar{\mathbf{q}}_2^{(1)}; w_2\right)}\rangle_2,
\end{aligned} \tag{S49}$$

where $\langle\ldots\rangle_i$ denotes an ensemble average over positions and orientations of molecules of type i. Finally, we obtain

$$R(1|\mathrm{W}+2) = -k_\mathrm{B}T\ln\langle e^{-\beta\mathcal{B}_1^{(\mathrm{W})}}\rangle_\mathrm{W} - k_\mathrm{B}T\ln\langle e^{-\beta\mathcal{B}_1^{(2)}}\rangle_2 \tag{S50}$$

and

$$\Delta R_1^{(2)} = -k_\mathrm{B}T\ln\langle e^{-\beta\mathcal{B}_1^{(\mathrm{W})}}\rangle_\mathrm{W} - k_\mathrm{B}T\ln\langle e^{-\beta\mathcal{B}_1^{(2)}}\rangle_2 + k_\mathrm{B}T\ln\langle e^{-\beta\mathcal{B}_1}\rangle_\mathrm{W}. \tag{S51}$$

Note that the ensemble average in Eq. (S44) is actually $\langle\ldots\rangle_\mathrm{W}$. The above conjecture now allows for the first and last term on the rhs of Eq. (S51) to cancel each other. Then,

$$\Delta R_1^{(2)} = -k_\mathrm{B}T\ln\langle e^{-\beta\mathcal{B}_1^{(2)}}\rangle_2. \tag{S52}$$

To simplify the theoretical approach, we shall assume that the probability density $P_2\left(\bar{\mathbf{q}}_2^{(1)}; w_2\right)$ is *symmetric under particle exchange*,[7] which in the present context means that it does not change when we interchange the COM positions and orientations of two solute 2 molecules. Then, we can express the ensemble average $\langle e^{-\beta\mathcal{B}_1^{(2)}}\rangle_2$ in the following way:

$$\langle e^{-\beta\mathcal{B}_1^{(2)}}\rangle_2 = \int d\bar{\mathbf{q}}_2^{(1)}\, dw_2\, P_2\left(\bar{\mathbf{q}}_2^{(1)}; w_2\right) e^{-\beta\mathcal{B}_1^{(2)}\left(\bar{\mathbf{q}}_2^{(1)}; w_2\right)}$$
$$= \left(\int d\bar{\mathbf{r}}_2^{(1)}\, d\breve{w}_2\, p_2\left(\bar{\mathbf{r}}_2^{(1)}; \breve{w}_2\right) e^{-\beta \mathscr{b}_1^{(2)}\left(\bar{\mathbf{r}}_2^{(1)}; \breve{w}_2\right)}\right)^{N_2}. \tag{S53}$$

Here, $p_2\left(\bar{\mathbf{r}}_2^{(1)}; \breve{w}_2\right)$ is a reduced one-particle probability density, and $\mathscr{b}_1^{(2)}\left(\bar{\mathbf{r}}_2^{(1)}; \breve{w}_2\right)$ describes the interaction of the solute 1 molecule with one solute 2 molecule, while the relative position and orientation of the latter are described by $\bar{\mathbf{r}}_2^{(1)}$ and $\breve{w}_2$, respectively. Note that $\bar{\mathbf{r}}_2^{(1)}$ is a 3-dimensional vector. Then,

$$-k_\mathrm{B}T \ln\langle e^{-\beta\mathcal{B}_1^{(2)}}\rangle_2 = N_2 f_1^{(2)} \tag{S54}$$

with the interaction free energy per solute 2 particle

$$f_1^{(2)} = -k_\mathrm{B}T \ln\left(\int d\bar{\mathbf{r}}_2^{(1)}\, d\breve{w}_2\, p_2\left(\bar{\mathbf{r}}_2^{(1)}; \breve{w}_2\right) e^{-\beta \mathscr{b}_1^{(2)}\left(\bar{\mathbf{r}}_2^{(1)}; \breve{w}_2\right)}\right). \tag{S55}$$

We are thus able to express the direct influence of solute 2 on the chemical potential of solute 1 in terms of the particle number N_2 and the one-particle contribution $f_1^{(2)}$. With the definition of the quantity

$$J_{12} = N_\mathrm{A} V f_1^{(2)}, \tag{S56}$$

where N_A is Avogadro´s number and V the volume of the solution, we finally obtain

$$\Delta R_1^{(2)} = c_2 J_{12}, \tag{S57}$$

with c_2 being the molar concentration of solute 2.

Detergent Chemical Potentials in the Micellar Solution and the Micellar Size Distribution

Since our goal is the analysis of polymer effects on micelle formation in a buffered aqueous solution, we have to consider cosolutes besides the various detergent aggregates. To keep the treatment as simple as possible, we consider only the polymer (index P) and the buffer (index B) as cosolutes. However, "B" stands collectively for all buffer ingredients whose concentrations – in contrast to the polymer concentration – are not varied and which thus form a constant background. It is thus sufficient to consider only one hypothetical "buffer molecule" to represent them all. We shall assume that the solution contains N_W water molecules, N_ν detergent aggregates with aggregation number ν (with $\nu = 1$ representing detergent monomers and $\nu > 1$ micelles), N_P polymer molecules and N_B buffer molecules. We consider only one detergent at a time (i. e., we do not consider detergent mixtures). Then, the Gibbs free energy of the micellar solution is given by

$$G = N_\mathrm{W}\mu_\mathrm{W} + N_\mathrm{B}\mu_\mathrm{B} + N_\mathrm{P}\mu_\mathrm{P} + \sum_\nu N_\nu \mu_\nu\,. \tag{S58}$$

We consider micelles to be separate "molecules" that are in equilibrium with detergent monomers. Nonetheless, we will express the micelle properties with respect to monomer quantities. For example, when the molar concentration of micelles is $\hat{c}_\nu$, the molar concentration of detergent that is bound in micelles of size ν is given by $c_\nu = \nu\hat{c}_\nu$. Thus, the "hat" indicates a micelle property, while the "un-hatted" symbols refer to quantities per detergent molecule in the micelle for $\nu > 1$. For $\nu = 1$, there is no difference between the two cases. Note that there are no hats in Equation (S58). The last term on the rhs of Eq. (S68) can be written in two equivalent ways:

$$\sum_\nu N_\nu \mu_\nu = \sum_\nu \hat{N}_\nu \hat{\mu}_\nu\,, \tag{S59}$$

since it holds for the chemical potentials that $\hat{\mu}_\nu = \nu\mu_\nu$ and for the particle numbers that $N_\nu = \nu\hat{N}_\nu$. Note the difference: $\hat{N}_\nu$ is the number of micelles with aggregation number ν, while N_ν is the total number of detergent molecules in all these micelles. The relationship between $\hat{c}_\nu$ and c_ν given above follows accordingly.

The reason for using "un-hatted quantities" at all lies in the derivation of the equilibrium condition for micelle formation.[8] To obtain the micellar size distribution, we have to minimize G under the constraint that the total number of detergent molecules

$$N_\mathrm{det} = \sum_\nu N_\nu = \sum_\nu \nu\hat{N}_\nu \tag{S60}$$

remains constant. The result is that the "un-hatted" chemical potentials are the same for all ν. Specifically, we have

$$\mu_1 = \mu_{\nu>1}. \tag{S61}$$

According to the previous sections, the chemical potential of a micelle can be written as

$$\hat{\mu}_\mathrm{v} = \hat{\mu}_\mathrm{v}^0 + k_\mathrm{B}T\ln(\hat{c}_\mathrm{v}V_0). \tag{S62}$$

The standard chemical potential

$$\hat{\mu}_\mathrm{v}^0 = R(\mathrm{v}|\mathrm{W}+\mathrm{B}+\mathrm{P}) + k_\mathrm{B}T\ln\frac{L_\mathrm{v}}{q_\mathrm{v}V_0} \tag{S63}$$

contains all interactions of the micelle with water, buffer, and polymer encoded in $R(\mathrm{v}|\mathrm{W}+\mathrm{B}+\mathrm{P})$, the contribution from internal DOFs of the micelle in q_v, and the thermal de Broglie wavelength of the micelle in $L_\mathrm{v} = N_\mathrm{A}\Lambda_\nu^3$. To obtain the "un-hatted" chemical potential, we first introduce the quantity

$$\mathcal{A}_\nu = \frac{1}{\nu}[R(\mathrm{v}|\mathrm{W}+\mathrm{B}+\mathrm{P}) - k_\mathrm{B}T\ln q_\mathrm{v}]. \tag{S64}$$

It encompasses all interactions of the micelle with the solution, all interactions between detergent molecules within the micelle as well as all internal DOFs of the detergent molecules, which might be affected by micelle formation. Division by the aggregation number ν does not imply that we decompose all these free energy contributions into single-molecule parts. It merely renders $\mathcal{A}_\nu$ a free energy per detergent molecule in the micelle.

If we neglect the penetration of water or cosolutes into the micelle, the mass of a micelle is given by $\hat{m}_\nu = \nu m_1$, where m_1 is the mass of a detergent monomer. Then,

$$\Lambda_\nu = \frac{h}{\sqrt{2\pi\hat{m}_\nu k_\mathrm{B}T}} = \frac{h}{\sqrt{2\pi\nu m_1 k_\mathrm{B}T}} = \frac{1}{\sqrt{\nu}}\frac{h}{\sqrt{2\pi m_1 k_\mathrm{B}T}} = \frac{\Lambda_1}{\sqrt{\nu}} \tag{S65}$$

at constant T. This result implies that L_v does not scale linearly with the aggregation number:

$$L_\mathrm{v} = N_\mathrm{A}\Lambda_\nu^3 = \frac{N_\mathrm{A}\Lambda_1^3}{\nu^{3/2}} = L_1\nu^{-3/2}. \tag{S66}$$

Putting all this together, we can define the "un-hatted" standard chemical potential as

$$\mu_\mathrm{v}^0 = \frac{\hat{\mu}_\mathrm{v}^0}{\nu} = \frac{1}{\nu}\left[R(\mathrm{v}|\mathrm{W}+\mathrm{B}+\mathrm{P}) - k_\mathrm{B}T\ln q_\mathrm{v} + k_\mathrm{B}T\ln\frac{L_1}{V_0\nu^{3/2}}\right] = \mathcal{A}_\nu + \frac{k_\mathrm{B}T}{\nu}\ln\left(\frac{L_1}{V_0\nu^{3/2}}\right) \tag{S67}$$

and rewrite Equation (S62) according to

$$\mu_\mathrm{v} = \mu_\mathrm{v}^0 + \frac{k_\mathrm{B}T}{\nu}\ln\left(\frac{c_\mathrm{v}V_0}{\nu}\right) \tag{S68}$$

or

$$\mu_\mathrm{v} = \mathcal{A}_\nu + \frac{k_\mathrm{B}T}{\nu}\ln\left(\frac{c_\mathrm{v}L_1}{\nu^{5/2}}\right). \tag{S69}$$

Please, recall that c_v is the molar concentration of detergent molecules bound into micelles with aggregation number ν, but not the micelle concentration. $\mathcal{A}_\nu$ is the quantity that is subject to molecular thermodynamic modeling.[8-10]

Next, we exploit Equation (S61), where now $\mathrm{v} > 1$. From

$$\mathcal{A}_1 + k_\mathrm{B}T\ln(c_1L_1) = \mathcal{A}_\nu + \frac{k_\mathrm{B}T}{\nu}\ln\left(\frac{c_\mathrm{v}L_1}{\nu^{5/2}}\right), \tag{S70}$$

we obtain after some rearrangement

$$\ln\left(\frac{c_\mathrm{v}}{\nu^{5/2}c_1^\nu L_1^{\nu-1}}\right) = -\beta\nu(\mathcal{A}_\nu - \mathcal{A}_1) \tag{S71}$$

and, finally, the micellar size distribution expressed in terms of detergent molarities:

$$c_\mathrm{v} = c_1^\nu \nu^{5/2} L_1^{\nu-1}\exp[-\beta\nu(\mathcal{A}_\nu - \mathcal{A}_1)] \tag{S72}$$

Note that $\mathcal{A}_\nu - \mathcal{A}_1$ is the PCP difference between micelle and monomer per detergent monomer in the micelle. According to the definition in Equation (S64), it has two contributions: (i) the work

$$\frac{1}{\nu}R(\mathrm{v}|\mathrm{W}+\mathrm{B}+\mathrm{P}) - R(1|\mathrm{W}+\mathrm{B}+\mathrm{P}), \tag{S73}$$

required to bring a detergent molecule from a fixed position in the solution into a micelle at a fixed position in the solution, and (ii) the free energy change

$$-\frac{1}{\nu}k_\mathrm{B}T\ln q_\mathrm{v} + k_\mathrm{B}T\ln q_1 \quad\Leftrightarrow\quad -\frac{k_\mathrm{B}T}{\nu}\ln\frac{q_\mathrm{v}}{q_1^\nu} \tag{S74}$$

due to internal DOFs of a detergent molecule and its interactions with other detergent molecules in the micelle upon entering the micelle.

Mass Action Model and Critical Micelle Concentration

A mass action model in the strict sense is a description of micelle formation, in which we allow only for micelles with a fixed aggregation number $\nu = m$. The polydispersity of micelles is then considered negligible. In the context of such a model, it is useful to introduce the following abbreviations:

$$x = c_1 + c_m \tag{S75}$$

$$y = c_1 \tag{S76}$$

$$z = c_m \tag{S77}$$

$$g_{\text{mic}} = \beta(\mathcal{A}_m - \mathcal{A}_1) \tag{S78}$$

Note that the quantities x, y, and z have here a different meaning than in previous work, where they refer to mole fractions.[8, 11, 12] Then, it follows from Equation (S72) that

$$z = y^m m^{5/2} L_1^{m-1} e^{-m g_{\text{mic}}} \tag{S79}$$

In the framework of such a model, we have to assume that the polymer has no effect on m.

According to Bothe et al.,[8] building upon earlier work,[13, 14] the critical micelle concentration (CMC) can be defined by

$$\left(\frac{d^3 y}{dx^3}\right)_{x=\text{CMC}} = 0, \tag{S80}$$

which indicates a breaking point in the curve $y(x)$ corresponding to the monomer concentration as a function of the total detergent concentration. This condition is equivalent to[8]

$$3\left(\frac{d^2 z}{dy^2}\right)^2 = \left(1 + \frac{dz}{dy^3}\right)\frac{d^3 z}{dy^3}. \tag{S81}$$

Using Equation (S79), we obtain from Equation (S81)

$$\left(\frac{y}{x}\right)_{x=\text{CMC}} = \frac{2m^2 - m}{2m^2 - 2}, \tag{S82}$$

which is the ratio of monomer to total detergent concentration at the CMC. With the help of this result, we can derive the relationship between g_{mic} and the CMC:

$$g_{\text{mic}} = \frac{m-1}{m}\ln(\text{CMC}\, L_1) + \tau_m + \frac{5}{2m}\ln m\,. \tag{S83}$$

The term

$$\tau_m = \frac{1}{m}\ln\left\{\frac{(2m^2 - m)^m}{(m-2)(2m^2 - 2)^{m-1}}\right\} \tag{S84}$$

is different from the corresponding term $\tau'_m = \tau_m + m^{-1}\ln m$ found by Bothe et al.[8] and – as we learned recently – also derived earlier by Corkill et al.[15] The reason for the difference is the explicit consideration of the thermal de Broglie wavelength,[12] which is absent in the earlier treatments. The various terms that depend on m only are shown graphically in Figure S1 (see below). It can be seen that the term $(m-1)/m$ goes quickly to unity for increasing m and can thus be neglected.[12] The other terms depending on m go to zero with increasing m, but differently fast and at a slower pace, so that they cannot be neglected in a quantitative modeling analysis of g_{mic} in general.[12] However, as we shall see, they are not of relevance for the analysis of the polymer effect in the framework of the present model. In the limit $m \to \infty$, we obtain the simple relationship

$$g_{\text{mic}} = \ln(\text{CMC}\, L_1)\,, \tag{S85}$$

which – without explicit consideration of L_1 – is often applied[9] and corresponds to a phase-separation model.

To work out the polymer effect, we shall denote the CMC in the absence of polymer as CMC_0 and the corresponding micellization free energy as g^0_{mic}. Within the model of constant m, we then obtain

$$\ln\left(\frac{\text{CMC}}{\text{CMC}_0}\right) = g_{\text{mic}} - g^0_{\text{mic}}. \tag{S86}$$

ST2: The Hydrophilic-Lipophilic Balance (HLB)

Original Definition and Interpretation

The formal concept of the hydrophilic-lipophilic balance (HLB) was introduced by Griffin in 1949.[16] It was intended as a parameter supposed to enable a correlation between the effectiveness of surfactants as emulsifiers and their molecular structures.[17] Although the HLB method is useful as a rough guide in this respect, it has serious limitations as discussed, e. g., by Holmberg et al.[18] The HLB number was originally developed empirically on the basis of nonionic surfactant properties (see below) and later also used as a measure of surfactant film spontaneous curvature.[19] Aveyard[20] points out that "HLB behavior" relates to the whole system and not to surfactant structure alone, so that equations like Equation (S87) below are of very limited use and can be misleading.

The HLB number is defined based on the weight percentage of the hydrophilic moiety w_{hg}:[21]

$$\text{HLB number} = \frac{w_{\mathrm{hg}}}{5} = \frac{100\, M_{\mathrm{hg}}}{5\, M_{\mathrm{tot}}}. \tag{S87}$$

Here, M_{tot} and M_{hg} are the molar masses of the total detergent molecule and of the head group, respectively. Examples are given in Table S2 for oligo(ethylene oxide) monoalkylethers (where hg is the oligo(ethylene oxide) chain) and in Table S3 for the *n*-alkyl-ß-D-maltosides (where hg is the maltose part). Davies introduced a different scale in an attempt to put HLB on a thermodynamic basis and relate this to structural factors. He assigned to each functional group in the detergent molecule a group number (which can be found in text books[18]) and computed HLB according to:[22]

$$\mathrm{HLB} = \sum(\text{hydrophilic group numbers}) - a\ (\text{group number per CH}_2\text{ group}) + 7, \tag{S88}$$

where a is the number of CH_2 groups in the alkyl tail. A thermodynamic perspective of HLB was given by Laughlin.[23]

The H/L Number

According to Shinoda and Friberg,[24] Moore and Bell[25] introduced another method of calculating the hydrophilic-lipophilic balance for oligo(ethylene oxide) monoalkylethers by simply relating the number of OE units b to the number of carbon atoms in the alkyl tail a:

$$\mathrm{H/L\ number} = \frac{100\, b}{a}. \tag{S89}$$

Examples are given in Table S2. We generalized this concept for an application to *n*-alkyl-ß-D-maltosides by replacing b with the number of sugar units 2, so that

$$\mathrm{H/L\ number} = \frac{200}{a}. \tag{S90}$$

Examples are given in Table S3.

Relation to the packing parameter

In their seminal work on the modeling of micelles, Israelachvili et al.[26] introduced a critical packing parameter CPP:[27]

$$\mathrm{CPP} = \frac{v}{A_0 l_c}. \tag{S91}$$

Here, v is the volume of the lipophilic part, which for alkyl chains is given by[28]

$$v = 27.4 + 26.9\, a, \tag{S92}$$

and l_c is roughly equal but less than the fully extended length of the hydrocarbon chain given by[28]

$$l_c = 1.54 + 1.265\, a. \tag{S93}$$

Finally, A_0 is the optimum value of the head group area for packing into the micelle, which is often approximated by the limiting area of the head group in films at the saturated air/liquid interface. We used values for A_0 from Rodríguez-Abreu[27] for oligo(ethylene oxide) monoalkylethers (Table S2) and from Li et al.[29] for *n*-alkyl-ß-D-maltosides (Table S3).

Becher[21] later called the packing parameter "Israelachvilis´s magic number" and noted that it is actually a volume fraction. It relates the actual volume v of the hydrocarbon chain to the volume $A_0 l_c$ it occupies on average taking into account the constraints due to head group packing. Becher suggested to interpret it as a sort of "inverse" HLB. However, we do not think that this interpretation is meaningful, since $A_0 l_c$ is not related to the head group volume. Nonetheless, Becher found a correlation between HLB and CPP for a number of detergents.[21] An important idea originating from such a correlation is that HLB could be related to the curvature of the interface between micellar core and head group region. Such a relation would be in line with the interpretation of the HLB number as a measure of surfactant film spontaneous curvature.[19]

Indeed, we observe a correlation (or, actually, anti-correlation with a negative slope) similar to the finding by Becher[21] for C_aE_b detergents (Figure S11). However, as shown by Rodríguez-Abreu,[27] there is no simple linear relationship for all values of a. Although a reasonable linear fit with a good correlation ($R^2 = 0.9784$) is obtained for $a = 10 - 12$ (with the corresponding values of b listed in Table S2), the data seem to form rather a sigmoidal curve than a straight line. Nonetheless, there is a clear tendency that larger HLB values correspond to smaller packing parameters.

If we restrict the analysis to series of detergents with the same head group, we observe a good correlation ($R^2 = 0.9736$) for alkyl maltosides (Figure S12A) and still a reasonable one ($R^2 = 0.8839$) for C_aE_8 (Figure S12B). Notably, the correlation has a positive slope for the alkyl maltosides in contrast to the negative slope for C_aE_8. This finding is a clear indication that the relationship between HLB number and CPP is not unique and depends significantly on the type of detergent.

According to Israelachvili et al.,[26] based on purely geometric considerations, CPP = 0.33 would indicate a spherical micelle, values between 0.36 and 0.38 elongated globules, and values CPP > 0.4 cylinders. It is known from small-angle X-ray scattering studies that alkyl maltosides form spheroidal globules.[30, 31] The finding that CPP $\leq$ 0.4 for alkyl maltosides ($a = 8 - 14$, Table S3) is in line with this observation. However, while the eccentricity (or ellipticity) of the globules increases with increasing alkyl chain length a,[8] CPP shows the opposite behavior (Table S3).

The correlation between the H/L number and the CPP follows trends similar to those seen for the HLB number. In the case of C_aE_b detergents, the non-linearity of the correlation is even more pronounced (Figure S13), so that we refrained from performing a linear regression. Restricting the analysis to series of detergents with the same head group allows for reasonable linear fits, which again are somewhat better for alkyl maltosides ($R^2 = 0.9781$, Figure S14A) than for C_aE_8 ($R^2 = 0.8335$, Figure S14B).

Relation to the CMC

As noted by Becher,[21] an advance was made in the understanding of HLB by Lin and co-workers,[32, 33] who realized that the logarithm of the CMC can be linearly correlated with the HLB number for homologous series of detergents according to

$$\ln\left(\frac{\mathrm{CMC}}{\mathrm{mM}}\right) = C_1 + C_2\,\mathrm{HLB}. \tag{S94}$$

with constant coefficients C_1 and C_2. It should be noted that Lin et al.[32, 33] used Davies´ concept of HLB based on Equation (S88), whereas we will use Griffin´s original definition based on Equation (S87). For us, the most interesting aspect of this finding is that the HLB number can be related to the micellization free energy. More precisely, we will use Equation (S85) in a slightly modified form (ignoring L_1) and keeping in mind that it is an approximation for large aggregation numbers. We shall interpret g_{mic} as the micellization free energy divided by $k_{\mathrm{B}}T$, which is a dimensionless quantity. (Actually, according to ST1, it is the PCP difference per detergent molecule between micelles and monomers divided by $k_{\mathrm{B}}T$.) Then, following the analysis by Becher,[21] we can write

$$g_{\mathrm{mic}} = C_1 + C_2\,\mathrm{HLB} \tag{S95}$$

or

$$\mathrm{HLB} = D_1 + D_2 g_{\mathrm{mic}}. \tag{S96}$$

with constant coefficients $D_1 = -C_1/C_2$ and $D_2 = 1/C_2$. In this way, the HLB number can be related to the driving force for micelle formation. Please, recall that given values for the coefficients C_1 and C_2 are valid only for one homologous series of detergents.

As is apparent from Figure S15, there is no simple correlation between $\ln(\mathrm{CMC/mM})$ and HLB for C_aE_b detergents in general. However, restriction to homologous series of detergents with fixed head group allows for a reasonable fit to Equation (S94). Notably, the values for $C_2 \approx 3.4$ obtained from the fits and the high quality of the fits are very similar for alkyl maltosides ($R^2 = 0.9833$, Figure ST10A) and for C_aE_8 ($R^2 = 0.9854$, Figure ST10B).

It can be seen from Figures S17 and S18 that similar results are obtained from correlating $\ln(\mathrm{CMC/mM})$ with the H/L number. However, in this case, the restriction to homologous series of detergents with fixed head group does not result in a linear correlation (Figure S18).

ST3: Equilibrium Constant for the Binding of ANS to Micelles

We make the simplifying assumption that at most one molecule of ANS binds to a micelle. Let $[\mathrm{A}]$, $[\mathrm{M}]$, and $[\mathrm{AM}]$ be the molar concentration, respectively, of free ANS (not bound to micelles), ANS-free micelles, and micelles with an ANS molecule bound. Then the equilibrium constant for binding of ANS to micelles can be written as

$$K = \frac{[\mathrm{AM}]}{[\mathrm{A}]\,[\mathrm{M}]}\,, \tag{S97}$$

where all concentrations are supposed to be given in mM. Recall that z is the molar concentration of detergent in micelles, so that

$$[\mathrm{M}] = \frac{z}{m}, \tag{S98}$$

with m being the aggregation number. The fraction f of ANS molecules bound to micelles is given by

$$f = \frac{[\mathrm{AM}]}{[\mathrm{A}] + [\mathrm{AM}]} = \frac{[\mathrm{A}]\,[\mathrm{M}]\,K}{[\mathrm{A}] + [\mathrm{A}]\,[\mathrm{M}]\,K} = \frac{[\mathrm{M}]\,K}{1 + [\mathrm{M}]\,K} = \frac{\frac{z}{m}\,K}{1 + \frac{z}{m}\,K} = \frac{z\,K}{m + z\,K}\,. \tag{S99}$$

The fluorescence intensity ϕ observed at 480 nm is given by

$$\phi = \phi_0(1 - f) + \phi_\infty f, \tag{S100}$$

where $\phi_0 = c_0$ and ϕ_∞ are the fluorescence intensities for free and micelle-bound ANS, respectively, assumed to be independent of the detergent concentration. If we introduce the quantities $I = \phi - c_0$ and $\Delta\phi = \phi_\infty - c_0$, we see that I is proportional to f:

$$I = \phi_0(1 - f) + \phi_\infty f - c_0 = (\phi_\infty - c_0)f = \Delta\phi\, f. \tag{S101}$$

For large m, we have the approximation:[8]

$$z \approx x - \mathrm{CMC}. \tag{S102}$$

Note that in this limit, the CMC corresponds to a sharp breaking point in the curve. Hence, we can shift the titration curve $\phi(x)$ and compute the initial slope of the curve right above the CMC according to

$$b_1 = \left(\frac{dI}{dx}\right)_{x \gtrsim \mathrm{CMC}} \approx \Delta\phi \left(\frac{df}{dz}\right)_{z=0}. \tag{S103}$$

Since

$$\frac{df}{dz} = \frac{m\,K}{(m + z\,K)^2}\,, \tag{S104}$$

we obtain

$$b_1 = \Delta\phi \frac{K}{m}. \tag{S105}$$

We note that a similar equation was derived by Abuin e al.,[34] but neglecting m and assuming $\Delta\phi \approx \phi_\infty$. According to Equation (S105), we can compute the binding constant K from the parameters b_1 and c_0 obtained from the partial linear regressions of the titration curves:

$$K = \frac{m b_1}{\phi_\infty - c_0}. \tag{S106}$$

The titration curves do not reach the value of ϕ_∞ in the presence of PEG (see Figure S4A of the main text). However, if the experimental titration curves obey Equation (S101) with the functional form of f given in Equation (S99), the effect of PEG on the shape of the curve above the CMC is entirely due to a change of K, and since $f \to 1$ for $z \to \infty$ (see the model curves in Figure ST13), ϕ_∞ should be independent of the PEG concentration. To check this conjecture and obtain information about ϕ_∞, we can exploit the fact that I^{-1} is proportional to z^{-1} according to

$$\frac{1}{I} = \frac{1}{\Delta\phi}\frac{m + z\,K}{z\,K} = \frac{m}{\Delta\phi\,K}\frac{1}{z} + \frac{1}{\Delta\phi}. \tag{S107}$$

A double-reciprocal plot should provide $\Delta\phi^{-1}$ as intercept and a slope s that is related to K by

$$K = \frac{m}{\Delta\phi\, s}. \tag{S108}$$

The problem remains that K can only be determined in this way, if m is known. Any uncertainty in the aggregation number has an impact on the quantification of the equilibrium constant for the binding of ANS to micelles.

Supplementary Tables

Table S1. Values of the coefficients a_0, b_1, and c_0 obtained from linear regressions of titration curves to determine the CMC (in mM).

χ / % (w/v)	DDM									DM					
	PEG400			PEG2000			PEG4000			PEG400			PEG4000		
	a_0	b_1	c_0	a_0	b_1	c_0	a_0	b_1	c_0	a_0	b_1	c_0	a_0	b_1	c_0
0	-61.3 ± 1.3	461 ± 6	2.67 ± 0.06	–	–	–	–	–	–	-376 ± 11	204 ±5	2.95 ± 0.08	–	–	–
5	-35.9 ± 1.0	251 ± 4	8.49 ± 0.06	-27.0 ± 0.9	212 ± 3	11.85 ± 0.07	-19.9 ± 0.4	196.1 ± 1.5	14.1 ± 0.1	-217 ± 5	115 ± 2	7.69 ± 0.13	-174 ± 7	88 ± 3	13.8 ± 0.1
10	-18.5 ± 1.2	154 ± 3	13.26 ± 0.05	-3.9 ± 0.5	119 ± 1	20.0 ± 0.1	-2.9 ± 0.6	122.8 ± 1.5	22.4 ± 0.1	-158 ± 9	77 ± 3	13.5 ± 0.2	-111 ± 10	56 ± 4	21.0 ± 0.2
15	1.0 ± 0.8	89.0 ± 1.1	22.23 ± 0.08	11.2 ± 1.5	72 ± 5	28.4 ± 0.1	–	–	–	-113 ± 3	48.4 ± 0.6	19.92 ± 0.13	-63 ± 3	35.0 ± 0.7	30.5 ± 0.2
20	13.1 ± 0.9	63.9 ± 1.2	32.10 ± 0.09	–	–	–	–	–	–	-78 ± 3	34.7 ± 0.6	28.66 ± 0.12	-30 ± 2	25.5 ± 0.4	40.60 ± 0.13
25	–	–	–	–	–	–	–	–	–	-40 ± 2	24.1 ± 0.5	41.2 ± 0.1	–	–	–
30	–	–	–	–	–	–	–	–	–	-7 ± 4	16.0 ± 0.9	55.7 ± 0.1	–	–	–

Table S1 (continued).

χ / % (w/v)	$C_{10}E_8$						$C_{12}E_8$						$C_{12}E_{10}$					
	PEG400			PEG4000			PEG400			PEG4000			PEG400			PEG4000		
	a_0	b_1	c_0	a_0	b_1	c_0	a_0	b_1	c_0	a_0	b_1	c_0	a_0	b_1	c_0	a_0	b_1	c_0
0	-230 ± 13	259 ± 10	3.0 ± 0.1	–	–	–	-37.1 ± 0.9	566 ± 6	2.89 ± 0.07	–	–	–	-19.7 ± 0.9	802 ± 13	3.2 ± 0.4	–	–	–
5	-156 ± 5	148 ± 3	7.6 ± 0.1	-139 ± 3	132 ± 2	13.5 ± 0.1	-23.0 ± 0.8	334 ± 4	7.89 ± 0.07	-12.4 ± 0.8	271 ± 4	13.53 ± 0.17	-5.4 ± 0.5	415 ± 6	8.0 ± 0.3	-0.9 ± 0.4	377 ± 4	14.3 ± 0.2
10	-121 ± 8	106 ± 5	12.8 ± 0.2	-102 ± 7	94 ± 4	21.5 ± 0.4	-6.2 ± 0.8	187 ± 4	14.19 ± 0.13	3.1 ± 0.5	163 ± 3	23.57 ± 0.12	1.5 ± 0.6	269 ± 5	14.0 ± 0.2	10.4 ± 0.5	228 ± 4	21.1 ± 0.2
15	-78 ± 4	71 ± 2	20.1 ± 0.1	-37 ± 5	54 ± 3	30.3 ± 0.2	6.8 ± 0.8	109 ± 4	21.56 ± 0.11	18.8 ± 1.2	90 ± 5	30.98 ± 0.23	12.3 ± 0.6	158 ± 4	20.5 ± 0.3	23.4 ± 0.5	153 ± 4	30.3 ± 0.1
20	-51 ± 2	52 ± 1	28.5 ± 0.2	-19 ± 3	45 ± 2	41.2 ± 0.2	16.6 ± 0.8	81 ± 3	30.23 ± 0.14	31.8 ± 0.8	64 ± 3	41.81 ± 0.14	23.5 ± 0.5	112 ± 2	31.3 ± 0.2	39.0 ± 0.7	100 ± 5	43.9 ± 0.2
25	-27 ± 1	38.4 ± 0.4	40.6 ± 0.3	13 ± 4	25 ± 2	56.5 ± 0.3	31.0 ± 0.3	51 ± 1	40.86 ± 0.14	49 ± 2	36 ± 6	54.2 ± 0.2	36.8 ± 0.5	76 ± 2	42.9 ± 0.3	52.5 ± 0.3	63 ± 1	56.4 ± 0.3
30	11 ± 4	25 ± 2	57.4 ± 0.3	40 ± 4	21 ± 2	74.4 ± 0.4	49.4 ± 0.8	38 ± 1	58.45 ± 0.18	–	–	–	54.6 ± 0.2	53 ± 1	58.9 ± 0.3	71.7 ± 0.5	41 ± 2	73.9 ± 0.2

Table S2. Properties of some oligo(ethylene glycol) monoalkylether detergents C_aE_b compiled from the literature.

a	*b*	CMC / mM	HLB number i)	H/L number j)	aggregation number	packing parameter p)
10	5	0.69 a), 0.86 w)	12.5	50.0	–	0.39
	6	0.83 a), 0.90 f), 0.46 k), 0.83 s), 0.90 t), 0.92 w)	13.3	60.0	73 k), 76 l), 78 s), 73 u)	0.38
	7	0.88 a), 0.96 f)	14.0	70.0	60 m)	–
	8	0.92 a), 1.2 b), 1.0 c), 1.03 f), 0.79 g), 0.28 k), 1.03 q), 1.1 s), 1.0 x)	14.5	80.0	50 m), 70 n) 57 s)	0.30
11	8	0.35 b), 0.30 c) x)	14.1	72.7	–	0.32
12	2	0.033 d)	7.7	16.7	–	0.60
	3	0.052 d)	9.4	25.0	–	0.50
	4	0.047 a), 0.064 d), 0.046 y)	10.7	33.3	–	0.46
	5	0.049 a), 0.064 d) y), 0.045 e), 0.068 g), 0.071 v), 0.04 w)	11.7	41.7	160 n), 1940 z)	0.42
	6	0.064 a), 0.060 e), 0.069 g), 0.065 k), 0.087 t) v) y), 0.082 w)	12.5	50.0	105 k) l), 103 y)	0.40
	7	0.067 a), 0.082 d), 0.069 g)	13.2	58.3	90 z)	0.37
	8	0.067 a), 0.083 b), 0.071 c) x), 0.109 d) y), 0.070 g), 0.110 h), 0.056 k), 0.081 r)	13.7	66.7	120 k) l), 127 z)	0.32
	10	0.09 e)	14.6	83.3	–	–
	12	0.093 h)	15.3	100.0	81 l)	
	15	0.120 e)	16.0	125.0	–	–
	18	0.083 h)	16.5	150.0	–	–
	20	0.155 e)	16.8	166.7	–	–
	23	0.091 h), 0.090 o),	17.2	191.7	40 l) o)	–
	29	0.260 e)	17.7	241.7	–	–
	49	0.630 e)	18.6	408.3	–	–
13	8	0.028 b), 0.027 c) x)	13.4	61.5	–	0.36
	10	0.125 h)	14.3	76.9	–	0.23
	15	0.150 h)	15.7	115.4	–	–
	22	0.196 h)	16.9	169.2	–	–
14	5	0.009 a) x)	10.9	35.7	–	–
14	8	0.0098 b), 0.0090 c), 0.0063 g), 0.0052 k)	13.0	57.1	–	0.44
15	8	0.0037 b), 0.0035 c) x)	12.7	53.3	–	0.46
16	8	0.00056 g), 0.00047 k)	12.4	50.0	–	–
18	8	0.00005 g)	11.9	44.4	–	–

a) Determined with the ANS method[35] (probably at room temperature) by Van Ede et al.[36] (as cited by Hinze and Pramauro[37]).

b) Meguro et al.,[38] obtained from surface tension curves at 20 °C.

c) Meguro et al.,[38] obtained from surface tension curves at 25 °C.

d) Rosen et al.,[39] obtained from surface tension curves at 25 °C.

e) Nishikido et al.,[40] obtained from surface tension curves as well as changes of the absorption spectrum of pinacyanol chloride at 20 °C.

f) Funasaki et al.,[41] obtained from surface tension curves at 25 °C.

g) Hidaki et al.,[42] CMC values at 20 °C from the data supplied by the manufacturer.

h) Moroi and Matuura,[43] cited from Becher without specifying the method.

i) Calculated on the basis of Equation (S87).

j) Computed on the basis of Equation (S89) according to Moore and Bell[25] as cited by Shinoda and Friberg.[24]

k) Casey and Reithmeier;[44] CMC determined with the ANS method under conditions for size exclusion chromatography at room temperature; aggregation numbers cited from Neugebauer (product information from Calbiochem, New York 1987).

l) Tanford and Reynolds.[45]

m) Funasaki et al.,[41, 46] obtained from gel filtration chromatography.

n) As given by Hinze and Pramauro.[37]

o) Borgerding and Hinze,[47] CMC value obtained from surface tension curves (probably at room temperature); aggregation number from cited literature.

p) Computed from Equation (S91) based on Equations (S92) and (S93) and values of A_0 from Rodríguez-Abreu.[27]

q) Liljekvist and Kronberg,[48] obtained from surface tension curves in 0.01 M NaCl at 22 °C.

r) Drummond et al.,[49] obtained from surface tension curves at 25 °C.

s) Kroll et al.,[50] static light scattering at 25 °C.

t) Corkill et al.,[15] obtained from surface tension curves at 25 °C.

u) Balmbra et al.,[51] light scattering.

v) Schubert et al.,[52] obtained from surface tension curves at 25 °C.

w) Donbrow and Jan,[53] obtained from surface tension curves at 20 °C.

x) Ueno et al.,[54] obtained from surface tension curves at 25 °C.

y) Herrington and Sahi;[55] CMC obtained from surface tension curves at 25 °C; aggregation number from vapor pressure osmometry at 25 °C.

z) Brown et al.,[56] quasi-elastic light scattering at 20.4 °C ($C_{12}E_5$), 19.5 °C ($C_{12}E_7$), and 20.9 °C ($C_{12}E_8$).

Table S3. Properties of *n*-alkyl-ß-D-maltosides with a carbon atoms in the alkyl tail compiled from the literature.

a	CMC / mM	HLB number [u)]	H/L number [v)]	aggregation number	packing parameter [w)]
8	26.5 [a)], 19.5 [c)], 23.3 [f)]	15.0	25.0	47 [c)], 55 [s)]	0.40
9	6.3 [a)], 6.0 [c)], 7.6 [f)]	14.6	22.2	55 [c)], 66 [s)]	0.38
10	2.2 [a)], 1.8 [d)], 2.1 [f)], 1.3 [g)], 2.0 [i)], 2.0 [k)], 1.9-2.6 [t)]	14.1	20.0	69 [d)], 82-90 [l)], 81 [s)]	0.34
11	0.59 [d)], 0.64 [f)], 0.38 [g)],	13.7	18.2	71 [c)], 105 [s)]	–
12	0.16 [b)], 0.152 [d)], 0.17 [f)], 0.09 [g)], 0.185 [h)], 0.15 [j)], 0.165 [n)], 0.16-0.25 [t)], 0.179 [x)]	13.4	16.7	90 [d)], 125 [h)], 135-145 [l)], 140 [m)], 100-150 [n)], 132 [o)], 138 [p)], 98 [q)], 111 [r)], 125 [s)], 148 [t)]	0.32
13	0.033 [d)], 0.024 [e)]	13.0	15.4	186 [c)], 145 [s)]	–
14	0.01 [d)]	12.7	14.3	–	0.29

a) Alpes et al.,[57] determined by fluorescence enhancement of 1,6-diphenyl-1,3,5-hexatriene.

b) De Grip and Bovee-Geurts,[58] determined by fluorescence enhancement of TNS.

c) Anatrace, 100 mM NaCl, 20 mM HEPES pH 7.5.

d) Anatrace, H_2O.

e) Anatrace, 0.15 mM NaCl.

f) Tsamaloukas et al.,[59] isothermal titration calorimetry at 25 °C.

g) Müh et al.,[10] determined by fluorescence enhancement of ANS as well as I_1/I_3 ratio of pyrene in 100 mM PIPES (pH 7.0), 5 mM $CaCl_2$.

h) Aoudia and Zana;[60] CMC obtained from the I_1/I_3 ratio of pyrene at 25 °C; aggregation number from fluorescence quenching.

i) Liljekvist and Kronberg,[48] obtained from surface tension curves in 0.01 M NaCl at 22 °C.

j) Drummond et al.,[49] obtained from surface tension curves at 25 °C.

k) Aveyard et al.,[61] obtained from surface tension curves at 25 °C.

l) Lipfert et al.,[30] small-angle X-ray scattering.

m) Strop and Brunger,[62] size exclusion chromatography.

n) VanAken et al.;[63] CMC determined *inter alia* by fluorescence enhancement of TNS; aggregation numbers from size exclusion chromatography and sedimentation equilibrium analysis.

o) Dupuy et al.,[64] small-angle X-ray scattering.

p) Tummino and Gafni,[65] fluorescence quenching.

q) Rosevear et al.,[66] size exclusion chromatography.

r) Warr et al.,[67] fluorescence quenching.

s) Kunji et al.,[68] size exclusion chromatography.

t) Jumpertz et al.;[69] CMC from fluorescence enhancement; aggregation numbers from multiple angle light scattering.

u) Calculated on the basis of Equation (S87).

v) Computed on the basis of Equation (S90).

w) Computed from Equation (S91) based on Equations (S92) and (S93) and values of A_0 from Li et al.[29]

x) Wang et el.,[70] obtained from surface tension curves at 25 °C.

Table S4. Values of the intercept $\Delta\phi^{-1}$, the slope s according to Equation (S108) in mM, and R^2 of the linear regressions for double-reciprocal plots $I^{-1}(z^{-1})$ (cf. Figure 10B,C of the main text and Figure S23) obtained from the fluorescence titration curves for the various detergent-PEG combinations.

χ / % (w/v)	**DDM**									**DM**					
	PEG400			PEG2000			PEG4000			PEG400			PEG4000		
	$\Delta\phi^{-1}\cdot 10^3$	$s\cdot 10^3$	R^2	$\Delta\phi^{-1}\cdot 10^3$	$s\cdot 10^3$	R^2	$\Delta\phi^{-1}\cdot 10^3$	$s\cdot 10^3$	R^2	$\Delta\phi^{-1}\cdot 10^3$	$s\cdot 10^3$	R^2	$\Delta\phi^{-1}\cdot 10^3$	$s\cdot 10^3$	R^2
0	1.0 ± 0.3	2.2 ± 0.03	0.9976	–	–	–	–	–	–	2.0 ± 0.1	4.5 ± 0.06	0.9956	–	–	–
5	2.0 ± 0.4	3.7 ± 0.04	0.9886	1.0 ± 0.3	4.9 ± 0.04	0.9988	1.0 ± 0.3	5.1 ± 0.03	0.9995	1.1 ± 0.2	8.5 ± 0.1	0.9936	1.1 ± 0.3	11.7 ± 0.2	0.9936
10	-0.03 ± 1.0	7.1 ± 0.1	0.9957	0.9 ± 0.7	8.1 ± 0.1	0.9988	1.5 ± 0.3	7.9 ± 0.05	0.9998	1.7 ± 0.8	12.3 ± 0.4	0.9716	0.8 ± 0.4	18.1 ± 0.3	0.9955
15	-0.6 ± 0.7	12.4 ± 0.1	0.9990	–	–	–	–	–	–	0.2 ± 0.5	21.2 ± 0.3	0.9974	0.7 ± 0.4	28.4 ± 0.4	0.9973
20	1.0 ± 0.4	15.3 ± 0.1	0.9996	–	–	–	–	–	–	1.7 ± 0.7	27.3 ± 0.6	0.9931	1.2 ± 0.3	38.0 ± 0.3	0.9989
25	–	–	–	–	–	–	–	–	–	1.9 ± 0.6	39.4 ± 0.4	0.9986	–	–	–
30	–	–	–	–	–	–	–	–	–	-0.8 ± 1.7	64.2 ± 1.5	0.9959	–	–	–

Table S4 (continued).

χ / % (w/v)	**$C_{10}E_8$**						**$C_{12}E_8$**						**$C_{12}E_{10}$**					
	PEG400			PEG4000			PEG400			PEG4000			PEG400			PEG4000		
	$\Delta\phi^{-1}\cdot 10^3$	$s\cdot 10^3$	R^2	$\Delta\phi^{-1}\cdot 10^3$	$s\cdot 10^3$	R^2	$\Delta\phi^{-1}\cdot 10^3$	$s\cdot 10^3$	R^2	$\Delta\phi^{-1}\cdot 10^3$	$s\cdot 10^3$	R^2	$\Delta\phi^{-1}\cdot 10^3$	$s\cdot 10^3$	R^2	$\Delta\phi^{-1}\cdot 10^3$	$s\cdot 10^3$	R^2
0	2.2 ± 0.2	3.2 ± 0.1	0.9636	–	–	–	1.8 ± 0.2	1.7 ± 0.02	0.9983	–	–	–	1.5 ± 0.2	1.2 ± 0.01	0.9983	–	–	–
5	2.1 ± 0.2	5.8 ± 0..2	0.9852	2.1 ± 0.2	6.9 ± 0.2	0.9906	0.7 ± 0.3	3.0 ± 0.03	0.9986	0.2 ± 0.6	3.9 ± 0.04	0.9974	0.4 ± 0.4	2.4 ± 0.03	0.9979	2.0 ± 0.6	2.5 ± 0.04	0.9961
10	2.0 ± 0.2	8.8 ± 0.1	0.9956	1.8 ± 0.3	10.6 ± 0.3	0.9900	1.5 ± 0.5	5.1 ± 0.06	0.9968	4.0 ± 0.8	5.7 ± 0.05	0.9984	3.0 ± 1.2	3.4 ± 0.07	0.9922	3.1 ± 1.3	4.1 ± 0.07	0.9936
15	1.6 ± 0.3	13.5 ± 0.2	0.9966	1.7 ± 0.4	17.8 ± 0.3	0.9961	3.2 ± 1.5	8.7 ± 0.2	0.9920	8.5 ± 5.2	9.6 ± 0.4	0.9917	1.3 ± 3.9	6.3 ± 0.2	0.9765	2.1 ± 1.3	6.3 ± 0.1	0.9950
20	1.6 ± 0.2	18.6 ± 0.2	0.9987	1.8 ± 0.6	23.2 ± 0.4	0.9946	-0.3 ± 1.5	13.3 ± 0.2	0.9936	4.2 ± 2.4	15.1 ± 0.3	0.9924	6.9 ± 4.2	8.4 ± 0.3	0.9813	4.6 ± 0.2	9.5 ± 0.2	0.9894
25	2.4 ± 0.2	24.0 ± 0.1	0.9996	1.3 ± 1.1	42.5 ± 0.9	0.9945	1.5 ± 2.8	19.6 ± 0.6	0.9854	–	–	–	4.0 ± 14.2	12.2 ± 0.8	0.9206	–	–	–
30	1.9 ± 1.4	36.9 ± 1.3	0.9847	-2.0 ± 2.4	57.7 ± 1.7	0.9876	3.5 ± 3.2	25.2 ± 0.7	0.9862	–	–	–	2.4 ± 3.3	18.3 ± 0.4	0.9930	–	–	–

Table S5. Estimates of $\Delta\phi$ (a.u.), i. e. the limit of $\phi_{480} - c_0$ for $x \to \infty$, for the various detergents investigated.

Detergent	$\Delta\phi$ estimated from titration curve [a)]	$\Delta\phi$ estimated from double-reciprocal plot [b)]
DM	370	841
DDM	470	938
$C_{10}E_8$	350	552
$C_{12}E_8$	350	332
$C_{12}E_{10}$	420	419

[a)] Estimated from the asymptotic behavior of the titration curve for zero PEG concentration.

[b)] Determined from the fit values of $\Delta\phi^{-1}$ (intercept of double-reciprocal plot, cf. Table S4) by averaging the data pertaining to fits, where $R^2 \geq 0.9900$ and the intercept is not negative. In this procedure, it is assumed that PEG does not affect $\Delta\phi$.

Table S6. Values of K/m and K obtained from Equation (13) of the main text to characterize the binding of ANS to the micelles of the various detergents as a function of PEG concentration χ. We used $m = 70$ for detergents with $a = 10$ and $m = 120$ for $a = 12$.

χ / % (w/v)	**DDM**						**DM**			
	PEG400		PEG2000		PEG4000		PEG400		PEG4000	
	K/m	K	K/m	K	K/m	K	K/m	K	K/m	K
0	0.98	118	–	–	–	–	0.55	39	–	–
5	0.53	64	0.45	54	0.42	50	0.31	22	0.24	17
10	0.33	39	0.25	30	0.26	31	0.21	15	0.15	11
15	0.19	23	0.15	18	–	–	0.13	9	0.09	7
20	0.14	16	–	–	–	–	0.09	7	0.07	5
25	–	–	–	–	–	–	0.07	5	–	–
30	–	–	–	–	–	–	0.04	3	–	–

Table S6 (continued).

χ / % (w/v)	**$C_{10}E_8$**				**$C_{12}E_8$**				**$C_{12}E_{10}$** [a)]			
	PEG400		PEG4000		PEG400		PEG4000		PEG400		PEG4000	
	K/m	K	K/m	K	K/m	K	K/m	K	K/m	K	K/m	K
0	0.74	52	–	–	1.62	194	–	–	1.91	229	–	–
5	0.42	30	0.38	26	0.95	115	0.77	93	0.99	119	0.90	108
10	0.30	21	0.27	19	0.53	64	0.47	56	0.64	77	0.54	65
15	0.20	14	0.15	11	0.31	37	0.26	31	0.38	45	0.36	44
20	0.15	10	0.13	9	0.23	28	0.18	22	0.27	32	0.24	29
25	0.11	8	0.07	5	0.15	17	0.10	12	0.18	22	0.15	18
30	0.07	5	0.06	4	0.11	13	–	–	0.13	15	0.10	12

[a)] K computed with the assumption $m = 120$ lacking experimental evidence.

Supplementary Figures

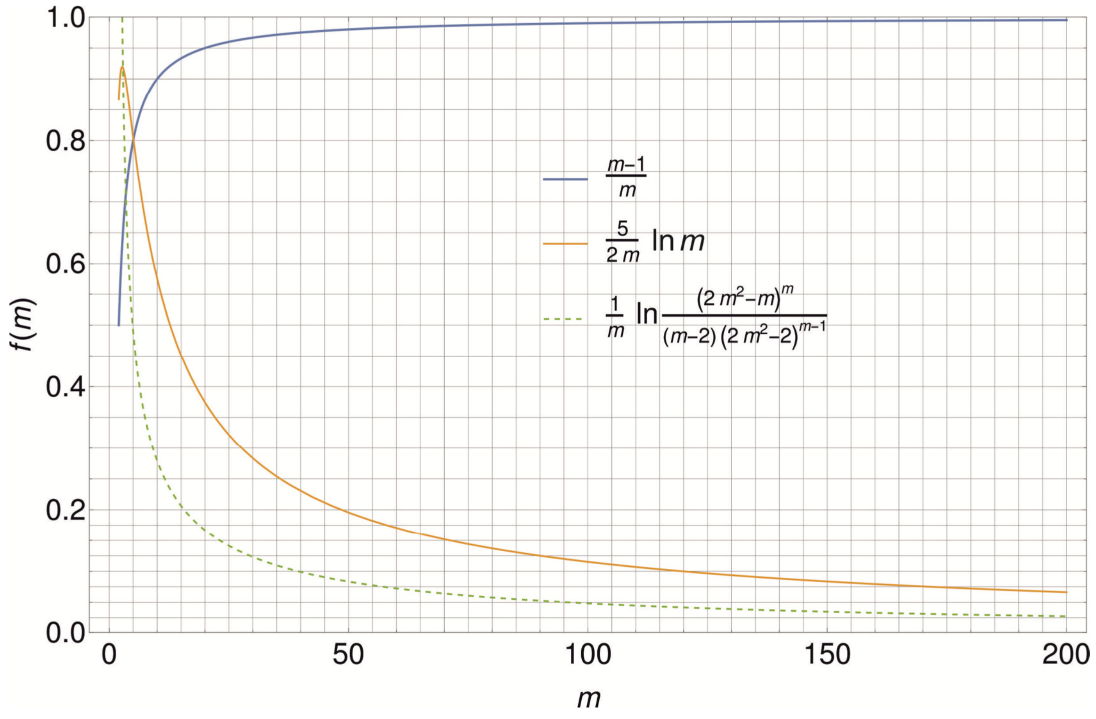

Figure S1. Illustration of the terms in Equation (S83) (blue and orange solid) and in Equation (S84) (orange dashed) that depend on the aggregation number m. Plots made with Mathematica 13.0.1.0 (© 1988-2022 Wolfram Research).

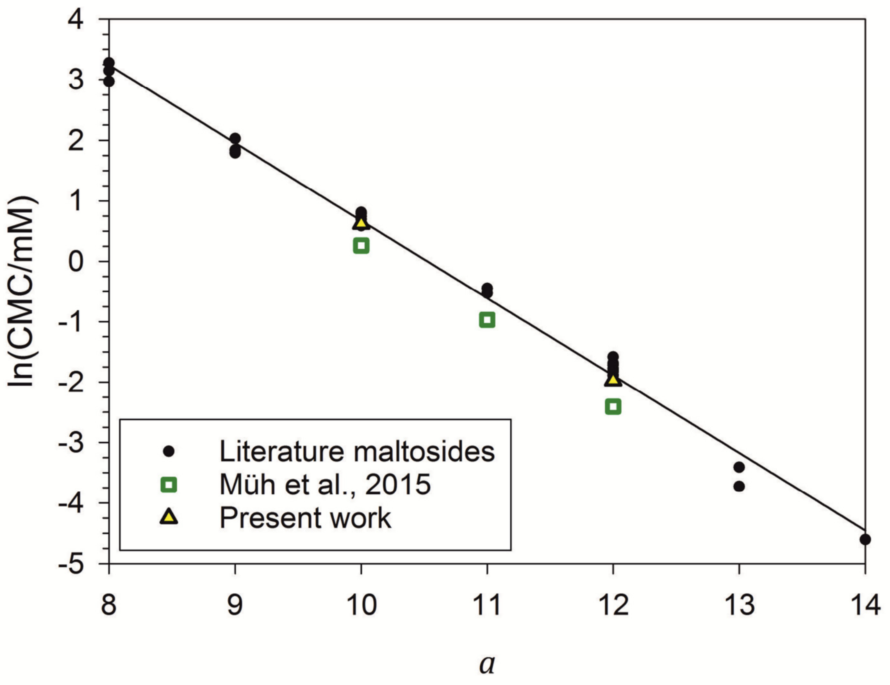

Figure S2. Dependence of $\ln(\mathrm{CMC/mM})$ of n-alkyl-ß-D-maltosides on the number a of carbon atoms in the alkyl tail for data compiled from the literature (black dots, cp. Table S3), our earlier work[10] (open green squares), and the present work (yellow triangles). The straight line results from a linear regression of the literature data with slope -1.28 ± 0.03 and intercept 13.5 ± 0.3 ($R^2 = 0.9931$). Plot and linear regression made with SigmaPlot 13 (© 2014 Systat Software Inc.).

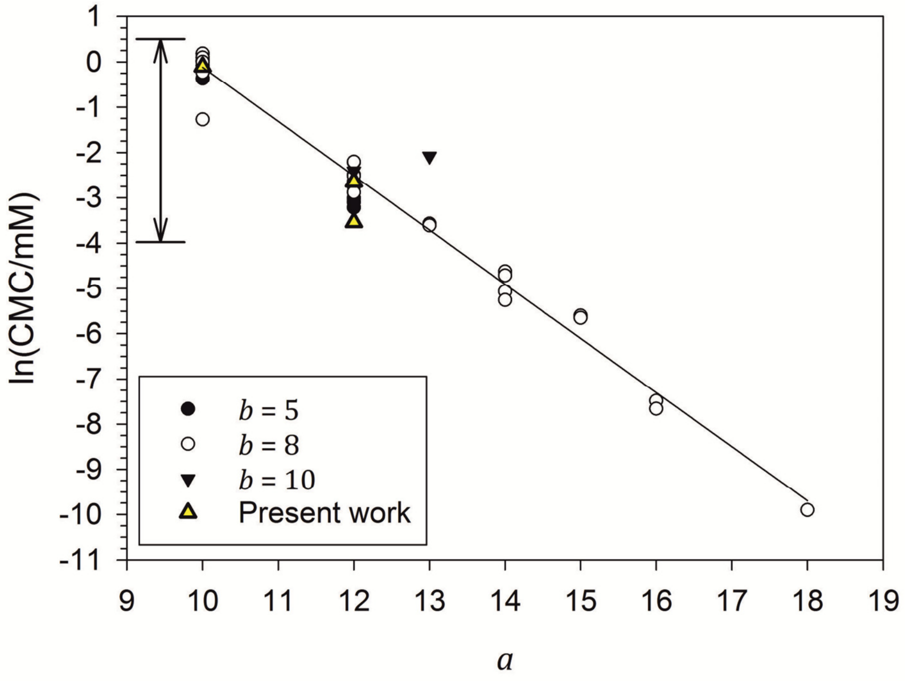

Figure S3. Dependence of $\ln(\mathrm{CMC/mM})$ of oligo(ethylene glycol) monoalkylether detergents C_aE_b on the number a of carbon atoms in the alkyl tail for data compiled from the literature (cp. Table S2) and the present work (yellow triangles). The straight line results from a linear regression of the literature data for $b = 8$ with slope -1.20 ± 0.03 and intercept 11.9 ± 0.4 ($R^2 = 0.9852$). The double arrow indicates the range of values for $\ln(\mathrm{CMC/mM})$ covered in Figure S4 for comparison. Plot and linear regression made with SigmaPlot 13 (© 2014 Systat Software Inc.).

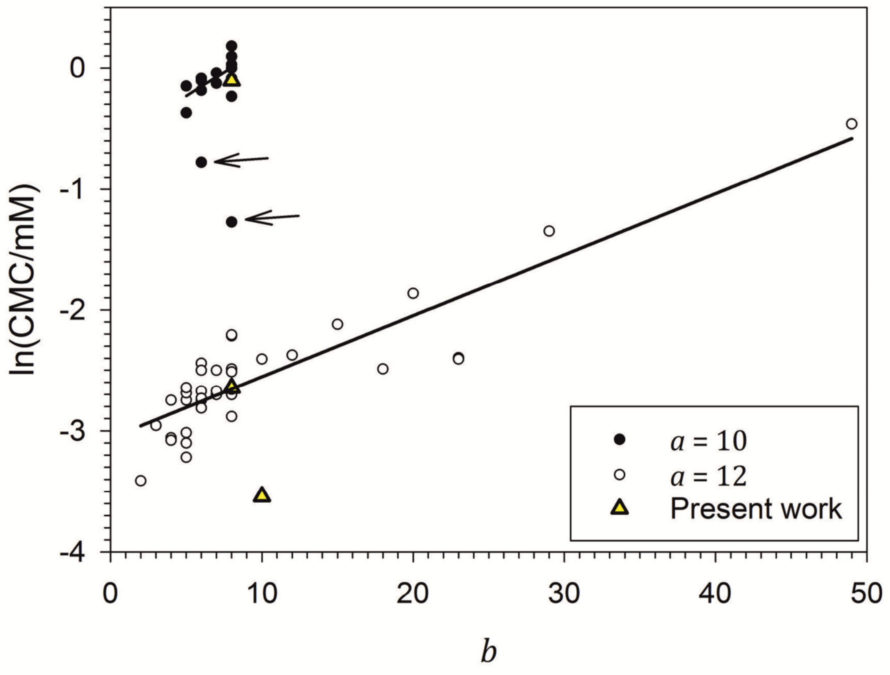

Figure S4. Dependence of $\ln(\mathrm{CMC/mM})$ of oligo(ethylene glycol) monoalkylether detergents C_aE_b on the number b of OE units in the head group for two different values of the number a of carbon atoms in the alkyl tail based on data compiled from the literature (cp. Table S2) and the present work (yellow triangles). The straight lines result from linear regression of the literature data with slope 0.079 ± 0.022 and intercept -0.63 ± 0.16 for $a = 10$ ($R^2 = 0.4672$) as well as slope 0.051 ± 0.005 and intercept -3.06 ± 0.07 for $a = 12$ ($R^2 = 0.7768$). The arrows indicate the data points from Casey and Reithmeier [44] for $a = 10$ that were excluded from the fit. Plot and linear regressions made with SigmaPlot 13 (© 2014 Systat Software Inc.).

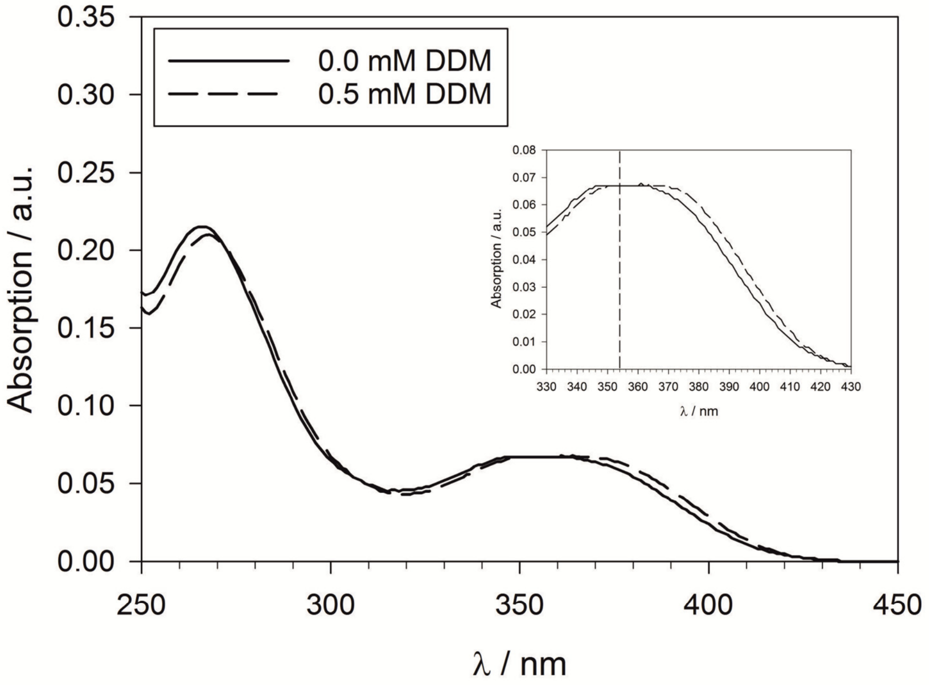

Figure S5. Absorption spectra of ANS in 100 mM PIPES (pH 7.0), 5 mM $CaCl_2$ at 19 ℃ in the absence (0.0 mM) and presence (0.5 mM) of DDM. The inset shows an expanded view of the region around the isosbestic point with the vertical dashed line indicating the excitation wavelength of 354 nm used in fluorescence experiments. Plots made with SigmaPlot 13 (© 2014 Systat Software Inc.).

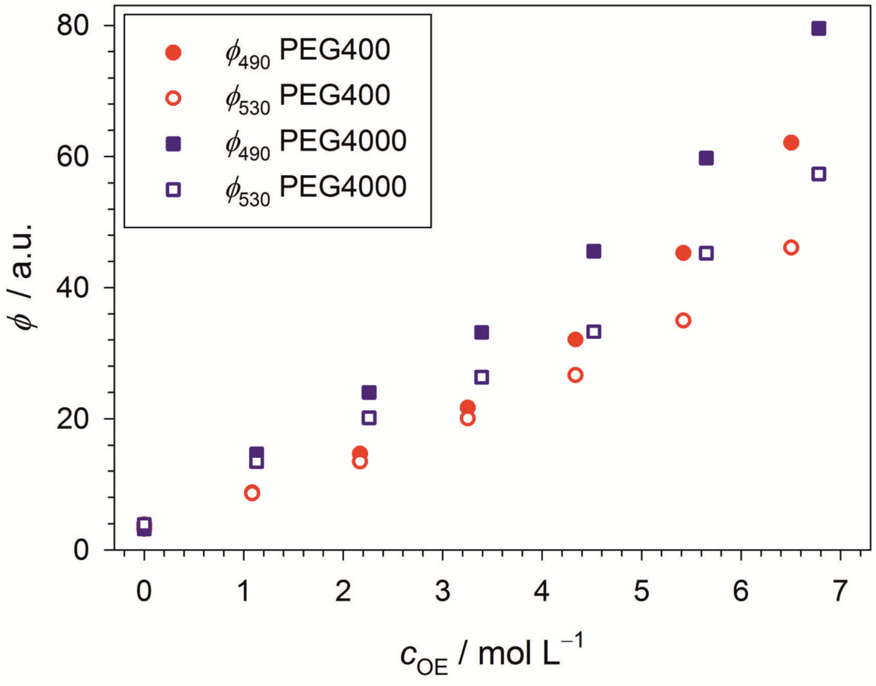

Figure S6. Dependence of the fluorescence intensities of ANS at the peak maxima (490 and 530 nm) in 100 mM PIPES (pH 7.0), 5 mM $CaCl_2$ at 19 ℃ in the absence of detergent (excitation wavelength: 354 nm) on the concentration of OE units c_{OE} of added PEG400 or PEG4000. Plot made with SigmaPlot 13 (© 2014 Systat Software Inc.).

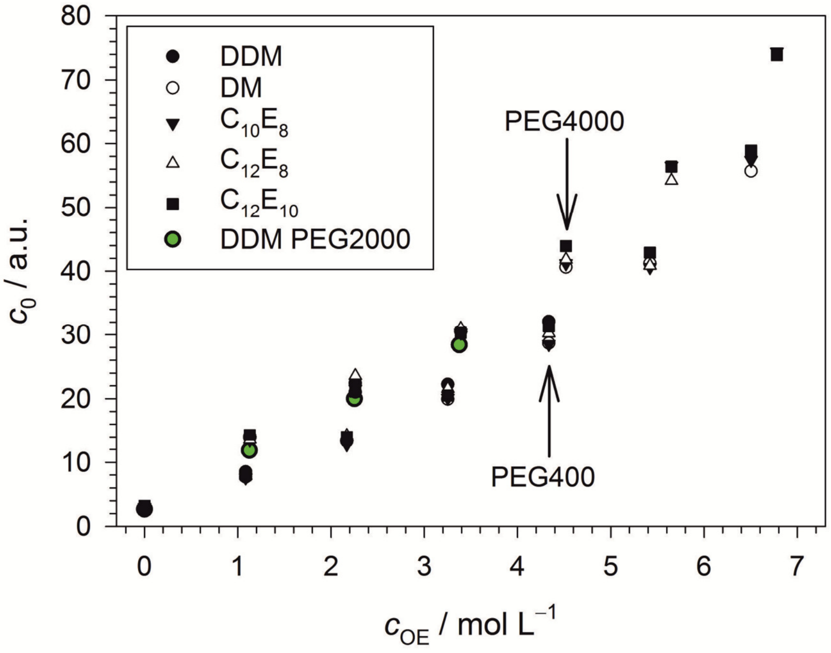

Figure S7. Dependence of the fit parameter c_0 (see Table S1) corresponding to ϕ_{480} below the CMC on the concentration of OE units c_{OE} of added PEG. Plot made with SigmaPlot 13 (© 2014 Systat Software Inc.).

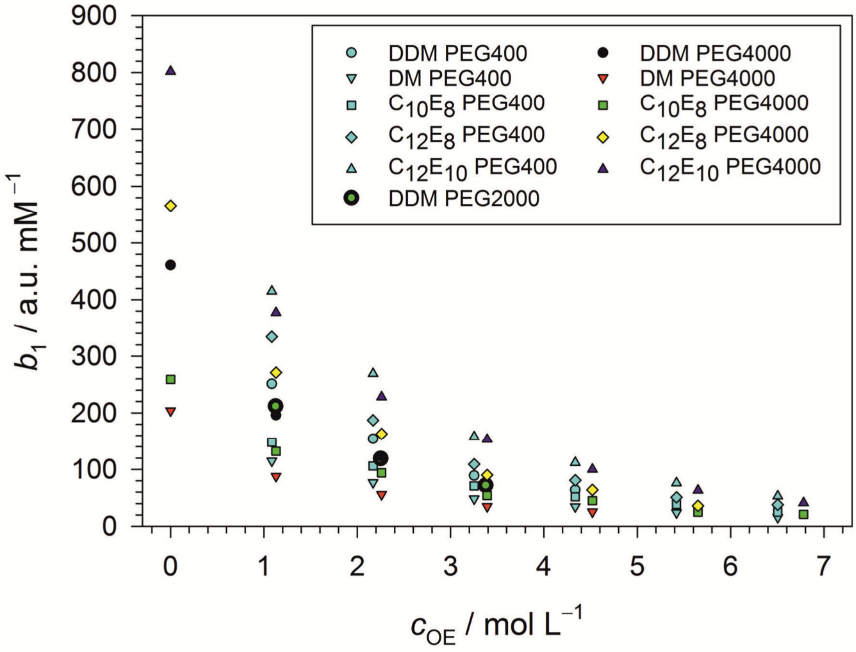

Figure S8. Dependence of the fit parameter b_1 (see Table S1) according to Equation (7) of the main text, corresponding to the slope of the initial rise of the ϕ_{480} titration curves above the CMC, on the concentration of OE units c_{OE} of added PEG. Plot made with SigmaPlot 13 (© 2014 Systat Software Inc.).

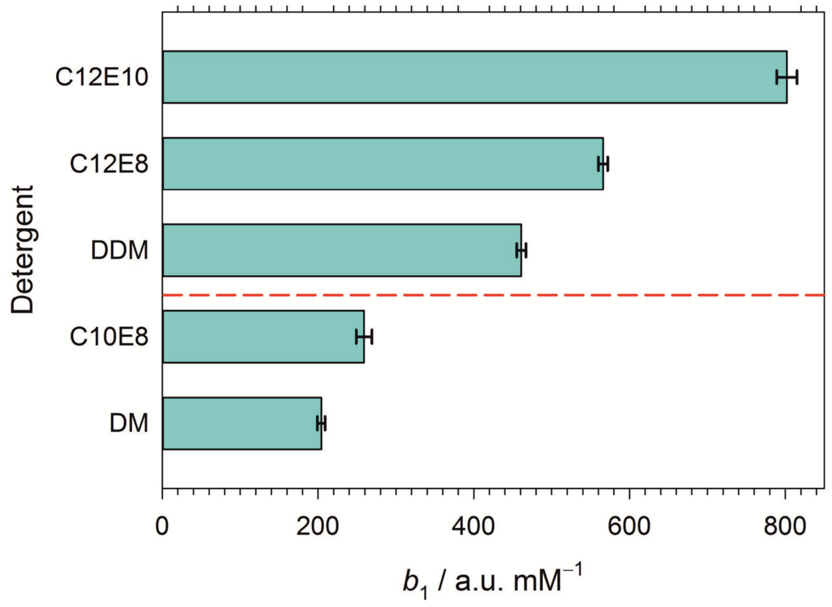

Figure S9. The fit parameter b_1 (see Table S1) according to Equation (7) of the main text, corresponding to the slope of the initial rise of the ϕ_{480} titration curves above the CMC, for the different detergents investigated at zero PEG concentration. The red dashed line separates the detergents with $a = 10$ (bottom) from those with $a = 12$ (top). Plot made with SigmaPlot 13 (© 2014 Systat Software Inc.).

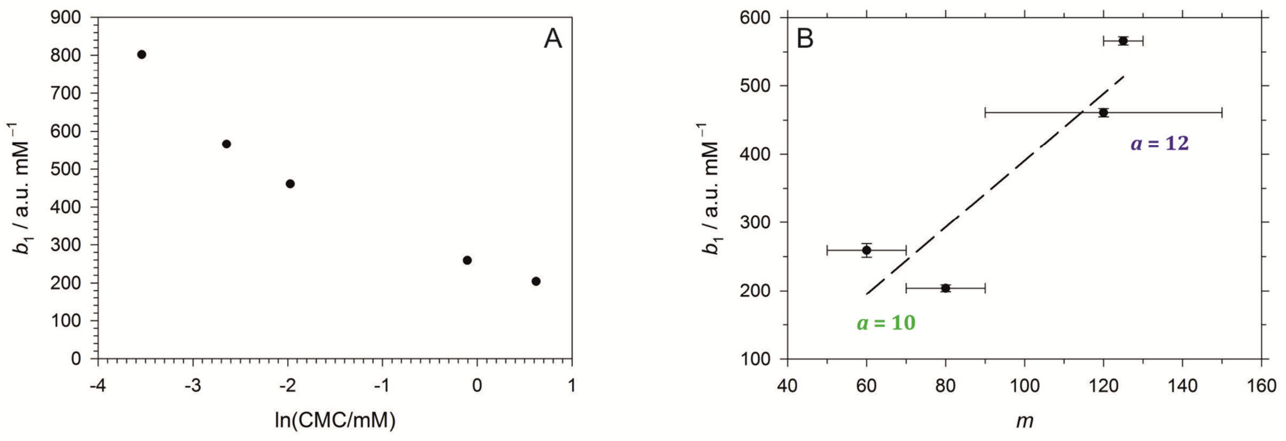

Figure S10. Correlation of the fit parameter b_1 (see Table S1) according to Equation (7) of the main text, corresponding to the slope of the initial rise of the ϕ_{480} titration curves above the CMC, at zero PEG concentration with the logarithm of the CMC (**A**) and the aggregation number m (**B**). Note that there is no data point for $C_{12}E_{10}$ in (**B**). The dashed straight line serves as a qualitative guide for the eye to indicate the rough correlation of b_1 with m. The error bars in (**B**) refer to the standard errors in Table S1 for b_1 and to a rough estimate from the data in Tables S2 and S3 for m. Plots made with SigmaPlot 13 (© 2014 Systat Software Inc.).

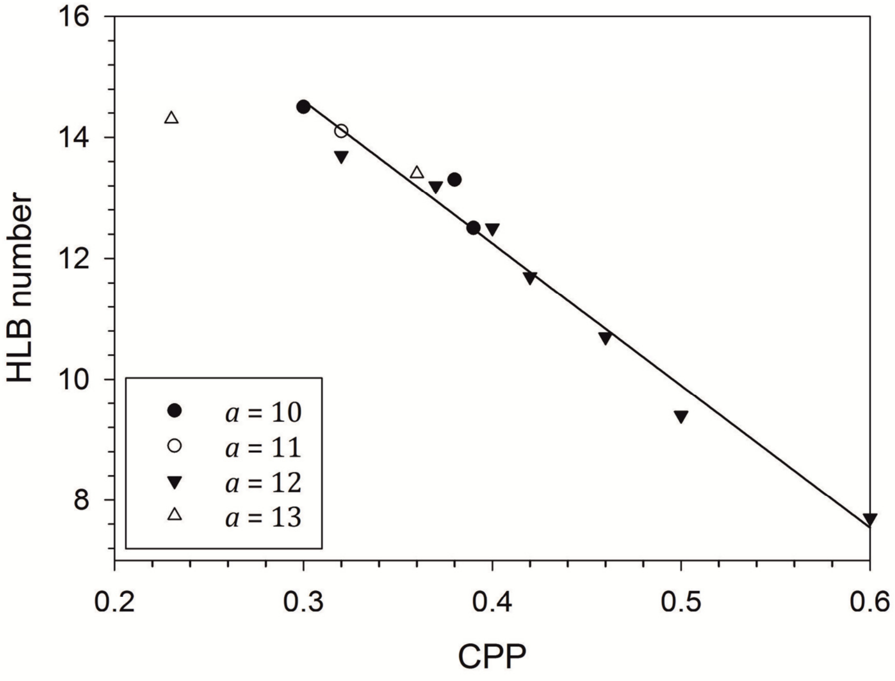

Figure S11. Correlation of the HLB number according to Equation (S87) with the critical packing parameter CPP according to Equation (S91) for oligo(ethylene glycol) monoalkylether detergents C_aE_b (cp. Table S2). The straight line results from linear regression of the data for $a = 10 - 12$ with slope -23.5 ± 1.2 and intercept 21.7 ± 0.5 ($R^2 = 0.9784$). Plot and linear regression made with SigmaPlot 13 (© 2014 Systat Software Inc.).

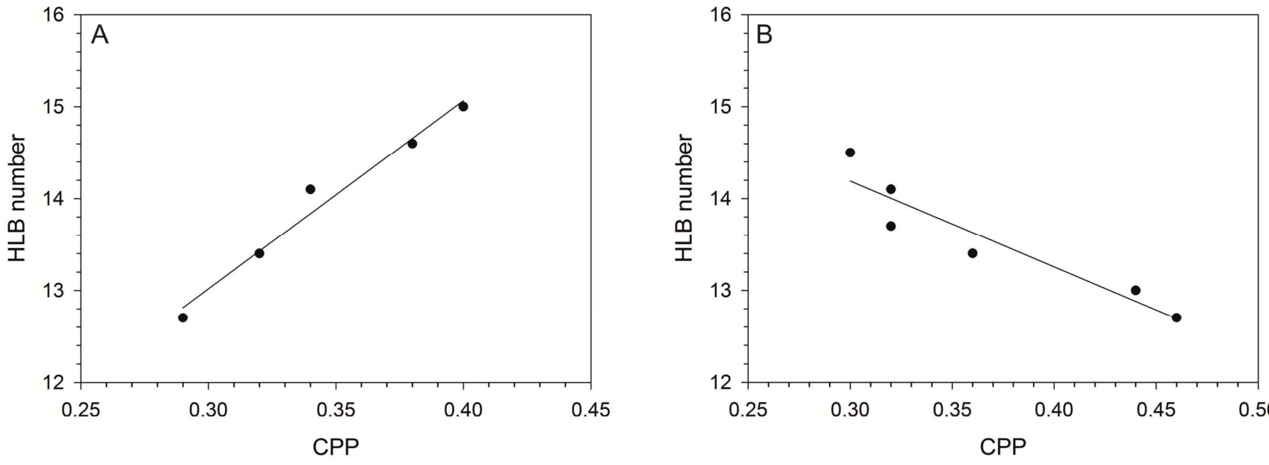

Figure S12. Correlation of the HLB number according to Equation (S87) with the critical packing parameter CPP according to Equation (S91). (**A**) *n*-Alkyl-ß-D-maltosides (cp. Table S3). The straight line results from linear regression with slope 20.5 ± 2.0 and intercept 6.9 ± 0.7 ($R^2 = 0.9736$). (**B**) Oligo(ethylene glycol) monoalkylether detergents C_aE_8 with eight OE units in the head group (cp. Table S2). The straight line results from linear regression with slope -9.4 ± 1.7 and intercept 17.0 ± 0.7 ($R^2 = 0.8839$). Plots and linear regressions made with SigmaPlot 13 (© 2014 Systat Software Inc.).

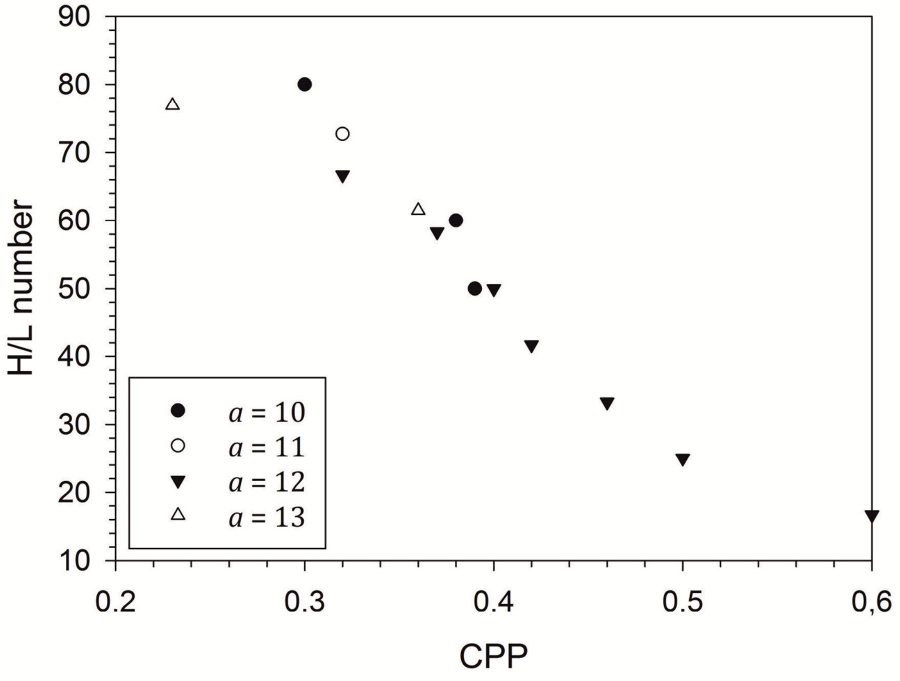

Figure S13. Correlation of the H/L number according to Equation (S89) with the critical packing parameter CPP according to Equation (S91) for oligo(ethylene glycol) monoalkylether detergents C_aE_b (cp. Table S2). Plot made with SigmaPlot 13 (© 2014 Systat Software Inc.).

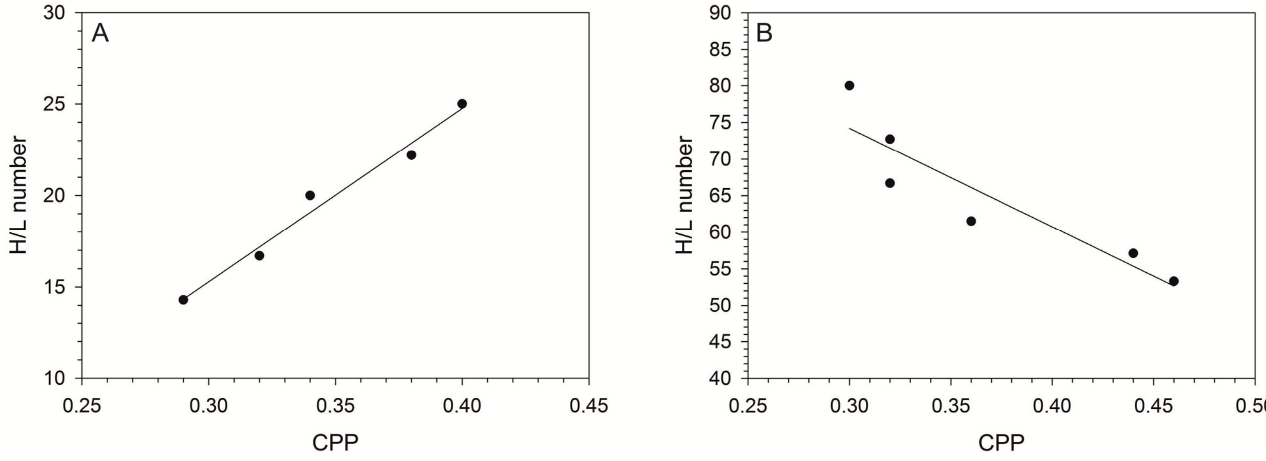

Figure S14. Correlation of the H/L number with the critical packing parameter CPP according to Equation (S91). (**A**) *n*-Alkyl-ß-D-maltosides (cp. Table S3, Equation (S90)). The straight line results from linear regression with slope 94.7 ± 8.2 and intercept -13.1 ± 2.9 ($R^2 = 0.9781$). (**B**) Oligo(ethylene glycol) monoalkylether detergents C_aE_8 with eight OE units in the head group (cp. Table S2, Equation (S89)). The straight line results from linear regression with slope -134.6 ± 30.7 and intercept 114.6 ± 11.2 ($R^2 = 0.8335$). Plots and linear regressions made with SigmaPlot 13 (© 2014 Systat Software Inc.).

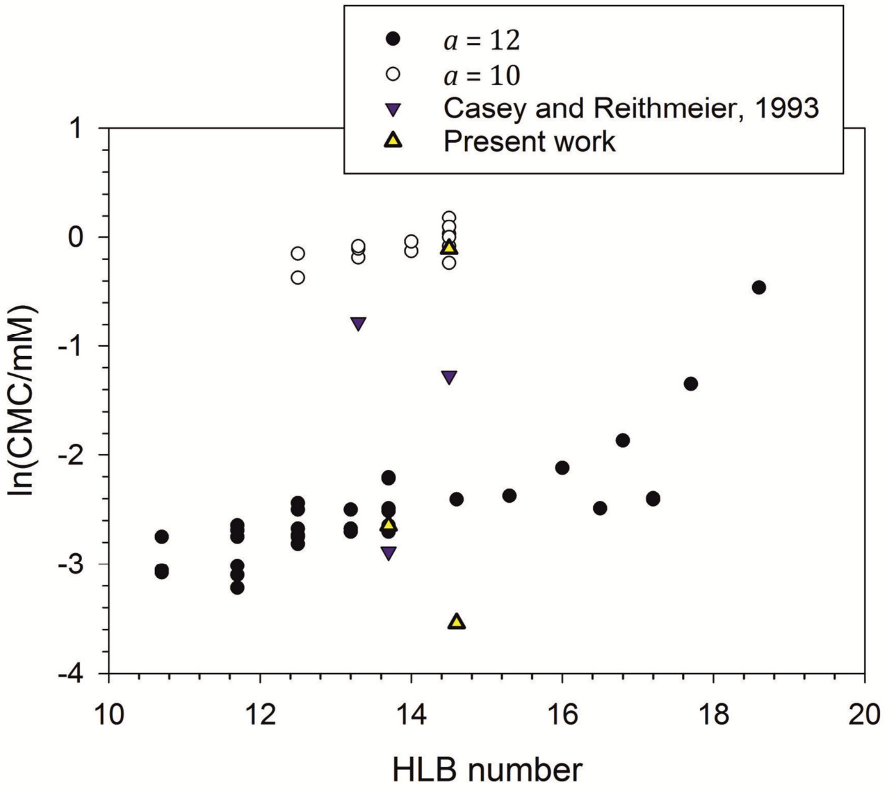

Figure S15. Correlation of $\ln(\mathrm{CMC/mM})$ of oligo(ethylene glycol) monoalkylether detergents $\mathrm{C}_a\mathrm{E}_b$ with the HLB number according to Equation (S87). Plot made with SigmaPlot 13 (© 2014 Systat Software Inc.).

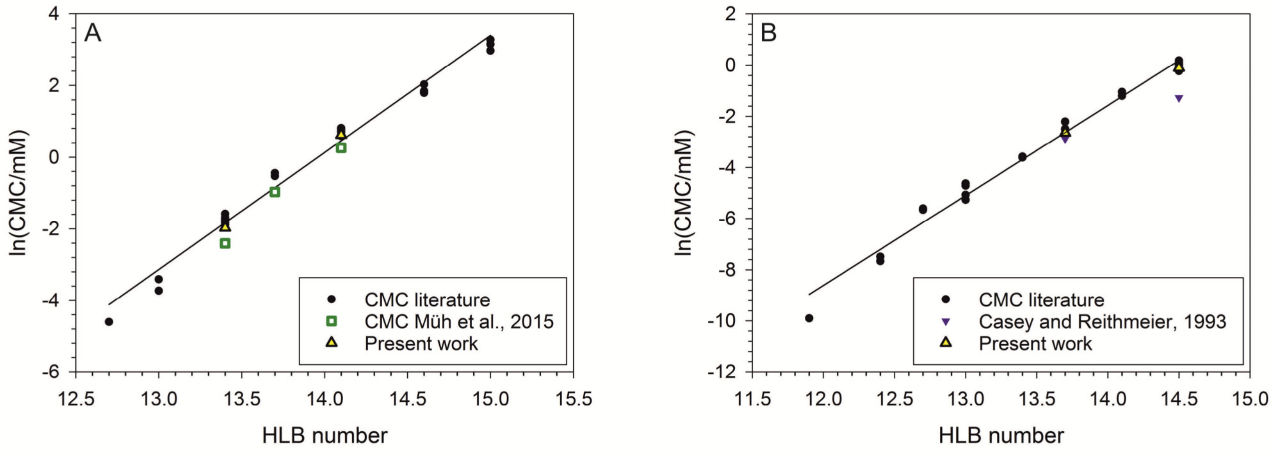

Figure S16. Correlation of $\ln(\mathrm{CMC/mM})$ with the HLB number defined in Equation (S87). (**A**) *n*-Alkyl-ß-D-maltosides (cp. Table S3). The straight line results from linear regression of the literature data corresponding to Equation (S94) with $C_2 = 3.27 \pm 0.09$ and $C_1 = -45.6 \pm 1.3$ ($R^2 = 0.9833$). (**B**) Oligo(ethylene glycol) monoalkylether detergents $\mathrm{C}_a\mathrm{E}_8$ with eight OE units in the head group (cp. Table S2). The straight line results from linear regression of the literature data corresponding to Equation (S94) with $C_2 = 3.52 \pm 0.09$ and $C_1 = -50.8 \pm 1.2$ ($R^2 = 0.9854$). Plots and linear regressions made with SigmaPlot 13 (© 2014 Systat Software Inc.).

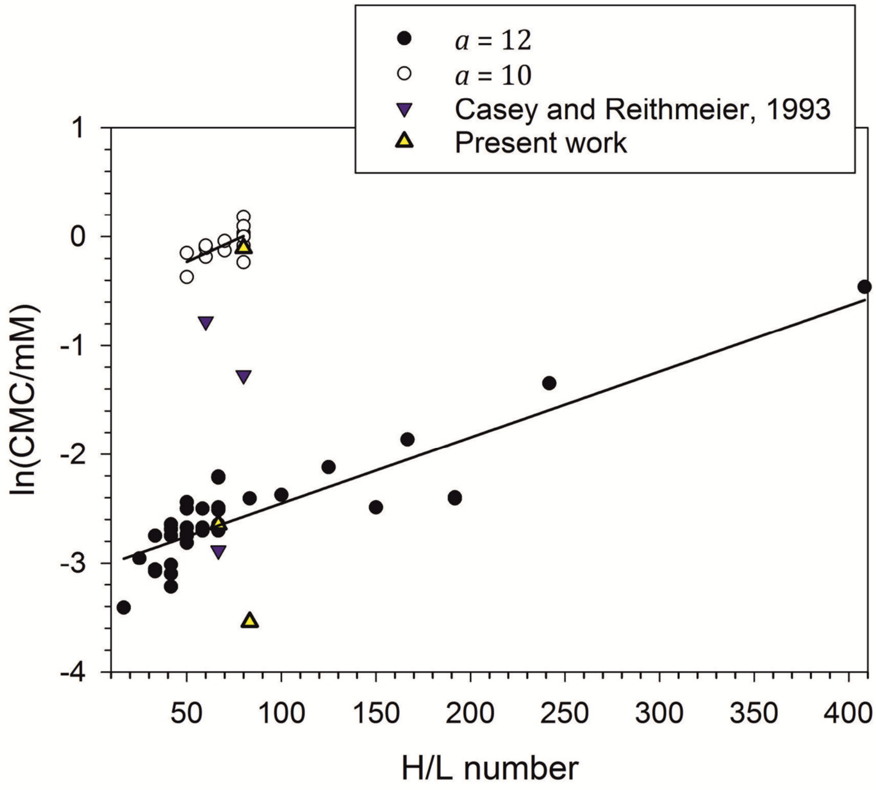

Figure S17. Correlation of $\ln(\mathrm{CMC/mM})$ of oligo(ethylene glycol) monoalkylether detergents $\mathrm{C}_a\mathrm{E}_b$ with the H/L number according to Equation (S89). The straight lines result from linear regression of the literature data (excluding those from Casey and Reithmeier[44] for $a = 10$) with slope 0.008 ± 0.003 and intercept -0.63 ± 0.16 for $a = 10$ ($R^2 = 0.4672$) as well as slope 0.0061 ± 0.0006 and intercept -3.06 ± 0.07 for $a = 12$ ($R^2 = 0.7768$). Plot and linear regressions made with SigmaPlot 13 (© 2014 Systat Software Inc.).

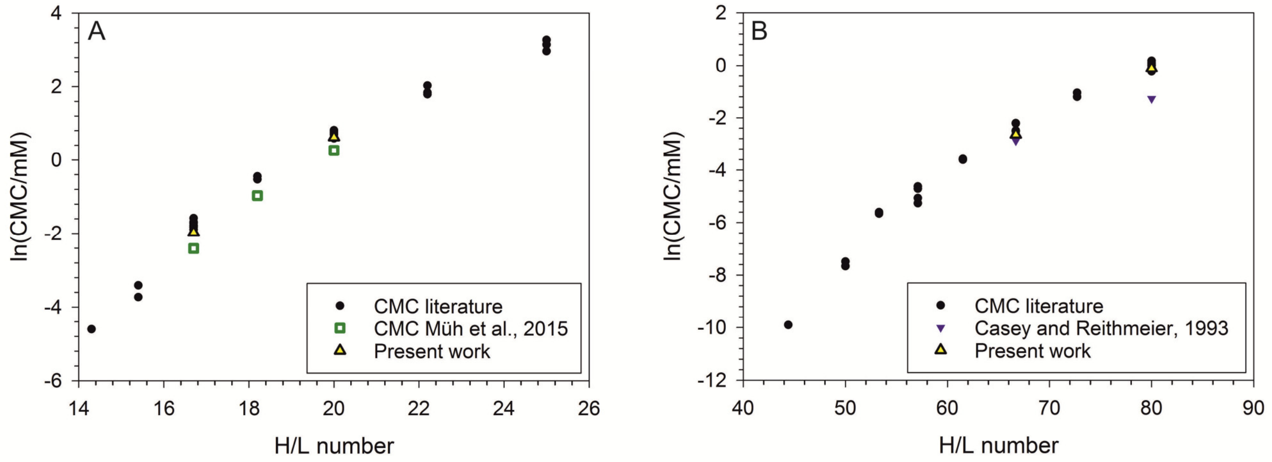

Figure S18. Correlation of $\ln(\mathrm{CMC/mM})$ with the H/L number. **A**) *n*-Alkyl-ß-D-maltosides (cp. Table S3, Equation (S90)). (**B**) Oligo(ethylene glycol) monoalkylether detergents $\mathrm{C}_a\mathrm{E}_8$ with eight OE units in the head group (cp. Table S2, Equation (S89)). Plots made with SigmaPlot 13 (© 2014 Systat Software Inc.).

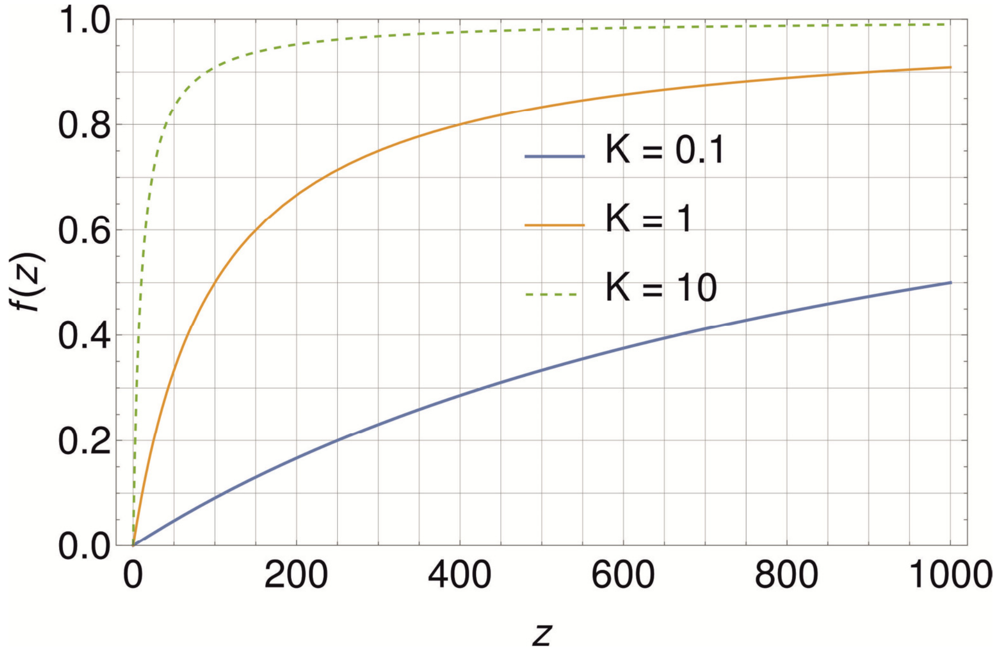

Figure S19. Plots of the function $f(z) = zK/(100 + zK)$ for different values of K. Plots made with Mathematica 13.0.1.0 (© 1988-2022 Wolfram Research).

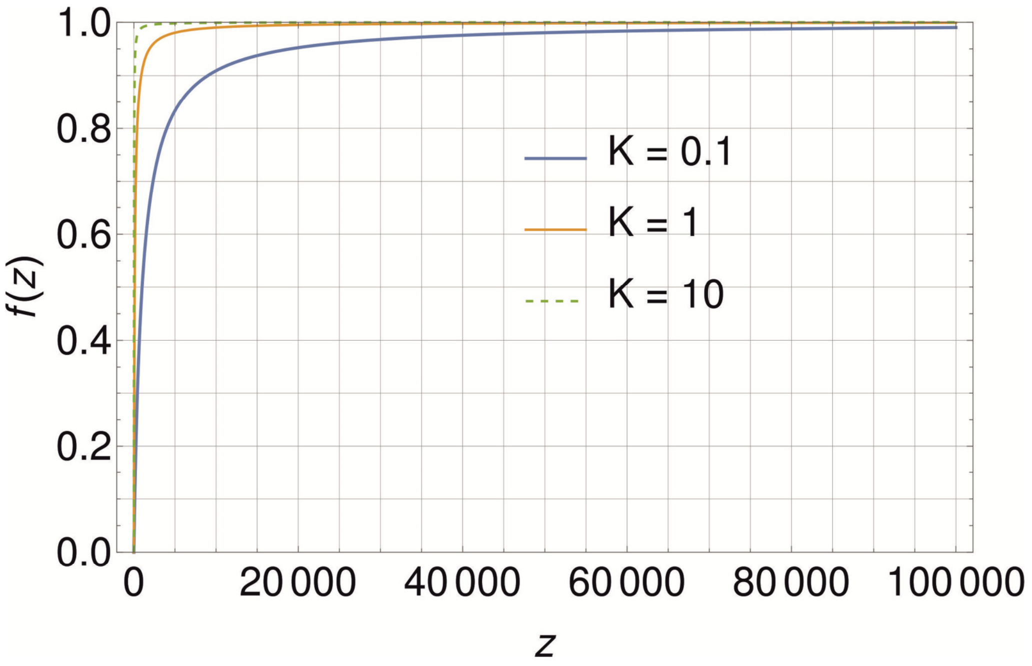

Figure S20. Same as in Figure S19, but with an extended scale for z. Plots made with Mathematica 13.0.1.0 (© 1988-2022 Wolfram Research).

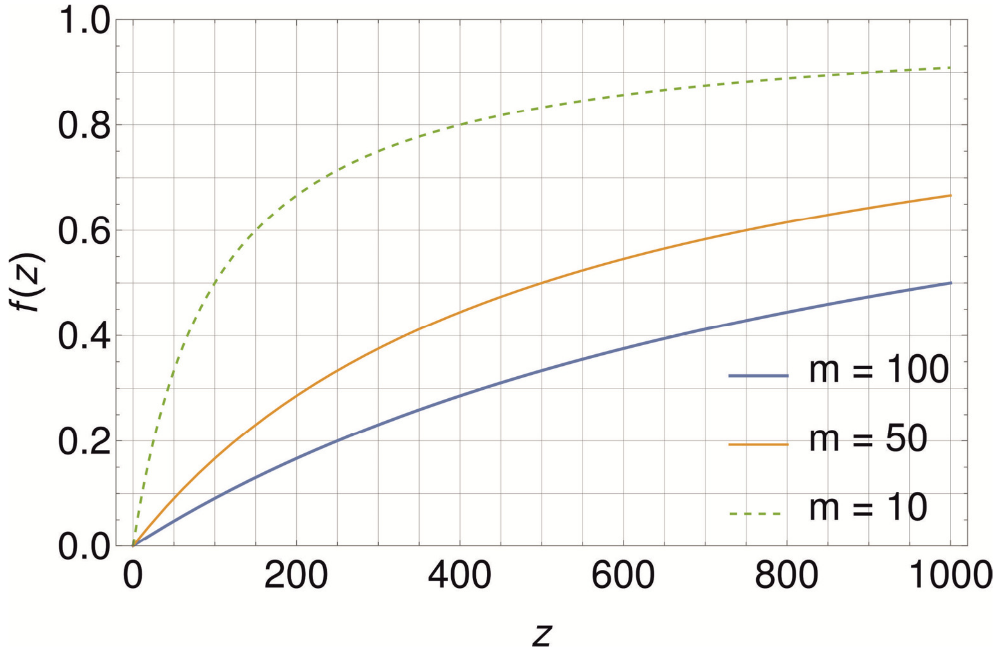

Figure S21. Plots of the function $f(z) = 0.1\ z/(m + 0.1\ z)$ for different values of m. Note that the curve for $m = 100$ is the same as in Figure S19 for $K = 0.1$. Plots made with Mathematica 13.0.1.0 (© 1988-2022 Wolfram Research).

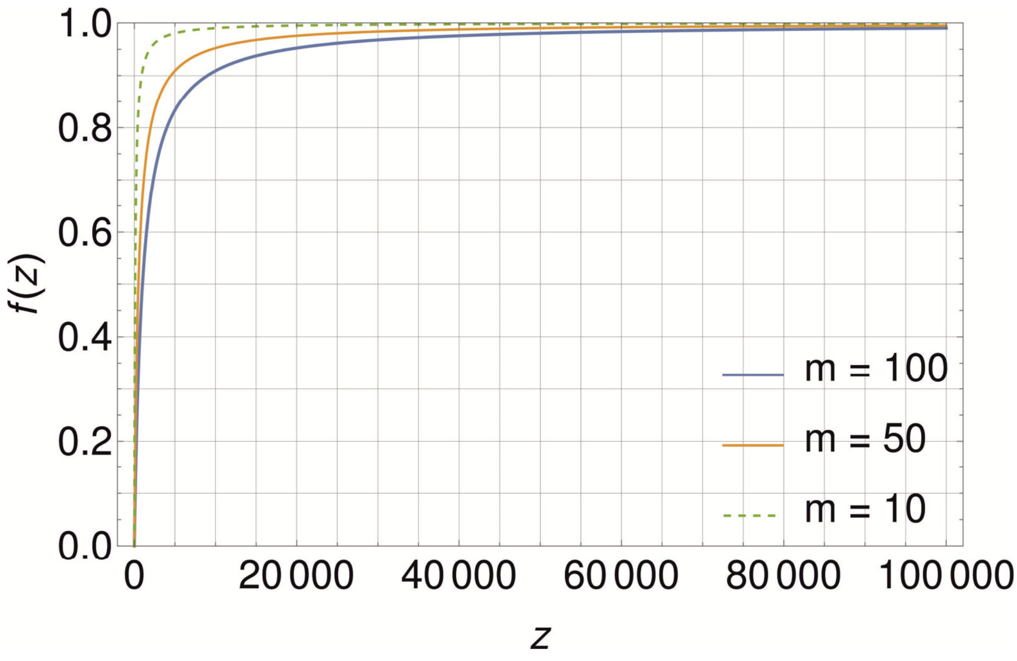

Figure S22. Same as in Figure S21, but with an extended scale for z. Plots made with Mathematica 13.0.1.0 (© 1988-2022 Wolfram Research).

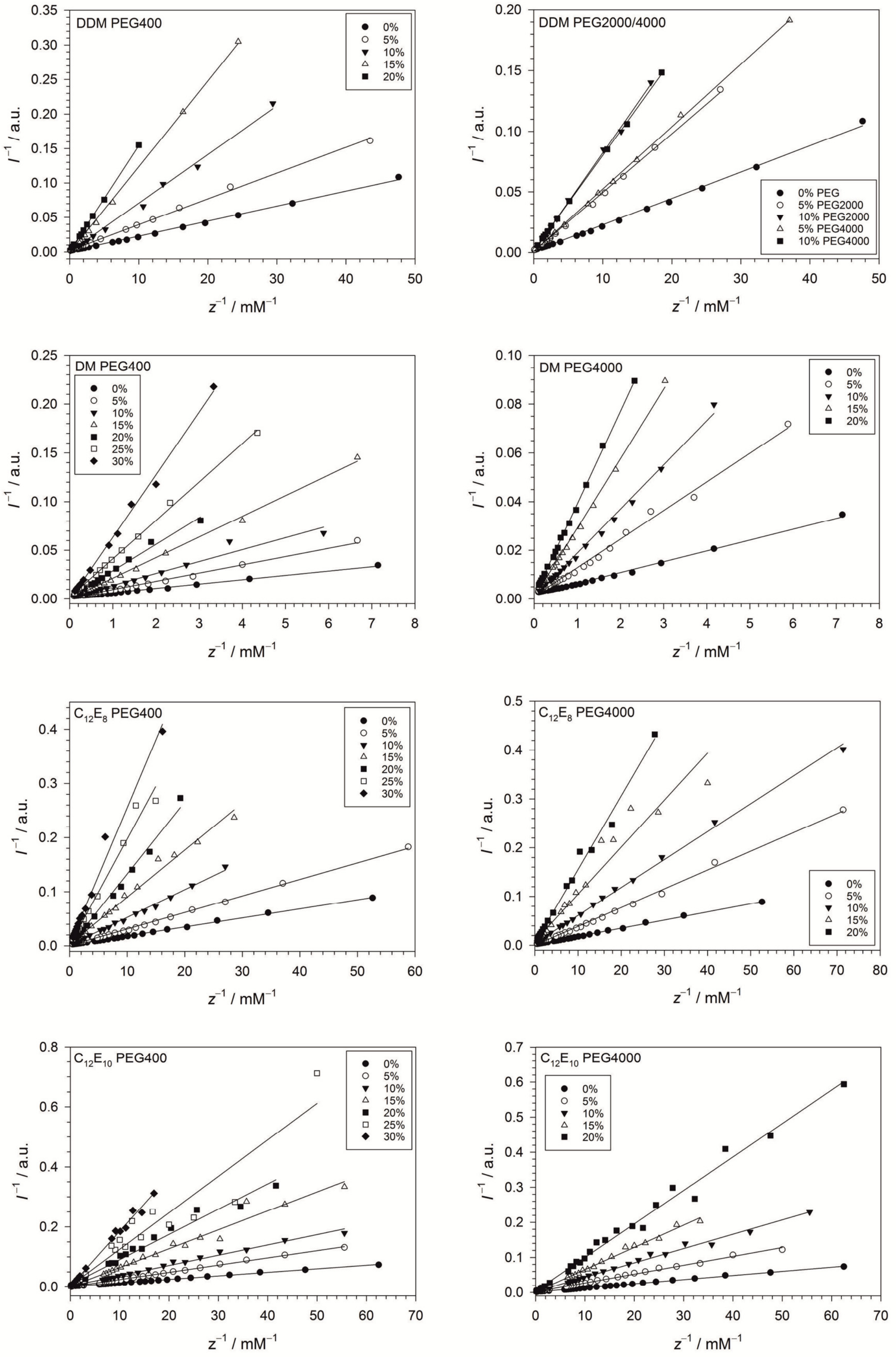

Figure S23. Double-reciprocal plot of $I(z)$ data points far above the CMC for various detergents and for different concentrations of PEG (in %(w/v), see legend). Plots made with SigmaPlot 13 (© 2014 Systat Software Inc.).

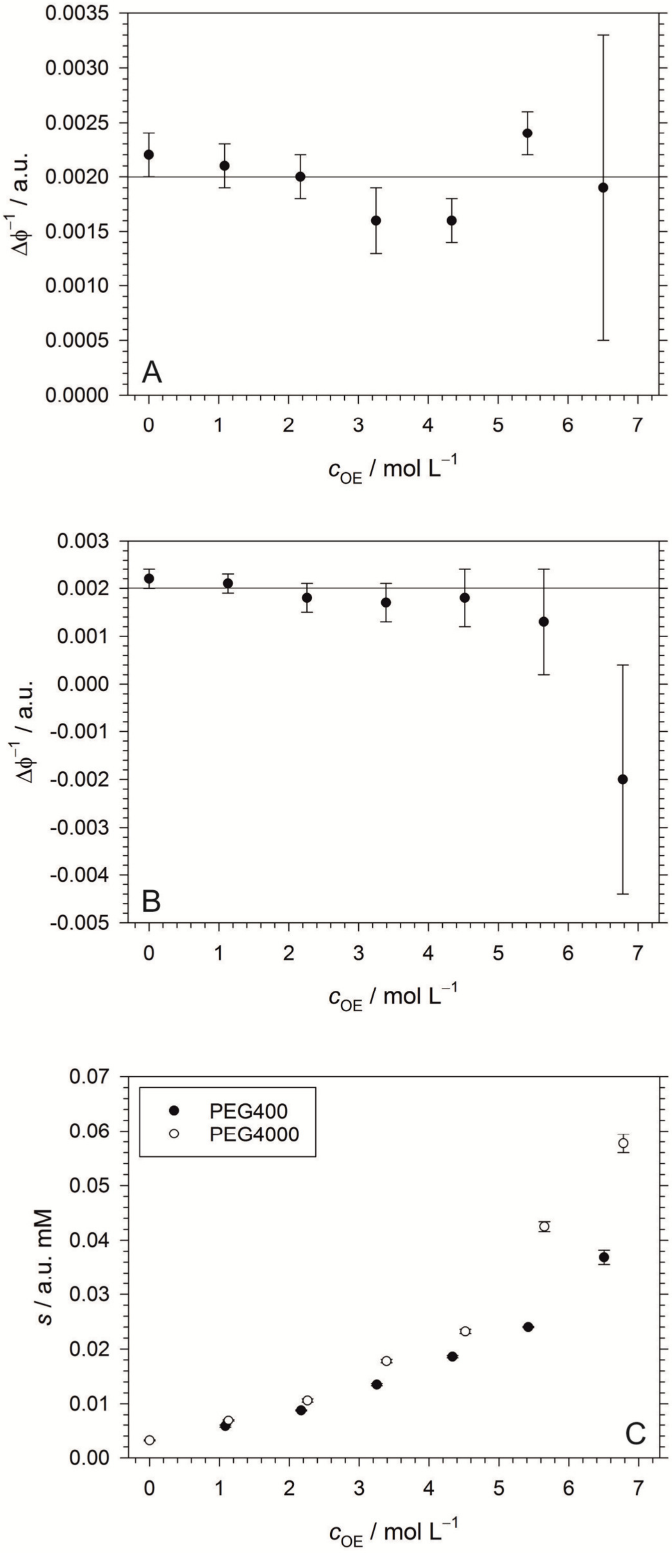

Figure S24. Values of $\Delta\phi^{-1}$ according to Equation (S107) and of s according to Equation (S108) obtained from linear regressions of the double-reciprocal plots for $C_{10}E_8$ shown in Figure 10B,C of the main text (see also Table S4) as a function of the molar concentration of OE units of the added PEG. (**A**) $\Delta\phi^{-1}$ for PEG400. (**B**) $\Delta\phi^{-1}$ for PEG4000. (**C**) s for PEG400 and PEG4000. Plots made with SigmaPlot 13 (© 2014 Systat Software Inc.).

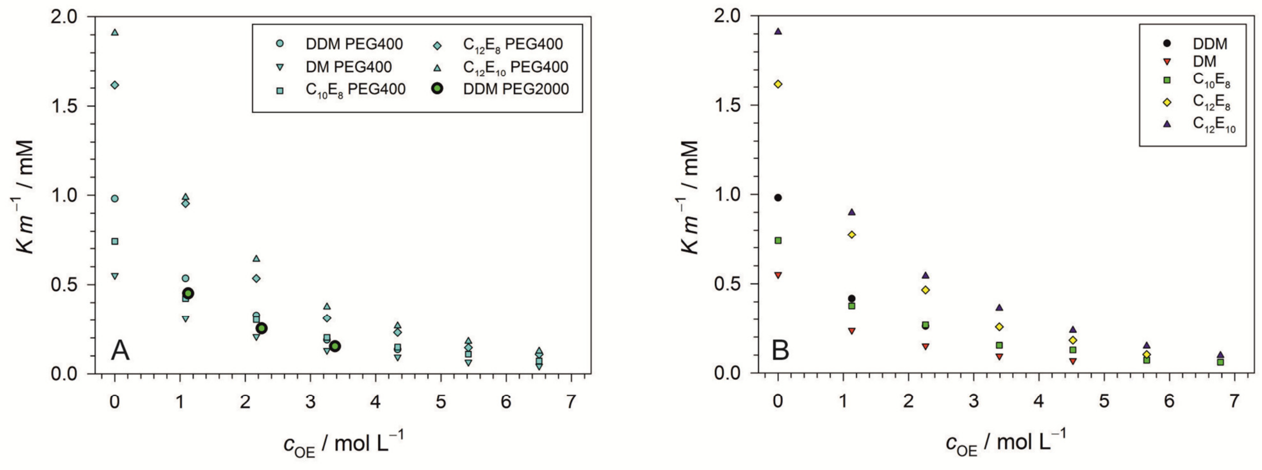

Figure S25. (**A**) Values of K/m obtained from Equation (13) of the main text to characterize the binding of ANS to the micelles of the various detergents as a function of the molar concentration of OE units of the added PEG for PEG400 and PEG2000. (**B**) Same as in (**A**), but for PEG4000. Plots made with SigmaPlot 13 (© 2014 Systat Software Inc.).

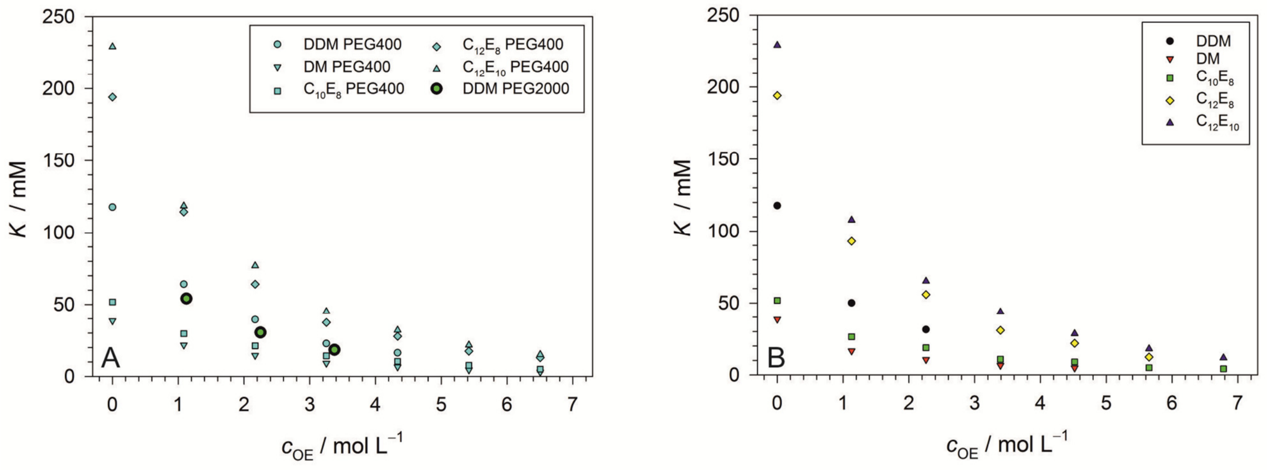

Figure S26. (**A**) Values of K obtained from Equation (13) of the main text to characterize the binding of ANS to the micelles of the various detergents as a function of the molar concentration of OE units of the added PEG for PEG400 and PEG2000. (**B**) Same as in (**A**), but for PEG4000. We used $m = 70$ for detergents with $a = 10$ and $m = 120$ for $a = 12$. Plots made with SigmaPlot 13 (© 2014 Systat Software Inc.).

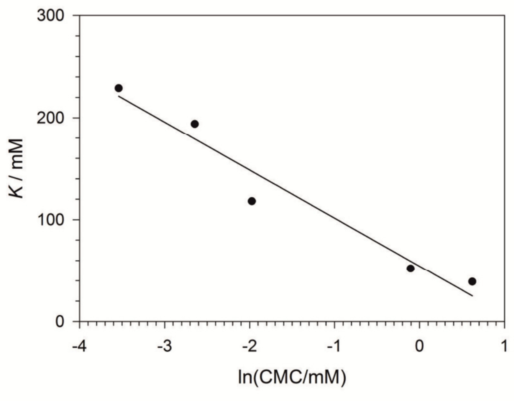

Figure S27. Correlation of the binding constant K with $\ln(\mathrm{CMC/mM})$ at zero PEG concentration. Plot made with SigmaPlot 13 (© 2014 Systat Software Inc.).

Supplementary References

[1] H. B. Callen, *Thermodynamics and an Introduction to Thermostatistics*, Wiley, New York, **1985**.

[2] M. E. Tuckerman, *Statistical Mechanics: Theory and Molecular Simulation*, Oxford University Press, New York, **2010**.

[3] A. Ben-Naim, *J. Phys. Chem.* **1978**, 82, 792-803.

[4] G. Némethy, H. A. Scheraga, *J. Chem. Phys.* **1962**, 36, 3401-3417.

[5] T. L. Hill, *An Introduction to Statistical Thermodynamics*, Dover, New York, **1960, 1986**.

[6] A. Ben-Naim, *Molecular Theory of Solutions*, Oxford University Press, New York, **2006**.

[7] A. Hobson, *Concepts of Statistical Mechanics*, Gordon and Breach Science Publishers, New York, **1971**.

[8] A. Bothe, A. Zouni, F. Müh, *RSC Adv.* **2023**, 13, 9387-9401.

[9] R. Nagarajan, E. Ruckenstein, *Langmuir* **1991**, 7, 2934-2969.

[10] F. Müh, D. DiFiore, A. Zouni, *Phys. Chem. Chem. Phys.* **2015**, 17, 11678-11691.

[11] F. Müh, A. Bothe, A. Zouni, *Photosynth. Res.* **2024**, 162, 273-289.

[12] F. Müh, *Colloids Interfaces* **2024**, 8, 60.

[13] J. N. Phillips, *Trans. Faraday Soc.* **1955**, 51, 561-569.

[14] W. Al-Soufi, L. Pineiro, M. Novo, *J. Colloid Interface Sci.* **2012**, 370, 102-110.

[15] J. M. Corkill, J. F. Goodman, S. P. Harrold, *Trans. Faraday Soc.* **1964**, 60, 202-207.

[16] W. C. Griffin, *J. Soc. Cosmet. Chem.* **1949**, 1, 311-326.

[17] R. G. Laughlin, *The aqueous phase behavior of surfactants*, Academic Press, London, **1994**.

[18] K. Holmberg, B. Jönsson, B. Kronberg, B. Lindman, *Surfactants and Polymers in Aqueous Solution*, Wiley, Chichester, **2003**.

[19] D. E. Evans, H. Wennerström, *The Colloidal Domain*, Wiley-VCH, New York, **1999**.

[20] B. Aveyard, *Surfactants. In Solution, at Interfaces and in Colloidal Dispersions*, Oxford University Press, Oxford, **2019**.

[21] P. Becher, *J. Dispersion Sci. Technol.* **1984**, 5, 81-96.

[22] J. T. Davies, *Prog. Int. Congr. Surface Activity, 2nd, London* **1957**, 1, 426-438.

[23] R. G. Laughlin, *J. Soc. Cosmet. Chem.* **1981**, 32, 371-392.

[24] K. Shinoda, S. Friberg, *Emulsions and Solubilization*, Wiley-Interscience, New York, **1986**.

[25] C. D. Moore, M. Bell, *Soap, Perfum. Cosmet.* **1956**, 29, 893.

[26] J. N. Israelachvili, D. J. Mitchell, B. W. Ninham, *J. Chem. Soc. Faraday Trans. 2* **1976**, 72, 1525-1568.

[27] C. Rodríguez-Abreu, *J. Surfact. Deterg.* **2019**, 22, 1001-1010.

[28] J. N. Israelachvili, *Intermolecular and Surface Forces*, Academic Press, Elsevier, Amsterdam, **2011**.

[29] Z. C. Li, G. Y. Chen, L. Q. Chen, Y. H. Zhang, Z. Y. Dai, *J. Surfact. Deterg.* **2019**, 22, 731-742.

[30] J. Lipfert, L. Columbus, V. B. Chu, S. A. Lesley, S. Doniach, *J. Phys. Chem. B* **2007**, 111, 12427-12438.

[31] R. C. Oliver, J. Lipfert, D. A. Fox, R. H. Lo, S. Doniach, L. Columbus, *PloS ONE* **2013**, 8, e62488.

[32] I. J. Lin, P. Somasundaran, *J. Colloid Interface Sci.* **1971**, 37, 731-743.

[33] I. J. Lin, J. P. Friend, Y. Zimmels, *J. Colloid Interface Sci.* **1973**, 45, 378-385.

[34] E. B. Abuin, E. A. Lissi, A. Aspée, F. D. Gonzalez, J. M. Varas, *J. Colloid Interface Sci.* **1997**, 186, 332-338.

[35] E. De Vendittis, G. Palumbo, G. Parlato, V. Bocchini, *Anal. Biochem.* **1981**, 115, 278-286.

[36] J. Van Ede, J. R. J. Nijmeijer, S. Welling-Wester, C. Örvell, G. W. Welling, *J. Chromatogr.* **1989**, 476, 319-327.

[37] W. L. Hinze, E. Pramauro, *Crit. Rev. Anal. Chem.* **1993**, 24, 133-177.

[38] K. Meguro, Y. Takasawa, N. Kawahashi, Y. Tabata, M. Ueno, *J. Colloid Interface Sci.* **1981**, 83, 50-56.

[39] M. J. Rosen, A. W. Cohen, M. Dahanayake, X. Y. Hua, *J. Phys. Chem.* **1982**, 86, 541-545.

[40] N. Nishikido, Y. Moroi, R. Matuura, *Bull. Chem. Soc. Jpn.* **1975**, 48, 1387-1390.

[41] N. Funasaki, H. S. Shim, S. Hada, *J. Phys. Chem.* **1992**, 96, 1998-2006.

[42] H. Hidaka, J. C. Zhao, K. Kitamura, K. Nohara, N. Serpone, E. Pelizzetti, *J. Photochem. Photobiol. A* **1992**, 64, 103-113.

[43] Y. Moroi, R. Matuura, *Bull. Chem. Soc. Jpn.* **1988**, 61, 333-339.

[44] J. R. Casey, R. A. F. Reithmeier, *Biochemistry* **1993**, 32, 1172-1179.

[45] C. Tanford, J. A. Reynolds, *Biochim. Biophys. Acta* **1976**, 457, 133-170.

[46] N. Funasaki, H. S. Shim, S. Hada, *J. Chem. Soc. Faraday Trans. I* **1991**, 87, 957-961.

[47] M. F. Borgerding, W. L. Hinze, *Anal. Chem.* **1985**, 57, 2183-2190.

[48] P. Liljekvist, B. Kronberg, *J. Colloid Interface Sci.* **2000**, 222, 159-164.

[49] C. J. Drummond, G. G. Warr, F. Grieser, B. W. Ninham, D. F. Evans, *J. Phys. Chem.* **1985**, 89, 2103-2109.

[50] P. Kroll, J. Benke, S. Enders, C. Brandenbusch, G. Sadowski, *ACS Omega* **2022**, 7, 7057-7065.

[51] R. R. Balmbra, J. S. Clunie, J. F. Goodman, J. M. Corkill, *Trans. Faraday Soc.* **1964**, 60, 979-985.

[52] K. V. Schubert, R. Strey, M. Kahlweit, *J. Colloid Interface Sci.* **1991**, 141, 21-29.

[53] M. Donbrow, Z. A. Jan, *J. Pharm. Pharmacol.* **1963**, 15, 825-830.

[54] M. Ueno, Y. Takasawa, H. Miyashige, Y. Tabata, K. Meguro, *Colloid Polymer Sci.* **1981**, 259, 761-766.

[55] T. M. Herrington, S. S. Sahi, *J. Colloid Interface Sci.* **1988**, 121, 107-120.

[56] W. Brown, Z. Pu, R. Rymden, *J. Phys. Chem.* **1988**, 92, 6086-6094.

[57] H. Alpes, K. Allmann, H. Plattner, J. Reichert, R. Riek, S. Schulz, *Biochim. Biophys. Acta Biomembr.* **1986**, 862, 294-302.

[58] W. J. De Grip, P. H. M. Bovee-Geurts, *Chem. Phys. Lipids* **1979**, 23, 321-335.

[59] A. D. Tsamaloukas, A. Beck, H. Heerklotz, *Langmuir* **2009**, 25, 4393-4401.

[60] M. Aoudia, R. Zana, *J. Colloid Interface Sci.* **1998**, 206, 158-167.

[61] R. Aveyard, B. P. Binks, J. Chen, J. Esquena, P. D. I. Fletcher, R. Buscall, S. Davies, *Langmuir* **1998**, 14, 4699-4709.

[62] P. Strop, A. T. Brunger, *Protein Sci.* **2005**, 14, 2207-2211.

[63] T. VanAken, S. Foxall-VanAken, S. Castleman, S. Ferguson-Miller, *Methods Enzymol.* **1986**, 125, 27-35.

[64] C. Dupuy, X. Auvray, C. Petipas, *Langmuir* **1997**, 13, 3965-3967.

[65] P. J. Tummino, A. Gafni, *Biophys J.* **1993**, 64, 1580-1587.

[66] P. Rosevear, T. VanAken, J. Baxter, S. Ferguson-Miller, *Biochemistry* **1980**, 19, 4108-4115.

[67] G. G. Warr, C. J. Drummond, F. Grieser, B. W. Ninham, D. F. Evans, *J Phys Chem* **1986**, 90, 4581-4586.

[68] E. R. S. Kunji, M. Harding, P. J. G. Butler, P. Akamine, *Methods* **2008**, 46, 62-72.

[69] T. Jumpertz, B. Tschapek, N. Infed, S. H. J. Smits, R. Ernst, L. Schmitt, *Anal. Biochem.* **2011**, 408, 64-70.

[70] S. Wang, S. Yada, T. Yoshimura, *Langmuir* **2024**, 40, 27028-27039.
